# Ag/Pyridine Co-Mediated Oxidative Arylthiocyanation of Activated Alkenes

**DOI:** 10.3390/molecules23102727

**Published:** 2018-10-22

**Authors:** De-Long Kong, Jian-Xun Du, Wei-Ming Chu, Chun-Ying Ma, Jia-Yi Tao, Wen-Hua Feng

**Affiliations:** Department of New Drug Research and Development, Institute of Materia Medica, Peking Union Medical College & Chinese Academy of Medical Sciences, Beijing 100050, China; kongdl@iccas.ac.cn (D.-L.K.); dujianxun@imm.ac.cn (J.-X.D.); chuweiming@imm.ac.cn (W.-M.C.); machunying@imm.ac.cn (C.-Y.M.); taojiayi@imm.ac.cn (J.-Y.T.)

**Keywords:** arylthiocyanation, difunctionalization, radical, oxindole, synthetic methods

## Abstract

An efficient Ag/pyridine co-mediated oxidative arylthiocyanation of activated alkenes via radical addition/cyclization cascade process was developed. This reaction could be carried out under mild conditions to provide biologically interesting 3-alkylthiocyanato-2-oxindoles in good to excellent yields. Mechanistic studies suggested a unique NCS• radical addition path and clarified the dual roles of catalytic pyridine as base and crucial ligand to accelerate the oxidation of Ag(I) to Ag(II), which is likely oxidant responsible for the formation of NCS• radical. These mechanistic results may impact the design and refinement of other radical based reactions proceeding through catalytic oxidations mediated by Ag(I)-pyridine/persulfate. The chemical versatility of thiocyanate moiety was also highlighted via SCN-tailoring chemistry in post-synthetic transformation for new S-C(sp^3^/sp^2^/sp), S-P, and S-S bonds constructions. The protocol provides an easy access to many important bioisosteres in medicinal chemistry and an array of sulfur-containing 2-oxindoles that are difficult to prepare by other approaches.

## 1. Introduction

**Figure 1:**
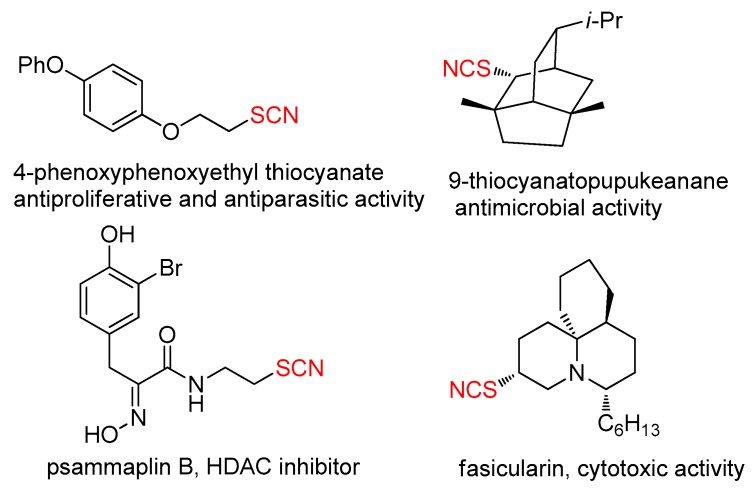
Representative bioactive alkyl thiocyanates.

Alkyl thiocyanates constitutes a key structural feature of a vast number of natural products and pharmaceuticals with a broad spectrum of biological activities (Figure 1). For example, the compound 4-phenoxyphenoxyethyl thiocyanate possesses great antiproliferative and antiparasitic activity [1]. The natural products psammaplin B, 9-thiocyanatopupukeanane, and fasicularin have been evaluated as a histone deacetylase (HDAC) enzyme inhibitor, antimicrobial agent, and cytotoxic agent, respectively [2,3,4]. Further, alkyl thiocyanates also serve as safe cyanating agents and versatile synthetic intermediates for the assembly of functionalized heterocycles and sulfur-based compounds [5,6,7,8,9]. Therefore, their synthetic importance has prompted considerable interest in developing operative construction methodologies for this motif.

**Scheme 1:**
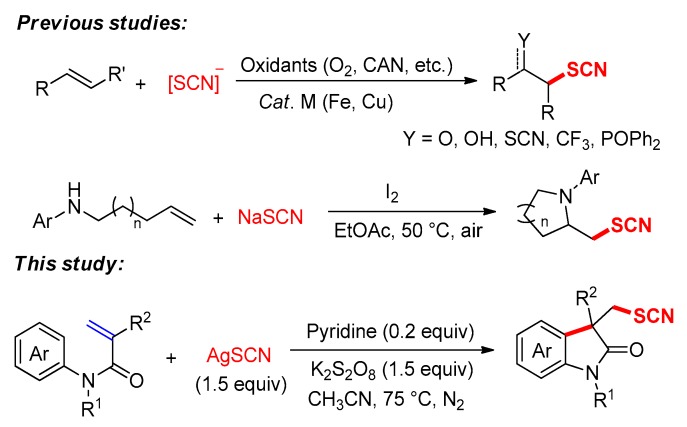
Difunctionalization of alkenes with thiocyanation.

So far, the reported approaches for the preparation of alkyl thiocyanates mainly focused on the nucleophilic [10,11,12,13] and electrophilic [14,15,16] substitution of prefunctionalized alkyl substrates with appropriated thiocyanation reagent and the direct thiocyanation of alkyl C–H bonds via oxidative functionalization [17,18,19,20,21,22,23]. However, in these transformations, only a C–S bond is formed. In contrast, the 1,2-difunctionalization of alkenes comprising a cascade thiocyanation and β-functionalization is more appealing from a synthetic perspective because of its high economy of steps and atoms. In recent years, many metal-free and transition-metal-catalyzed transformations such as thiocyanooxygenation [24,25,26,27], dithiocyanation [28,29], thiocyanophosphinoylation [30], thiotrifluoromethylation [31], and thiocyanoamination [32] have been developed (Scheme 1). In spite of these significant advances, arylthiocyanation of alkenes via 1,2-difunctionalization remains relatively unexplored. Especially, the cascade oxidative coupling/cyclization of functionalized alkenes with thiocyanate salts to access structurally diverse heterocycles, which is one of the most important and promising areas in synthetic and medicinal chemistry, are still far less developed. Herein, we would like to report the silver-mediated oxidative arylthiocyanation of alkenes via a radical addition/cyclization cascade process.

Considering the ubiquitous existence and unique biological activity of 2-oxindoles, *N*-arylacrylamides were chosen as the platform to realize C–H functionalization and C–S and C–C bond formation in one pot to produce the valuable 3-alkylthiocyanato-2-oxindoles. During the course of our studies, Chen and co-workers reported a very similar method which required not only an elevated temperatures (100 °C), but a large excess of oxidants and stoichiometric amounts of strong base Cs_2_CO_3_ [33], while, we found that only a slightly excess of the oxidants and catalytic amount of pyridine already could promote the C-H thiocyanation of *N*-arylacrylamides at 75 °C, making such a protocol easy to handle and scalable. Notably, in contrast to other oxindole syntheses, the resulting products, substituted 2-oxindoles with an appended SCN group, can be used to create further molecular complexity and diversity around the privileged scaffold of 2-oxindoles or can be applied for bioorthogonal transformation under physiological conditions [34,35,36,37].

## 2. Results and Discussion

As an easily prepared and stable thiocyanation reagent, AgSCN has been previously used as a SCN radical source [38]. Our study commenced by examining the arylthiocyanation of *N*-methyl-*N*-phenylmethacrylamide **1a** in the presence of AgSCN (1.5 equiv) in CH_3_CN at 75 °C (Table 1). The oxidant was found to be crucial for this reaction. As an example, ceric ammonium nitrate (CAN) only afforded the product **2a** in 15% yield, while di-*tert*-butyl peroxide (DTBP), oxone, PhI(OAc), and Selectfluor completely shut down the reaction (entry 1–5). Peroxydisulfate ion (S_2_O_8_^2−^, oxidation potential is 2.01 V) have been proven to be a powerful inorganic oxidant in oxidizing Ag(I) to the metastable Ag(II) species (1.98 V), which is the key step in most Ag(I)-mediated oxidative processes [39]. However, no desired product was observed with single K_2_S_2_O_8_ (entry 6). We envisioned that a base could be necessary to capture the proton released in this reaction; thus, several bases were next investigated. However, NaHCO_3_, Cs_2_CO_3_, hexamethylphosphoramide (HMPA) and Et_3_N all failed to give the desired reaction (entry 7–10). 1,8-Diazabicyclo[5,4,0]undec-7-ene (DBU) slightly increased the yield to 55% (entry 11). Pleasingly, the addition of 1 equiv pyridine significantly improved the efficiency of the reaction and provided **2a** in 83% yield (entry 12). Moreover, the reaction can be performed well when catalytic amount pyridine (0.2 equiv) was used, giving **2a** in 85% yield, albeit with a longer reaction time (entry 13). As a control experiment, removing K_2_S_2_O_8_ led to no reaction (entry 15).

**Scheme 2:**
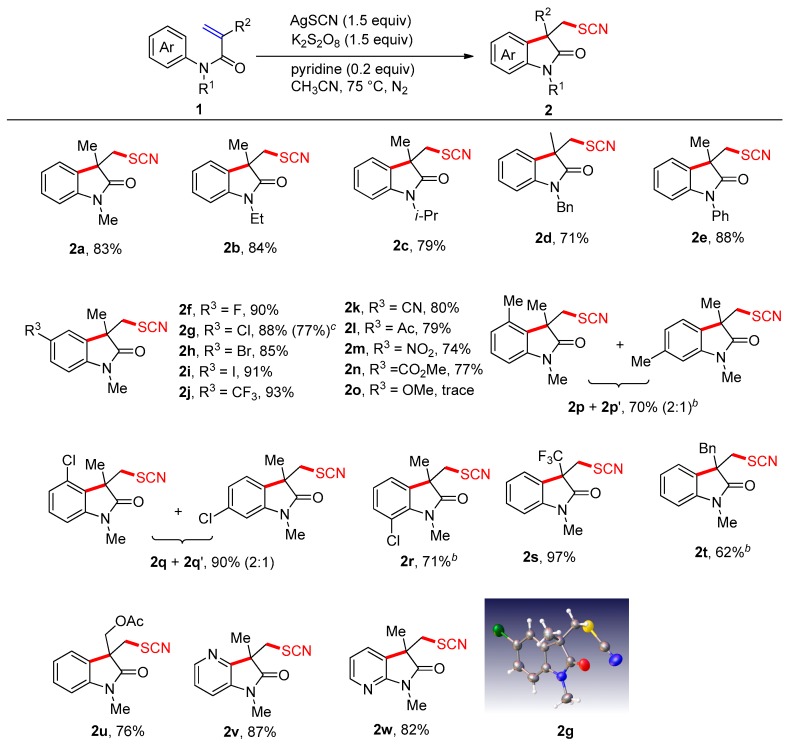
Scope of Activated Alkenes ^a^. ^a^ *Reaction conditions*: **1a** (0.2 mmol, 1 equiv), AgSCN (1.5 equiv), K_2_S_2_O_8_ (1.5 equiv), pyridine (0.2 equiv) in CH_3_CN (2.5 mL) at 75 °C for 8 h. ^b^ 0.5 equiv portion pyridine was used. ^c^ The yield of gram scale reaction (5 mmol) is given in parenthesis.

With the optimized conditions in hand, we then investigated the substrate scope of silver-mediated arylthiocyanation of alkenes (Scheme 2). Several *N*-substituted substrates bearing methyl, ethyl, isopropyl, phenyl and benzyl were tolerated, affording the desired products **2a**–**e** in good yields. Next, the effect of the substituent in the phenyl ring of *N*-arylacrylamides was studied. In general, very smooth arylthiocyanation occurred for *N*-arylacrylamides having substituents at the *para* and *meta* as well as *ortho* positions in the aniline. Substrates with halo-substituents (F, Cl, Br, and I) on the *para*-position of the *N*-aryl moiety performed well to produce the thiocyanated 2-oxindoles **2f**–**i** in high yields (85–91%), which offers the potential for further derivatization via cross-coupling reactions. Other electron withdrawing groups such as CF_3_, CN, Ac, and CO_2_Me on the *para*-position of aryl rings were also compatible, affording the corresponding products **2j**–**n** in good to excellent yields (77–93%). In particular, the *para*-NO_2_ substituted substrate which is usually inert in radical cyclizations [40], afforded the desired oxindole **2m** still with high yields. An obvious negative electronic effect from 4-methoxy on the *para*-position of the aryl moiety led to only a trace amount of the desired product **2o** as determined by LC-MS analysis. While *meta*-Substituted *N*-phenylacrylamides with both electron-withdrawing and electron-donating groups could be cyclized smoothly to give a mixture of regioisomers in a good yield (**2p**–**q**, ratio = 2:1). To our satisfaction, the *ortho*-position substituted *N*-arylacylamides, which usually did not work well in radical cyclizations due to steric effect, afforded the corresponding oxindoles **2r** still with good yields. Next, the compatibility of the substituents on the *α*-position (R^2^) of the acrylamides was also investigated. Several *α*-substituents, including CF_3_, benzyl and ester, were tolerated in the reaction to furnish the desired products **2s**–**u** in good yields. Notably, the reaction can also be carried out successfully with heterocyclic substrates to furnish biologically interesting heterocyclic scaffolds, 7-azaoxindole **2v** and 4-azaoxindole **2w** in high yields. To document the potential of the method, a gram scale (5 mmol) reaction was performed for the generation of compound **2g**. The reaction worked smoothly, giving the corresponding product in 77% yield. The molecular structure of **2g** was confirmed by the X-ray crystal analysis (CCDC 1849159, see the Supplementary Materials for details).

**Scheme 3:**
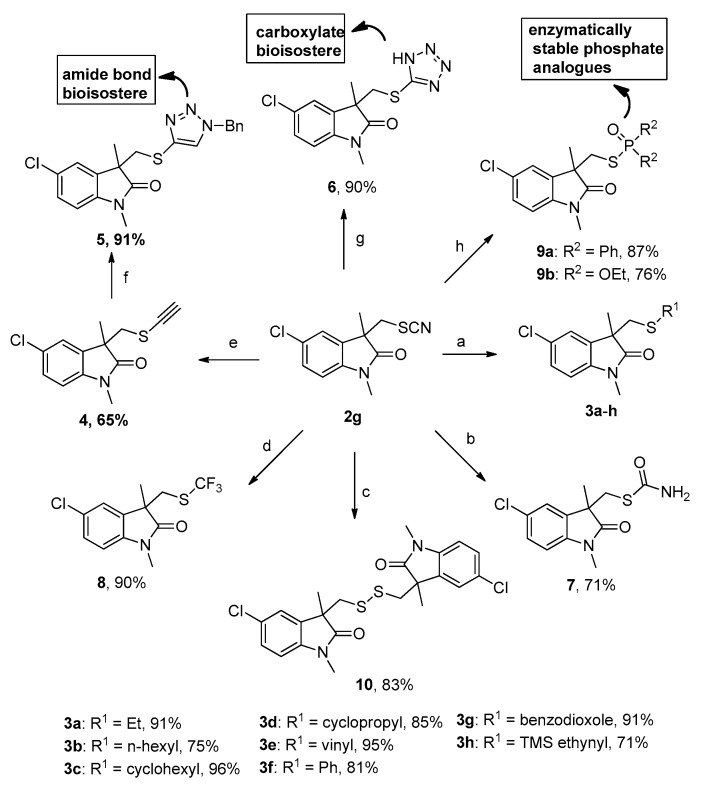
Derivatizations of **2g** via SCN-Tailoring Chemistry ^a^. ^a^ *Reaction conditions*: (a) RMgBr or TMSC≡CLi, THF, 0 °C–rt, 2 h; (b) H_2_SO_4_, rt, 2 h; (c) Et_2_NH, *n*-BuLi, 0 °C–rt, 3 h; (d) TMSCF_3_, CsF, CH_3_CN, rt, 2 h; (e) HC≡CMgCl, LiCl, THF, 0 °C–rt, 2 h; (f) BnN_3_, CuSO_4_, Na ascorbate, DCM/H_2_O = 3:1, rt, 1 h; (g) NaN_3_, ZnBr_2_, *i*-PrOH/H_2_O = 1:1, reflux; 1 h) H-P(O)(R^2^)_2_, DBU, toluene, rt, 3 h.

The 2-oxindole scaffold is a common motif for many biologically active natural products and pharmaceuticals. 2-Oxindoles with an appended SCN moiety obtained by the presented methodology can be used to create a focused compound library. Therefore, we attempted to explore the post-synthetic transformations of the SCN moiety (Scheme 3). Firstly, an array of unsymmetrical thioethers **3a**–**h** could be efficiently prepared starting from **2g** with corresponding Grignard reagent or lithium reagent. In this type of transformation, new C–S bonds that involve C_sp3_, C_sp2_, and C_sp_ were constructed, demonstrating the generality of this homologation chemistry. In a particularly noteworthy example, treatment of **2g** with ethynylmagnesium chloride/LiCl led cleanly to thioalkyne **4**, which was used for further modification through copper-catalyzed azide–thioalkyne cycloadditions (CuAtAC) to generate thiotriazole motif **5**, an important amide bond bioisostere in medicinal chemistry. In addition, cycloaddition of **2g** with NaN_3_ led to the generation of thiotetrazole **6**, itself a carboxylate bioisotere and important structural motifs existed in a lot of cephalosporins drugs. The hydrolysis reaction of **2g** with sulfuric acid furnished thiocarbamate **7** in good yield. Furthermore, the valuable trifluoromethylthiole-containing oxindole **8** was achieved upon treatment of **2g** with TMSCF_3_ and CsF, providing a facile method for accessing the interesting trifluoromethylthiole-containing oxindoles. Notably, biologically highly relevant phosphonothioates (**9a**–**b**), which have been identified as enzymatically stable phosphate analogues, could also be easy prepared via base promoted nucleophilic substitution of H-phosphine oxides and H-phosphinates on thiocyanates. Clearly, this reaction provides an easily access to S-P(O) bond-containing 2-oxindoles, which have never been explored before. Finally, symmetrical disulfide **10** was prepared in the presence of a base via reductive dimerization of **2g**. The base might promote the formation of a nucleophilic thiolate anion which could attack another thiocyanate to give symmetrical disulfide. It is worth pointing out that the aryl chloride functionality of **2g** remains totally untouched in all of these derivatization reactions, which enable further elaboration via cross-coupling chemistry. These representative transformations clearly demonstrate the potential molecular diversity that can be created starting from the SCN appended oxindoles.

To gain insight into mechanistic details of this reaction, several control experiments were performed (see the Supplementary Materials for details). The desired transformation was completely inhibited when 1 equiv 2,2,6,6-tetramethylpiperidine-1-oxyl (TEMPO) or 2,6-di-*tert*-butyl-4-methylphenol (BHT, a well-known radical scavenger) was added into the present reaction system. Replacement of AgSCN with CuSCN or KSCN in the standard reaction conditions led to no product, while AgNO_3_/KSCN gave a yield of 15%, which indicated silver was crucial to this transformation to understand the role of pyridine in this reaction, a stage reaction was carried out. In this experiment, we found that stirring of AgSCN/K_2_S_2_O_8_/pyridine mixture in CH_3_CN at 75 °C for 1 h led to a formation of catalytic silver–pyridine complexes [41,42], which could react with **1a** without free pyridine to give **2a** in 68% yield. This experiment suggested pyridine functioned not only as a base but also as a possible ligand to accelerate the oxidation of Ag(I) to Ag(II), which was consistent with Bonchev and Aleksiev’s reports that the addition of a suitable nitrogen-containing neutral ligand to Ag(I)/persulfate reactions resulted in a lowering of the potential of the Ag(II)/Ag(I) couple [43,44]. Furthermore, Ag(py)_4_S_2_O_8_ was prepared as an orange solid. Then, treatment of **1a** with Ag(py)_4_S_2_O_8_ (0.5 equiv), KSCN(1.5 equiv), and K_2_S_2_O_8_ (1.5 equiv) in CH_3_CN at 75 °C for 2 h led to the formation of **2a** in 56% isolated yield. In this experiment, a quick reduction of orange-Ag(II) to colorless-Ag(I) was observed at the beginning of the reaction which suggested that Ag(II) should be the active species to oxidize NCS^−^ to NCS• radical.

**Scheme 4:**
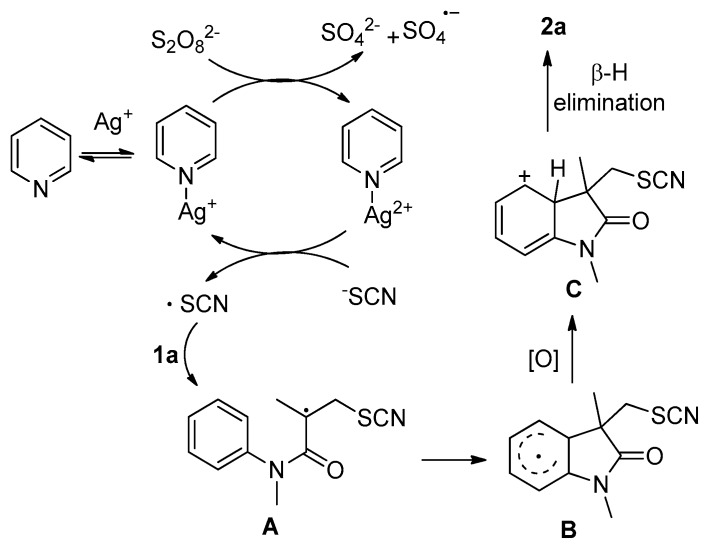
Proposed mechanism.

On the basis of the experimental results and previous reports [45,46,47,48,49,50], a plausible mechanism is proposed as depicted in Scheme 4. Pyridine coordination to Ag(I) is followed by persulfate oxidation, resulting a Ag(II)–pyridine complex. The oxidation of the thiocyanate anion by Ag(II)–pyridine complex generates an electrophilic SCN radical, which then attacks the C=C bond of **1a** to afford corresponding alkyl radical intermediate **A**, followed by cyclization to give the aryl radical **B**. Another single-electron transfer from intermediate **B** to an additional 1 equiv of Ag(II) generates intermediate **C**, which affords product **2a** by *β*-H elimination.

## 3. Materials and Methods

### 3.1. General Information

All reagents used were obtained commercially and used without further purification unless indicated otherwise. Column chromatography was carried out on silica gel (300–400 grad). TLC analysis was performed on pre-coated, glass-backed silica gel plates and visualized with UV light. ^1^H-NMR and ^13^C-NMR spectra were obtained on a 400 MHz (Varian, Palo Alto, CA, USA) and 500 MHz (Bruker-Biospin, Billerica, MA, USA) NMR spectrometer in the deuterated solvents and chemical shifts are reported in ppm form with tetramethylsilane as the internal standard. Multiplicities are indicated as s (singlet), d (doublet), t (triplet), q (quintet), and m (multiplet), and coupling constants (*J*) are reported in Hertz. High resolution mass spectra (HRMS) is recorded using electrospray ionization (ESI) (Thermo Scientific, TSQ Fortis, MA, USA) equipped with a quadrupole mass analyzer. X-ray structures were determined on an X-ray diffraction meter (Bruker-Biospin, Billerica, MA, USA). Melting points were measured on a Beijing Tech X-4 apparatus without correction (Tech X-4, Beijing, China). All the substrates **1** were synthesized according to the literature, and the NMR spectroscopy were consisted with reported data [51].

### 3.2. General Procedure for Arylthiocyanation of Acrylamides ***1***

Acrylamides 1 (0.2 mmol), K_2_S_2_O_8_ (81 mg, 0.3 mmol) and AgSCN (50 mg, 0.3 mmol) were combined in an oven-dried sealed tube. The vessel was evacuated and backfilled with N_2_ (this process was repeated a total of 3 times), and CH_3_CN (3 mL) and pyridine (3.5 μL, 0.04 mmol) were added via syringe. The tube was then sealed with a Teflon lined cap and placed into a preheated oil bath at 75 °C with vigorous stirring. After 8 h, the reaction mixture was cooled to room temperature and filtered through a plug of silica (eluted with EtOAc). The filtrate was concentrated, and the product was purified by column chromatography on silica gel (petroleum ether/EtOAc) to give product **2**.

*1,3-Dimethyl-3-(thiocyanatomethyl)indolin-2-one* (**2a**). Yield 38 mg (83%), colorless oil. ^1^H-NMR (400 MHz, CDCl_3_) δ 7.38 (td, *J* = 7.7, 1.3 Hz, 1H), 7.29 (m, 1H), 7.15 (td, *J* = 7.6, 1.0 Hz, 1H), 6.92 (d, *J* = 7.8 Hz, 1H), 3.45–3.33 (m, 2H), 3.26 (s, 3H), 1.51 (s, 3H). ^13^C-NMR (100 MHz, CDCl_3_) δ 177.4, 143.5, 129.9, 129.5, 123.6, 123.3, 111.6, 108.8, 48.6, 40.8, 26.5, 22.7. HRMS (ESI): calcd. for C_12_H_13_N_2_OS ([M + H]^+^) 233.0749, found 233.0739.

*1-Ethyl-3-methyl-3-(thiocyanatomethyl)indolin-2-one* (**2b**). Yield 42 mg (84%), colorless oil. ^1^H-NMR (400 MHz, CDCl_3_) δ 7.35 (td, *J* = 7.8, 1.3 Hz, 1H), 7.30–7.23 (m, 1H), 7.12 (td, *J* = 7.6, 1.0 Hz, 1H), 6.92 (d, *J* = 7.8 Hz, 1H), 3.96–3.67 (m, 2H), 3.48–3.31 (m, 2H), 1.48 (s, 3H), 1.28 (t, *J* = 7.2 Hz, 3H). ^13^C-NMR (100 MHz, CDCl_3_) δ177.0, 142.6, 130.1, 129.4, 123.7, 123.1, 111.6, 108.9, 48.5, 40.9, 35.1, 22.8, 12.7. HRMS (ESI): calcd. for C_13_H_15_N_2_OS ([M + H]^+^) 247.0899, found 247.0898.

*1-Isopropyl-3-methyl-3-(thiocyanatomethyl)indolin-2-one* (**2c**). Yield 41 mg (79%), colorless oil. ^1^H-NMR (400 MHz, CDCl_3_) δ 7.33 (td, *J* = 7.7, 1.5 Hz, 1H), 7.26 (dd, *J* = 8.0, 1.7 Hz, 1H), 7.10 (t, *J* = 7.5 Hz, 1H), 7.07 (d, *J* = 8.0 Hz, 1H), 4.63 (dt, *J* = 14.2, 7.1 Hz, 1H), 3.45–3.27 (m, 2H), 1.50 (dd, *J* = 7.0, 4.0 Hz, 6H), 1.46 (s, 3H). ^13^C-NMR (100 MHz, CDCl_3_) δ 177.1, 142.3, 130.3, 129.2, 123.8, 122.7, 111.6, 110.5, 48.3, 44.4, 41.1, 22.9, 19.5, 19.5. HRMS (ESI): calcd. for C_14_H_17_N_2_OS ([M + H]^+^) 261.1056, found 261.1054.

*1-Benzyl-3-methyl-3-(thiocyanatomethyl)indolin-2-one* (**2d**). Yield 44 mg (71%), colorless oil.^1^H-NMR (400 MHz, CDCl_3_) δ 7.36–7.23 (m, 7H), 7.17–7.05 (m, 1H), 6.81 (dq, *J* = 1.5, 0.7 Hz, 1H), 5.05–4.86 (m, 2H), 3.45 (q, *J* = 9.3 Hz, 2H), 1.55 (s, 3H). ^13^C-NMR (100 MHz, CDCl_3_) δ 177.6, 142.6, 135.5, 129.8, 129.4, 128.9, 127.9, 127.4, 123.6, 123.3, 111.6, 109.9, 48.7, 44.2, 40.8, 23.2. HRMS (ESI): calcd. for C_18_H_17_N_2_OS ([M + H]^+^) 309.1056, found 309.1052.

*3-Methyl-1-phenyl-3-(thiocyanatomethyl)indolin-2-one* (**2e**). Yield 52 mg (88%), white solid. ^1^H-NMR (400 MHz, CDCl_3_) δ 7.58–7.51 (m, 2H), 7.46–7.40 (m, 3H), 7.37–7.27 (m, 2H), 7.19 (dt, *J* = 7.6, 1.1 Hz, 1H), 6.89 (td, *J* = 1.4, 0.7 Hz, 1H), 3.55–3.43 (m, 2H), 1.63 (s, 3H). ^13^C-NMR (100 MHz, CDCl_3_) δ 177.0, 143.6, 134.1, 129.8, 129.6, 129.4, 128.5, 126.6, 123.8, 123.8, 111.4, 110.2, 48.9, 41.2, 23.2. HRMS (ESI): calcd. for C_17_H_15_N_2_OS ([M + H]^+^) 295.0899, found 295.0896.

*5-Fluoro-1,3-dimethyl-3-(thiocyanatomethyl)indolin-2-one* (**2f**). Yield 45 mg (90%), white solid. ^1^H-NMR (400 MHz, CDCl_3_) δ 7.16–6.95 (m, 1H), 6.85 (dd, *J* = 8.4, 4.1 Hz, 1H), 3.37 (s, 1H), 3.25 (s, 2H), 1.50 (s, 2H).^13^C-NMR (100 MHz, CDCl_3_) δ 177.1, 159.6 (d, *J* = 241.1 Hz), 139.5, 131.5 (d, *J* = 8 Hz), 115.8 (d, *J* = 23.5 Hz), 111.9 (d, *J* = 24.8 Hz), 111.2, 109.4 (d, *J* = 8.1 Hz), 49.1, 40.5, 26.7, 22.7. HRMS (ESI): calcd. for C_12_H_12_N_2_OFS ([M + H]^+^) 251.0648, found 251.0654.

*5-Chloro-1,3-dimethyl-3-(thiocyanatomethyl)indolin-2-one* (**2g**). Yield 47 mg (88%), pale yellow solid. ^1^H-NMR (400 MHz, CDCl_3_) δ 7.35 (dd, *J* = 8.3, 2.1 Hz, 1H), 7.32–7.26 (m, 1H), 6.85 (d, *J* = 8.3 Hz, 1H), 3.37 (s, 2H), 3.25 (s, 3H), 1.51 (s, 3H). ^13^C-NMR (100 MHz, CDCl_3_) δ 176.9, 142.1, 131.6, 129.4, 128.7, 124.2, 111.2, 109.8, 48.9, 40.5, 26.7, 22.7. HRMS (ESI): calcd. for C_12_H_12_N_2_OClS ([M + H]^+^) 267.0353, found 267.0354.

*5-Bromo-1,3-dimethyl-3-(thiocyanatomethyl)indolin-2-one* (**2h**). Yield 52 mg (85%), pale yellow solid. ^1^H-NMR (400 MHz, CDCl_3_) δ 7.50 (dd, *J* = 8.3, 2.0 Hz, 1H), 7.40 (d, *J* = 1.9 Hz, 1H), 6.80 (d, *J* = 8.3 Hz, 1H), 3.37 (d, *J* = 1.0 Hz, 2H), 3.24 (s, 3H), 1.51 (s, 3H). ^13^C-NMR (100 MHz, CDCl_3_) δ 176.8, 142.56, 132.3, 132.0, 126.9, 115.9, 111.2, 110.2, 48.8, 40.5, 26.7, 22.7. HRMS (ESI): calcd. for C_12_H_12_N_2_OBrS ([M + H]^+^) 310.9848, found 310.9856.

*5-Iodo-1,3-dimethyl-3-(thiocyanatomethyl)indolin-2-one* (**2i**). Yield 65 mg (91%), pale yellow solid. ^1^H-NMR (400 MHz, CDCl_3_) δ 7.69 (dd, *J* = 8.2, 1.7 Hz, 1H), 7.56 (d, *J* = 1.7 Hz, 1H), 6.70 (d, *J* = 8.2 Hz, 1H), 3.36 (d, *J* = 1.3 Hz, 2H), 3.23 (s, 3H), 1.49 (s, 3H). ^13^C-NMR (100 MHz, CDCl_3_) δ 176.6, 143.3, 138.3, 132.4, 132.4, 111.2, 110.8, 85.6, 48.7, 40.5, 26.6, 22.7. HRMS (ESI): calcd. for C_12_H_12_N_2_OIS ([M + H]^+^) 358.9709, found 358.9710.

*1,3-Dimethyl-3-(thiocyanatomethyl)-5-(trifluoromethyl)indolin-2-one* (**2j**). Yield 56 mg (93%), pale yellow solid. ^1^H-NMR (400 MHz, CDCl_3_) δ 7.72–7.64 (m, 1H), 7.52 (d, *J* = 1.8 Hz, 1H), 7.01 (d, *J* = 8.2 Hz, 1H), 3.40 (s, 2H), 3.30 (s, 3H), 1.54 (s, 3H). ^13^C-NMR (100 MHz, CDCl_3_) δ 177.3, 146.5, 130.5, 127.3, 127.3, 125.8, 125.5, 120.8, 120.8, 110.9, 108.6, 48.7, 40.3, 26.8, 22.7. HRMS (ESI): calcd. for C_13_H_12_N_2_OF_3_S ([M + H]^+^) 301.0617, found 301.0616.

*1,3-Dimethyl-2-oxo-3-(thiocyanatomethyl)indoline-5-carbonitrile* (**2k**). Yield 41 mg (80%), white solid. ^1^H-NMR (400 MHz, CDCl_3_) δ 7.70 (dd, *J* = 8.2, 1.6 Hz, 1H), 7.54 (d, *J* = 1.6 Hz, 1H), 6.99 (d, *J* = 8.2 Hz, 1H), 3.38 (s, 2H), 3.29 (s, 3H), 1.52 (s, 3H). ^13^C-NMR (100 MHz, CDCl_3_) δ 177.1, 147.4, 134.7, 131.1, 127.1, 118.8, 110.7, 109.3, 106.6, 48.7, 40.1, 26.8, 22.7. HRMS (ESI): calcd. for C_13_H_12_N_3_OS ([M + H]^+^) 258.0695, found 258.0699.

*5-Acetyl-1,3-dimethyl-3-(thiocyanatomethyl)indolin-2-one* (**2l**). Yield 43 mg (79%), pale yellow solid. ^1^H-NMR (400 MHz, CDCl_3_) δ 8.02 (dd, *J* = 8.2, 1.8 Hz, 1H), 7.91 (d, *J* = 1.7 Hz, 1H), 6.97 (d, *J* = 8.2 Hz, 1H), 3.41 (s, 2H), 3.30 (s, 3H), 2.59 (s, 3H), 1.52 (s, 3H). ^13^C-NMR (100 MHz, CDCl_3_) δ 196.6, 177.7, 147.8, 132.7, 131.3, 130.3, 123.6, 111.1, 108.3, 48.6, 40.3, 26.8, 26.5, 22.8. HRMS (ESI): calcd. for C_14_H_15_N_2_O_2_S ([M + H]^+^) 275.0848, found 275.0844.

*1,3-Dimethyl-5-nitro-3-(thiocyanatomethyl)indolin-2-one* (**2m**). Yield 41 mg (74%), white solid. ^1^H-NMR (400 MHz, CDCl_3_) δ 8.36 (dd, *J* = 8.7, 2.2 Hz, 1H), 8.20 (d, *J* = 2.2 Hz, 1H), 7.03 (d, *J* = 8.7 Hz, 1H), 3.43 (s, 2H), 3.34 (s, 3H), 1.57 (s, 3H). ^13^C-NMR (100 MHz, CDCl_3_) δ 177.5, 149.2, 143.9, 130.7, 126.7, 119.7, 110.5, 108.5, 48.9, 39.9, 27.0, 22.8. HRMS (ESI): calcd. for C_12_H_12_N_3_O_3_S ([M + H]^+^) 278.0593, found 278.0581.

*Methyl 1,3-dimethyl-2-oxo-3-(thiocyanatomethyl)indoline-5-carboxylate* (**2n**). Yield 44 mg (77%), white solid. ^1^H-NMR (400 MHz, CDCl_3_) δ 8.10 (dd, *J* = 8.2, 1.7 Hz, 1H), 7.94 (d, *J* = 1.9 Hz, 1H), 6.95 (d, *J* = 8.0 Hz, 1H), 3.89 (s, 3H), 3.39 (s, 2H), 3.28 (s, 3H), 1.51 (s, 3H).^13^C-NMR (100 MHz, CDCl_3_) δ 177.7, 166.5, 147.6, 132.1, 130.0, 125.3, 124.8, 111.0, 108.4, 52.3, 48.5, 40.4, 26.8, 22.7. HRMS (ESI): calcd. for C_14_H_14_N_2_O_3_SNa ([M + Na]^+^) 313.0617, found 313.0620.

*1,3,4-Trimethyl-3-(thiocyanatomethyl)indolin-2-one* (**2p**).Total yield 34 mg (70%, **2p**/**2p**’ = 2:1). ^1^H-NMR (400 MHz, CDCl_3_) δ 7.31–7.26 (m, 1H), 6.93–6.89 (m, 1H), 6.77 (d, *J* = 7.8 Hz, 1H), 3.67–3.44 (m, 2H), 3.25 (s, 3H), 2.41 (s, 3H), 1.56 (s, 3H). ^13^C-NMR (100 MHz, CDCl_3_) δ 177.5, 144.0, 135.2, 129.4, 125.8, 123.7, 111.0, 106.5, 49.6, 38.6, 26.6, 21.5, 18.4. HRMS (ESI): calcd. for C_13_H_15_N_2_OS ([M + H]^+^) 247.0899, found 247.0898.

*1,3,6-Trimethyl-3-(thiocyanatomethyl)indolin-2-one* (**2p′**). Total yield 34 mg (70%, **2p**/**2p**′ = 2:1). ^1^H-NMR (400 MHz, CDCl_3_) δ 7.16 (d, *J* = 7.5 Hz, 1H), 6.95 (ddd, *J* = 7.6, 1.5, 0.8 Hz, 1H), 6.75–6.73 (m, 1H), 3.38 (s, 2H), 3.24 (s, 3H), 2.41 (d, *J* = 0.7 Hz, 3H), 1.48 (s, 3H). ^13^C-NMR (100 MHz, CDCl_3_) δ 177.7, 143.5, 139.8, 127.0, 126.6, 123.3, 111.78, 109.7, 48.40, 41.0, 26.5, 22.8, 22.0. HRMS (ESI): calcd. for C_13_H_15_N_2_OS ([M + H]^+^) 247.0899, found 247.0898.

*4-Chloro-1,3-dimethyl-3-(thiocyanatomethyl)indolin-2-one* (**2q**). Total yield 48 mg (90%, **2q**/**2q**’ = 2:1). ^1^H-NMR (400 MHz, CDCl_3_) δ 7.33 (t, *J* = 8.0 Hz, 1H), 7.08 (d, *J* = 8.2 Hz, 1H), 6.84 (d, *J* = 7.7 Hz, 1H), 3.85 (d, *J* = 13.5 Hz, 1H), 3.41 (d, *J* = 13.3 Hz, 1H), 3.27 (s, 3H), 1.62 (s, 3H). ^13^C-NMR (100 MHz, CDCl_3_) δ 176.9, 145.5, 131.5, 130.8, 124.6, 124.1, 110.5, 107.4, 50.52, 37.08, 26.80, 20.91. HRMS (ESI): calcd. for C_12_H_12_N_2_OClS ([M + H]^+^) 267.0353, found 267.0354.

*6-Chloro-1,3-dimethyl-3-(thiocyanatomethyl)indolin-2-one* (**2q**′). Total yield 48 mg (90%, **2q**/**2q**′ = 2:1). ^1^H-NMR (400 MHz, CDCl_3_) δ 7.21 (d, *J* = 7.8 Hz, 1H), 7.12 (dd, *J* = 8.0, 1.9 Hz, 1H), 6.93 (d, *J* = 2.0 Hz, 1H), 3.37 (s, 2H), 3.24 (s, 3H), 1.49 (s, 3H). ^13^C-NMR (100 MHz, CDCl_3_) δ 177.4, 144.7, 135.3, 128.3, 126.0, 123.1, 111.4, 109.6, 48.5, 40.5, 26.7, 22.8. HRMS (ESI): calcd. for C_12_H_12_N_2_OClS ([M + H]^+^) 267.0353, found 267.0354.

*7-Chloro-1,3-dimethyl-3-(thiocyanatomethyl)indolin-2-one* (**2r**). Yield 38 mg (71%), white solid. ^1^H-NMR (400 MHz, CDCl_3_) δ 7.29 (dd, *J* = 8.2, 1.2 Hz, 1H), 7.17 (dd, *J* = 7.4, 1.2 Hz, 1H), 7.05 (dd, *J* = 8.2, 7.4 Hz, 1H), 3.62 (s, 3H), 3.37 (s, 2H), 1.49 (s, 3H). ^13^C-NMR (100 MHz, CDCl_3_) δ 177.7, 139.5, 132.6, 131.8, 124.0, 122.1, 116.3, 111.3, 48.5, 40.7, 29.9, 23.1. HRMS (ESI): calcd. for C_12_H_12_N_2_OClS ([M + H]^+^) 267.0353, found 267.0354.

*1-Methyl-3-(thiocyanatomethyl)-3-(trifluoromethyl)indolin-2-one* (**2s**). Yield 55 mg (97%), white solid. ^1^H-NMR (400 MHz, CDCl_3_) δ 7.51 (td, *J* = 7.8, 1.2 Hz, 1H), 7.44 (dtd, *J* = 7.5, 1.2, 0.6 Hz, 1H), 7.22 (td, *J* = 7.6, 1.0 Hz, 1H), 6.98 (dt, *J* = 7.9, 0.8 Hz, 1H), 3.80–3.63 (m, 2H), 3.30 (s, 3H). ^13^C-NMR (100 MHz, CDCl_3_) δ 168.9, 145.0, 131.7, 126.0, 125.0, 123.9, 122.2, 120.5, 109.9, 109.4, 56.7 (q, *J* = 27.1 Hz), 34.3, 27.0. ^19^F NMR (376 MHz, CDCl_3_) δ −71.5. HRMS (ESI): calcd. for C_12_H_10_N_2_OF_3_S ([M + H]^+^) 287.0460, found 287.0456.

*3-Benzyl-1-methyl-3-(thiocyanatomethyl)indolin-2-one* (**2t**). Yield 38 mg (62%), colorless oil. ^1^H-NMR (400 MHz, CDCl_3_) δ 7.32–7.28 (m, 1H), 7.21 (ddd, *J* = 7.5, 1.3, 0.6 Hz, 1H), 7.15–7.06 (m, 4H), 6.88–6.83 (m, 2H), 6.69 (dt, *J* = 7.9, 0.8 Hz, 1H), 3.63–3.50 (m, 2H), 3.22–3.10 (m, 2H), 3.03 (s, 3H). ^13^C-NMR (100 MHz, CDCl_3_) δ 176.23, 144.1, 134.2, 130.0, 129.6, 129.2, 127.9, 127.2, 124.6, 122.8, 111.6, 108.6, 54.5, 43.0, 39.4, 26.2. HRMS (ESI): calcd. for C_18_H_17_N_2_OS ([M + H]^+^) 309.1056, found 309.1052.

*(1-Methyl-2-oxo-3-(thiocyanatomethyl)indolin-3-yl)methyl acetate* (**2u**). Yield 44 mg (76%), colorless oil. ^1^H-NMR (400 MHz, Acetone-*d*_6_) δ 7.52–7.48 (m, 1H), 7.40 (td, *J* = 7.8, 1.3 Hz, 1H), 7.14–7.07 (m, 2H), 4.55 (d, *J* = 11.0 Hz, 1H), 4.20 (d, *J* = 11.0 Hz, 1H), 3.82 (d, *J* = 13.4 Hz, 1H), 3.63 (d, *J* = 13.4 Hz, 1H), 3.23 (s, 3H), 1.92 (s, 3H). ^13^C-NMR (100 MHz, CDCl_3_) δ 174.3, 170.1, 144.12, 130.2, 126.0, 124.7, 123.4, 111.2, 109.0, 65.8, 52.2, 36.9, 26.7, 20.7. HRMS (ESI): calcd. for C_14_H_15_N_2_O_3_S ([M + H]^+^) 291.0798, found 291.0793.

*1,3-Dimethyl-3-(thiocyanatomethyl)-1H-pyrrolo[3,2-b]pyridin-2(3H)-one* (**2v**). Yield 40 mg (87%), colorless oil. ^1^H-NMR (400 MHz, CDCl_3_) δ 8.30 (dd, *J* = 5.1, 1.3 Hz, 1H), 7.28 (dd, *J* = 8.0, 5.2 Hz, 1H), 7.17 (dd, *J* = 8.0, 1.3 Hz, 1H), 3.58 (d, *J* = 13.3 Hz, 1H), 3.37 (d, *J* = 13.4 Hz, 1H), 3.28 (s, 3H), 1.54 (s, 3H). ^13^C-NMR (100 MHz, CDCl_3_) δ 176.0, 151.1, 143.6, 139.1, 124.0, 115.0, 110.6, 49.2, 38.7, 26.3, 21.5. HRMS (ESI): calcd. for C_11_H_12_N_3_OS ([M + H]^+^) 234.0695, found 234.0693.

*1,3-Dimethyl-3-(thiocyanatomethyl)-1H-pyrrolo[2,3-b]pyridin-2(3H)-one* (**2w**). Yield 38 mg (82%), colorless oil. ^1^H-NMR (400 MHz, CDCl_3_) δ 8.29 (ddd, *J* = 5.3, 1.6, 0.7 Hz, 1H), 7.57 (ddd, *J* = 7.3, 1.6, 0.7 Hz, 1H), 7.05 (ddd, *J* = 7.3, 5.3, 0.7 Hz, 1H), 3.46–3.32 (m, 5H), 1.53 (d, *J* = 0.7 Hz, 3H). ^13^C-NMR (100 MHz, CDCl_3_) δ 177.2, 156.9, 148.3, 131.5, 124.4, 118.7, 111.4, 48.7, 40.2, 25.7, 22.3. HRMS (ESI): calcd. for C_11_H_12_N_3_OS ([M + H]^+^) 234.0695, found 234.0693.

### 3.3. Procedure for Gram-Scale Preparation of 5-Chloro-1,3-dimethyl-3-(thiocyanatomethyl)indolin-2-one *(**2g**)*

Acrylamides **1g** (1.05g, 5 mmol), K_2_S_2_O_8_ (1.70 g, 7.5 mmol) and AgSCN (1.24 g, 7.5 mmol) were combined in an 50 mL oven-dried sealed tube. The vessel was evacuated and backfilled with N_2_ (this process was repeated a total of 3 times), and CH_3_CN (30 mL) and pyridine (79 μL, 1 mmol) were added via syringe. The tube was then sealed with a Teflon lined cap and placed into a preheated oil bath at 75 °C with vigorous stirring. After 12 h, the reaction mixture was cooled to room temperature and filtered through a plug of silica (eluted with EtOAc). The filtrate was concentrated, and the product was purified by column chromatography on silica gel (petroleum ether/EtOAc, 10:1) to give product **2g** (1.05g, 77%) as a yellow solid.

### 3.4. General Procedure for Preparation of Compounds ***3a**–**g***

To a solution of **2g** (53 mg, 0.2 mmol) in dry THF (3 mL) at 0 °C under N_2_ was added corresponding Grignard reagent RMgBr (0.6 mmol in THF, 3 equiv), dropwise, via syringe. The reaction was allowed to warm to room temperature, stirred for 2–3 h, whereupon TLC analysis indicated that the reaction was complete. Following quenching [saturated NH_4_Cl (aq)], and extraction (EtOAc, 2 x), the crude product was dried (Na_2_SO_4_), filtered and evaporation. Flash chromatography over silica gel (petroleum ether/EtOAc, 10:1) then yielded the corresponding product **3a**–**g**.

*5-Chloro-3-((ethylthio)methyl)-1,3-dimethylindolin-2-one* (**3a**). Yield 49 mg (91%), colorless oil. ^1^H-NMR (400 MHz, CDCl_3_) δ 7.24 (d, *J* = 6.6 Hz, 2H), 6.84–6.66 (m, 1H), 3.19 (s, 3H), 3.06–2.86 (m, 2H), 2.37 (qd, *J* = 7.3, 3.9 Hz, 2H), 1.39 (s, 3H), 1.11 (t, *J* = 7.4 Hz, 3H). ^13^C-NMR (100 MHz, CDCl_3_) δ 179.1, 142.2, 134.7, 128.2, 127.9, 123.7, 108.0, 49.5, 39.7, 27.9, 26.4, 22.9, 14.9. HRMS (ESI): calcd. for C_13_H_17_NOClS ([M + H]^+^) 270.0714, found 270.0712.

*5-Chloro-3-((hexylthio)methyl)-1,3-dimethylindolin-2-one* (**3b**). Yield 49 mg (75%), colorless oil. ^1^H-NMR (400 MHz, CDCl_3_) δ 7.29–7.25 (m, 2H), 6.78 (d, *J* = 8.8 Hz, 1H), 3.22 (s, 3H), 3.10–2.81 (m, 2H), 2.36 (td, *J* = 7.4, 1.5 Hz, 2H), 1.49–1.39 (m, 5H), 1.32–1.17 (m, 7H), 0.86 (t, *J* = 7.0 Hz, 3H). ^13^C-NMR (100 MHz, CDCl_3_) δ 179.1, 142.2, 134.8, 128.2, 128.0, 123.7, 109.0, 49.6, 40.1, 34.0, 31.4, 29.7, 28.4, 26.4, 22.9, 22.6, 14.1. HRMS (ESI): calcd. for C_17_H_25_NOClS ([M + H]^+^) 326.1345, found 326.1348.

*5-Chloro-3-((cyclohexylthio)methyl)-1,3-dimethylindolin-2-one* (**3c**). Yield 61 mg (96%), colorless oil. ^1^H-NMR (400 MHz, CDCl_3_) δ 7.32–7.24 (m, 2H), 6.77 (dd, *J* = 8.1, 0.6 Hz, 1H), 3.21 (s, 3H), 3.02 (d, *J* = 12.8 Hz, 1H), 2.88 (d, *J* = 12.8 Hz, 1H), 2.49–2.39 (m, 1H), 1.90–1.50 (m, 5H), 1.41 (s, 3H), 1.28–1.11 (m, 5H). ^13^C-NMR (100 MHz, CDCl_3_) δ 179.2, 142.1, 134.7, 128.1, 127.9, 123.7, 109.0, 49.4, 45.2, 38.0, 33.9, 33.6, 26.4, 26.1, 25.7, 22.8. HRMS (ESI): calcd. for C_17_H_23_NOClS ([M + H]^+^) 324.1183, found 324.1187.

*5-Chloro-3-((cyclopropylthio)methyl)-1,3-dimethylindolin-2-one* (**3d**). Yield 47 mg (85%), white solid. ^1^H-NMR (400 MHz, CDCl_3_) δ 7.29–7.24 (m, 2H), 6.80–6.76 (m, 1H), 3.23 (s, 3H), 3.04 (d, *J* = 1.2 Hz, 2H), 1.54 (tt, *J* = 7.3, 4.3 Hz, 1H), 1.43 (s, 3H), 0.79–0.67 (m, 2H), 0.49–0.33 (m, 2H). ^13^C-NMR (100 MHz, CDCl_3_) δ 179.1, 142.2, 134.8, 128.2, 127.9, 123.8, 109.0, 49.6, 41.6, 26.4, 23.1, 14.3, 9.5, 8.6. HRMS (ESI): calcd. for C_14_H_17_NOClS ([M + H]^+^) 282.0714, found 282.0717.

*5-Chloro-1,3-dimethyl-3-((vinylthio)methyl)indolin-2-one* (**3e**). Yield 51 mg (95%), colorless oil. ^1^H-NMR (400 MHz, CDCl_3_) δ 7.25 (d, *J* = 8.2 Hz, 2H), 6.75 (d, *J* = 8.2 Hz, 1H), 6.16 (dd, *J* = 16.9, 9.9 Hz, 1H), 5.10–4.99 (m, 2H), 3.18 (s, 3H), 3.09 (s, 2H), 1.43 (s, 3H). ^13^C-NMR (100 MHz, CDCl_3_) δ 178.6, 142.1, 134.2, 132.7, 128.4, 128.1, 123.9, 112.4, 109.1, 49.2, 40.1, 26.5, 22.7. HRMS (ESI): calcd. for C_13_H_15_NOClS ([M + H]^+^) 268.0557, found 268.0551.

*5-Chloro-1,3-dimethyl-3-((phenylthio)methyl)indolin-2-one* (**3f**). Yield 51 mg (81%), colorless oil. ^1^H-NMR (400 MHz, CDCl_3_) δ 7.22 (dd, *J* = 8.3, 2.1 Hz, 1H), 7.20–7.14 (m, 5H), 7.05 (d, *J* = 2.1 Hz, 1H), 6.76 (d, *J* = 8.3 Hz, 1H), 3.37 (s, 2H), 3.20 (s, 3H), 1.42 (s, 3H). ^13^C-NMR (100 MHz, CDCl_3_) δ 178.6, 142.1, 135.6, 133.9, 130.9, 128.8, 128.2, 128.0, 126.9, 124.1, 109.0, 49.7, 42.8, 26.5, 23.0. HRMS (ESI): calcd. for C_17_H_17_NOClS ([M + H]^+^) 318.0714, found 318.0716.

*3-((Benzo[d][1,3]dioxol-5-ylthio)methyl)-5-chloro-1,3-dimethylindolin-2-one* (**3g**). Yield 65 mg (91%), pale yellow solid. ^1^H-NMR (400 MHz, CDCl_3_) δ 7.19 (dd, *J* = 8.5, 2.0 Hz, 1H), 6.91 (d, *J* = 2.2 Hz, 1H), 6.79–6.72 (m, 1H), 6.66–6.52 (m, 3H), 5.92 (dd, *J* = 4.0, 2.4 Hz, 2H), 3.27 (d, *J* = 2.9 Hz, 2H), 3.21 (s, 3H), 1.37 (s, 3H). ^13^C-NMR (100 MHz, CDCl_3_) δ 178.7, 147.7, 147.5, 142.2, 133.9, 128.1, 127.9, 127.5, 126.5, 124.0, 112.9, 108.9, 108.9, 101.4, 50.1, 44.5, 26.5, 23.2. HRMS (ESI): calcd. for C_18_H_17_NO_3_ClS ([M + H]^+^) 362.0612, found 362.0605.

### 3.5. Procedure for the Preparation of 5-Chloro-1,3-dimethyl-3-((((trimethylsilyl)ethynyl)thio)methyl)indolin-2-one *(**3h**)*

To a solution of trimethylsilacetylene (60 mg, 0.6 mmol) in dry THF (2 mL) at 0 °C under N_2_ was added *n*-BuLi (0.4 mL of a 1.6 M solution in hexanes, 0.6 mmol) dropwise and the resulting reaction mixture was stirred for 0.5 h. A solution of **2g** (53 mg, 0.2 mmol) in THF (1 mL) was slowly added, via syringe, and the reaction was allowed to warm to room temperature, stirred for 2 h, and then quenched (saturated aq. NH_4_Cl), extracted (EtOAc, 2×) and dried (Na_2_SO_4_). Purification by flash chromatography (petroleum ether/EtOAc, 10:1) yielded the desired product **3h**. Yield 48 mg (71%), colorless oil.^1^H-NMR (400 MHz, CDCl_3_) δ 7.32 (d, *J* = 2.1 Hz, 1H), 7.28 (ddd, *J* = 8.3, 2.1, 0.5 Hz, 1H), 6.79 (d, *J* = 8.3 Hz, 1H), 3.24–3.10 (m, 5H), 1.46 (s, 3H), 0.11 (s, 9H). ^13^C-NMR (100 MHz, CDCl_3_) δ 177.9, 142.1, 133.3, 128.5, 128.0, 124.5, 109.2, 100.7, 93.7, 49.3, 43.3, 26.5, 22.6, −0.07. HRMS (ESI): calcd. for C_16_H_21_NOClSSi ([M + H]^+^) 338.0796, found 338.0791.

### 3.6. Procedure for the Preparation of 5-Chloro-3-((ethynylthio)methyl)-1,3-dimethylindolin-2-one *(**4**)*

To a solution of **2g** (53 mg, 0.2 mmol) and LiCl (24 mg, 0.6 mmol) in dry THF (3 mL) at 0 °C under N_2_ was added ethynylmagnesium chloride (1.2 mL of a 0.5 M solution in THF, 0.6 mmol), dropwise, via syringe. The reaction was allowed to warm to room temperature and stirred for 2 h, Upon completion of the reaction, saturated NH_4_Cl (aq) was added and extraction (EtOAc, 2 x). The crude product was dried (Na_2_SO_4_), filtered and evaporation. Flash chromatography over silica gel (petroleum ether/EtOAc, 10:1) then yielded the product *5-Chloro-3-((ethynylthio)methyl)-1,3-dimethylindolin-2-one* (**4**). Yield 32mg (65%), colorless oil. ^1^H-NMR (400 MHz, CDCl_3_) δ 7.31–7.29 (m, 1H), 7.29 (s, 1H), 6.82–6.78 (m, 1H), 3.22 (s, 3H), 3.19 (d, *J* = 3.4 Hz, 2H), 2.62 (s, 1H), 1.47 (s, 3H). ^13^C-NMR (100 MHz, CDCl_3_) δ 177.9, 142.2, 133.1, 128.6, 128.1, 124.5, 109.2, 82.0, 73.7, 49.3, 42.6, 26.6, 22.7. HRMS (ESI): calcd. for C_13_H_13_NOClS ([M + H]^+^) 266.0401, found 266.0396.

### 3.7. Procedure for the Preparation of Triazole 3-(((1-benzyl-1H-1,2,3-triazol-4-yl)thio)methyl)-5-chloro-1,3-dimethylindolin-2-one *(**5**)*

To a solution of **4** (26 mg, 0.1 mmol) in CH_2_Cl_2_/H_2_O (2:1, 2 mL) at room temperature was added CuSO_4_·5H_2_O (1.5 mg, 0.01 mmol) and sodium ascorbate (4 mg, 0.02 mmol). The reaction mixture was stirred for 1 h at room temperature and the solvents were removed under reduced pressure. Column chromatography of the residue (petroleum ether/EtOAc, 1:1) provided triazole *3-(((1-benzyl-1H-1,2,3-triazol-4-yl)thio)methyl)-5-chloro-1,3-dimethylindolin-2-one* (**5**). Yield 35 mg (91%), white solid. ^1^H-NMR (400 MHz, Methanol-*d*_4_) δ 7.40 (s, 1H), 7.39–7.31 (m, 3H), 7.30–7.23 (m, 3H), 7.03 (d, *J* = 2.1 Hz, 1H), 6.93 (d, *J* = 8.3 Hz, 1H), 5.55–5.35 (m, 2H), 3.53 (d, *J* = 13.8 Hz, 1H), 3.25 (d, *J* = 13.8 Hz, 1H), 3.15 (s, 3H), 1.33 (s, 3H). ^13^C-NMR (100 MHz, Methanol-*d*_4_) δ 179.1, 142.3, 139.0, 134.8, 133.8, 128.7, 128.3, 128.0, 127.9, 127.6, 126.6, 123.4, 109.6, 53.7, 50.1, 41.4, 25.3, 22.0. HRMS (ESI): calcd. for C_20_H_20_N_4_OClS ([M + H]^+^) 399.1046, found 399.1041.

### 3.8. Procedure for the Preparation of 3-(((1H-tetrazol-5-yl)thio)methyl)-5-chloro-1,3-dimethylindolin-2-one *(**6**)*

Compound **2g** (53 mg, 0.2 mmol), ZnBr_2_ (45 mg, 0.2 mmol, 1 equiv.) and NaN_3_ (32 mg, 0.5 mmol, 2.5 equiv.) were combined in a mixed solvent [H_2_O/*i*PrOH (1:1, 3 mL)] and refluxed for 1 h. Upon completion of the reaction, the mixture was diluted with EtOAc. The solvent was then removed under vacuo. Column chromatography of the residue (petroleum ether/EtOAc, 1:1) provided tetrazole **6**. Yield 55 mg (90%), white solid. ^1^H-NMR (400 MHz, Methanol-*d*_4_) δ 7.27–7.21 (m, 1H), 7.16 (d, *J* = 2.0 Hz, 1H), 6.95 (d, *J* = 8.3 Hz, 1H), 3.85 (d, *J* = 13.7 Hz, 1H), 3.63 (d, *J* = 13.7 Hz, 1H), 3.17 (s, 3H), 1.41 (s, 3H). ^13^C-NMR (126 MHz, Methanol-*d*_4_) δ 178.7, 154.4, 142.2, 132.9, 128.5, 128.1, 123.7, 109.8, 49.8, 39.3, 25.5, 21.7. HRMS (ESI): calcd. for C_12_H_13_N_5_OClS ([M + H]^+^) 310.0529, found 310.0531.

### 3.9. Procedure for the Preparation of S-((5-chloro-1,3-dimethyl-2-oxoindolin-3-yl)methyl) carbamothioate *(**7**)*

Compound **2g** (53 mg, 0.2 mmol) and 1mL 95% sulfuric acid was stirred for 2 h at room temperature. Upon completion of the reaction, the mixture was diluted with EtOAc and cooled water. The solvent was then removed under vacuo. The residue was purified by column chromatography on silica gel (petroleum/EtOAc, 1:1) to give the corresponding products **7**. Yield 40 mg (71%), white solid. ^1^H-NMR (400 MHz, Methanol-*d*_4_) δ 7.37 (d, *J* = 2.1 Hz, 1H), 7.31 (dd, *J* = 8.3, 2.2 Hz, 1H), 6.98 (d, *J* = 8.3 Hz, 1H), 3.48 (d, *J* = 13.6 Hz, 1H), 3.29 (d, *J* = 13.6 Hz, 1H), 3.22 (s, 3H), 1.42 (s, 3H).^13^C-NMR (100 MHz, Methanol-*d*_4_) δ 179.5, 168.0, 142.0, 134.1, 128.1, 127.9, 123.8, 109.4, 49.2, 35.4, 25.3, 21.5. HRMS (ESI): calcd. for C_12_H_13_N_2_OClSNa ([M + Na]^+^) 307.0284, found 307.0288.

### 3.10. Procedure for the Preparation of 5-chloro-1,3-dimethyl-3-(((trifluoromethyl)thio)methyl)indolin-2-one *(**8**)*

A mixture of **2g** (53 mg, 0.2 mmol) and CsF (30 mg, 0.2 mmol) was dissolved in MeCN (3 mL) and cooled to 0 °C. Then trifluoromethyltrimethylsilane (56.8 mg, 0.4 mmol) was added at once via syringe and the mixture was stirred at room temperature for 2 h. The resulting mixture was filtered through a short pad of celite and extracted with EtOAc. The resulting organic solution was washed with water. The organic layer was dried over Na_2_SO_4_, filtered and concentrated. The residue was purified by flash chromatography (petroleum ether/EtOAc, 5:1) to give the corresponding product **8**. Yield 55 mg (90%), colorless oil. ^1^H-NMR (400 MHz, CDCl_3_) δ 7.29 (dd, *J* = 8.3, 2.1 Hz, 1H), 7.22 (d, *J* = 2.0 Hz, 1H), 6.79 (d, *J* = 8.3 Hz, 1H), 3.29 (d, *J* = 0.5 Hz, 2H), 1.46 (s, 3H). ^13^C-NMR (100 MHz, CDCl_3_) δ 177.5, 141.8, 132.7, 131.9, 128.9, 128.3, 123.7, 109.4, 47.8, 36.2 (t, *J* = 2.1 Hz), 26.5, 22.6. ^19^F NMR (376 MHz, CDCl_3_) δ −41.1 (s). HRMS (ESI): calcd. for C_12_H_12_ONClF_3_S ([M + H]^+^) 310.0275, found 310.0276.

### 3.11. General Procedure for the Preparation of Compounds ***9a**–**b***

To a solution of **2g** (52 mg, 0.2 mmol) and H-P(O)(R^2^)_2_ (diphenylphosphine oxide or diethyl phosphite 0.3 mmol) in toluene (2 mL) at room temperature was added DBU (45 mg, 0.3 mmol). The reaction mixture was stirred for 3 h at room temperature and the solvents were removed under reduced pressure. Column chromatography of the residue (petroleum ether/EtOAc, 4:1) provided corresponding products **9a**–**b**.

*((5-Chloro-1,3-dimethyl-2-oxoindolin-3-yl)methyl) diphenylphosphinothioate* (**9a**). Yield 76 mg (87%), white solid. ^1^H-NMR (400 MHz, CDCl_3_) δ 7.82–7.75 (m, 2H), 7.73–7.66 (m, 2H), 7.53–7.39 (m, 6H), 7.23–7.19 (m, 2H), 6.74–6.69 (m, 1H), 3.18 (s, 3H), 1.39 (s, 3H). ^13^C-NMR (100 MHz, CDCl3) δ 177.8, 141.8, 133.3, 132.8, 132.7, 132.7, 132.7, 131.8, 131.7, 131.6, 131.6, 131.5, 128.9, 128.8, 128.8, 128.7, 128.6, 128.3, 124.2, 109.1, 48.5, 48.4, 35.6, 35.6, 23.0. HRMS (ESI): calcd. for C_23_H_22_NO_2_ClSP ([M + H]^+^) 442.0792, found 442.0784.

*((5-Chloro-1,3-dimethyl-2-oxoindolin-3-yl)methyl) O,O-diethyl phosphorothioate* (**9b**). Yield 57 mg (76%), colorless oil. ^1^H-NMR (400 MHz, CDCl_3_) δ 7.36–7.25 (m, 2H), 6.79 (d, *J* = 8.3 Hz, 1H), 4.17–3.87 (m, 4H), 3.30 (d, *J* = 11.2 Hz, 2H), 3.21 (d, *J* = 1.2 Hz, 3H), 1.44 (d, *J* = 1.4 Hz, 3H), 1.33 (td, *J* = 7.1, 0.9 Hz, 3H), 1.25 (td, *J* = 7.1, 0.9 Hz, 3H). ^13^C-NMR (100 MHz, CDCl_3_) δ 177.9, 142.1, 133.3, 128.6, 128.3, 124.2, 109.2, 63.8 (m), 48.9 (d, *J* = 6.7 Hz), 37.5 (d, *J* = 3.8 Hz), 16.0 (dd, *J* = 11.6, 7.4 Hz). HMS (ESI): calcd. for C_15_H_22_NO_4_ClSP ([M + H]^+^) 378.0683, found 378.0690.

### 3.12. Procedure for the Preparation of 3,3′-(disulfanediylbis(methylene))bis(5-chloro-1,3-dimethylindolin-2-one) *(**10**)*

To a solution of Et_2_NH (0.6 mmol) in dry THF (2 mL) at 0 °C under N_2_ was added *n*-BuLi (0.4 mL of a 1.6 M solution in hexanes, 0.6 mmol) dropwise and the resulting reaction mixture was stirred for 0.5 h. A solution of **2g** (53 mg, 0.2 mmol) in THF (1 mL) was slowly added, via syringe, and the reaction was allowed to warm to room temperature, stirred for 3 h, and then quenched (saturated aq. NH_4_Cl), extracted (EtOAc, 2×) and dried (Na_2_SO_4_). Purification by flash chromatography (petroleum ether/EtOAc, 3:1) yielded the desired product **10**.

*3,3′-(Disulfanediylbis(methylene))bis(5-chloro-1,3-dimethylindolin-2-one)(**10**)*. Yield 89 mg (83%), white solid. ^1^H-NMR (400 MHz, CDCl_3_) δ 7.30–7.22 (m, 3H), 7.19–7.17 (m, 1H), 7.14 (d, *J* = 2.1 Hz, 1H), 6.79 (d, *J* = 8.3 Hz, 1H), 6.76 (d, *J* = 8.2 Hz, 1H), 3.20 (d, *J* = 3.0 Hz, 6H), 3.13 (d, *J* = 13.4 Hz, 1H), 3.07–2.97 (m, 2H), 2.88 (d, *J* = 13.4 Hz, 1H), 1.35 (s, 6H). ^13^C-NMR (100 MHz, CDCl_3_) δ 178.3, 178.3, 142.2, 142.1, 133.7, 133.6, 128.4, 128.4, 128.0, 127.9, 124.1, 124.0, 109.2, 109.2, 49.4, 49.3, 48.7, 48.1, 26.5(4), 26.5(2), 23.1, 22.9. HRMS (ESI): calcd. for C_22_H_23_N_2_O_2_Cl_2_S_2_ ([M + H]^+^) 481.0578, found 481.0586.

## 4. Conclusions

In summary, we have developed an efficient arylthiocyanation of activated alkenes under mild conditions leading to biologically interesting 3-alkylthiocyanato-2-oxindoles, which was practical and straightforward to construct the C–C and C–SCN bonds in one pot, and was of broad functional group compatibility. Mechanistic studies suggested a unique NCS• radical addition path and clarified the dual roles of catalytic pyridine as base and crucial ligand to accelerate the oxidation of Ag(I) to Ag(II). Around the privileged 2-oxindole core, we demonstrate the versatility of the thiocyanate moiety in post-synthetic transformations for constructing new S-C(sp^3^/sp^2^/sp), S-P, and S-S bonds, which provides an easy access to many important bioisosteres in medicinal chemistry and an array of sulfur-containing 2-oxindoles that are difficult to prepare by other approaches. This protocol will likely open up new vistas to chemical biology community for exploiting the rich chemical potential of the SCN moiety. Further work to achieve effective thiocyanation with catalytic amount of silver are underway.

## Figures and Tables

**Table 1:**
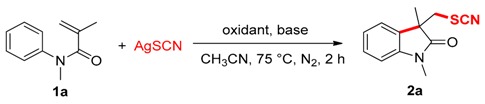
Optimization of the Reaction Conditions ^a^.

Entry	Oxidant	Base (equiv.)	Yield ^b^ (%)
1	DTBP	none	0
2	Oxone	none	0
3	PhI(OAc)	none	0
4	selectfluor	none	trace
5	CAN	none	15
6	K_2_S_2_O_8_	none	0
7	K_2_S_2_O_8_	NaHCO_3_ (1)	0
8	K_2_S_2_O_8_	Cs_2_CO_3_ (1)	trace
9	K_2_S_2_O_8_	HMPA (1)	0
10	K_2_S_2_O_8_	Et_3_N (1)	0
11	K_2_S_2_O_8_	DBU (1)	55
12	K_2_S_2_O_8_	pyridine (1)	83
13 ^c^	K_2_S_2_O_8_	Pyridine (0.2)	85
14 ^c^	K_2_S_2_O_8_	Pyridine (0.1)	64
15	none	Pyridine (0.2)	0

^a^*Reaction conditions*: **1a** (0.2 mmol), AgSCN (0.3 mmol), oxidant (1.5 equiv) and base in CH_3_CN (2.5 mL) at 75 °C for 2 h. ^b^ Isolated yields. ^c^ 8 h.

## Supplementary Materials

The following are available online, general methods, X-ray crystallography details for oxindole **2g**, mechanistic studies, and ^1^H-NMR and ^13^C-NMR spectra of all products.
