# Supplementary material for: Ag/Pyridine Co-Mediated Oxidative Arylthiocyanation of Activated Alkenes
Source: Molecules. 2018 Oct 22;23(10):2727. doi: 10.3390/molecules23102727 (PMC6222345; doi:10.3390/molecules23102727)

Supplementary Information

Article

Ag/Pyridine Co-Mediated Oxidative Arylthiocyanation of Activated Alkenes

De-Long Kong, Jian-Xun Du, Wei-Ming Chu, Chun-Ying Ma, Jia-Yi Tao and Wen-Hua Feng *

Department of New Drug Research and Development, Institute of Materia Medica, Peking Union Medical College & Chinese Academy of Medical Sciences, Beijing, 100050, China; kongdl@iccas.ac.cn (D.-L.K.); dujianxun@imm.ac.cn (J.-X.D.); chuweiming@imm.ac.cn (W.-M.C.); machunying@imm.ac.cn (C.-Y.M.); taojiayi@imm.ac.cn (J.-Y.T.)

* Correspondence: fwh@imm.ac.cn; Tel.: +86-010-8315-0067

1. X-ray crystallography of compound 2g (thermal ellipsoids are shown with 30% probability)

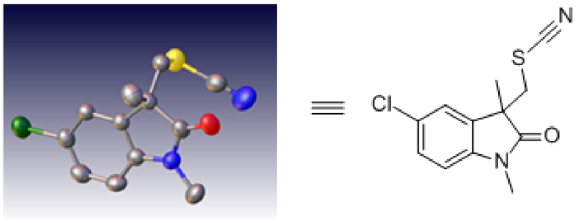

CCDC number: 1849159

Table S1. Crystal data and structure refinement for 2g

Identification code	**2g**
Empirical formula	$C_{12}H_{11}ClN_{2}Os$
Formula weight	266.74
Temperature/K	293(2)
Crystal system	Triclinic
Space group	P1
a/Å, b/Å, c/Å	7.1852(2), 24.5633(6), 7.5740(2)
α/°, β/°, γ/°	90, 108.676(3), 90
Volume/Å^3	1266.36(7)
Z	4
ρ_{calc}mg/mm^3	1.399
m/mm^{-1}	4.089
F(000)	552
Crystal size/mm^3	0.26 × 0.21 × 0.18
Theta range for data collection	3.599 to 66.663°

Index ranges	-7 ≤ h ≤ 8, -29 ≤ k ≤ 29, -8 ≤ l ≤ 8
Reflections collected	13972
Independent reflections	2231[R(int) = 0.0483]
Data/restraints/parameters	2231/0/157
Goodness-of-fit on F^2	1.055
Final R indexes [I>2σ (I)]	R_1 = 0.0406, wR_2 = 0.1017
Final R indexes [all data]	R_1 = 0.0500, wR_2 = 0.1098
Largest diff. peak/hole / e Å^{-3}	0.422/-0.284

Crystal Data. $C_{12}H_{11}ClN_2Os$, M =266.74, Triclinic, a = 7.1852(2) Å, b = 24.5633(6) Å, c = 7.5740(2) Å, α = 90°, β = 108.676(3), γ = 90°, U = 1266.36(7) Å^3, T = 293(2), space group P1 (no. 1), Z = 4, μ(Mo-Kα) = 4.089, 13972 reflections measured, 2231 unique (R_{int} = 0.0483) which were used in all calculations. The final $wR(F_2)$ was 0.1098 (all data).

2. Mechanistic Studies

In this section, clarify several mechanistic details are clarified: 1) this transformation proceeded through a unique NCS• radical addition path; 2) pyridine functioned not only as a base but also as a ligand to accelerate the oxidation of Ag(I) to Ag(II); 3) Ag(II) is the likely oxidant responsible for the formation of NCS• radical.

2.1. Radical Verification Experiments

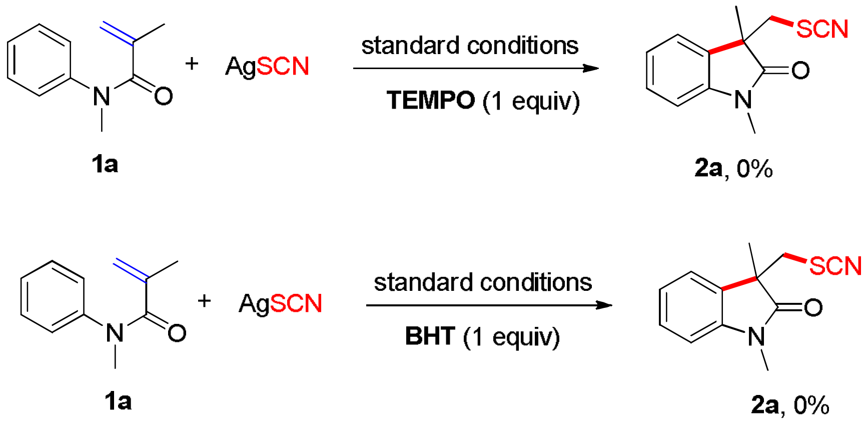

Compound **1a** (0.2 mmol), $K_2S_2O_8$ (81.0 mg, 0.3 mmol), AgSCN (48 mg, 0.3 mmol), TEMPO (31.3 mg, 0.2 mmol) or BHT (44 mg, 0.2 mmol) and were combined in an oven-dried sealed tube. The vessel was evacuated and backfilled with N_2 and CH_3CN (3 mL) and pyridine (3.5 μL, 0.04 mmol) were added via syringe. The tube was then sealed with a Teflon lined cap and placed into a preheated oil bath at 75 °C with vigorous stirring. After 8 h, the reaction mixture was cooled to room temperature and filtered through a plug of silica (eluted with EtOAc). Only trace **2a** was detected with 90% **1a** recovered. The result indicates that the radical intermediate probably be involved in the catalytic cycle of the reaction.

2.2 Role of Silver Experiments

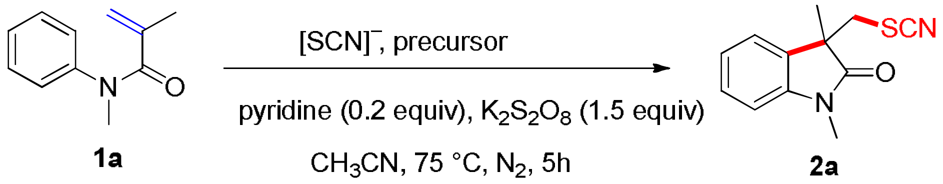

CuSCN (1.5 equiv)	**2a**, 0%
KSCN (1.5 equiv)	**2a**, 0%
KSCN/$AgNO_3$ (1:1, 1.5 equiv)	**2a**, 15%

2.3 Role of Pyridine Experiments

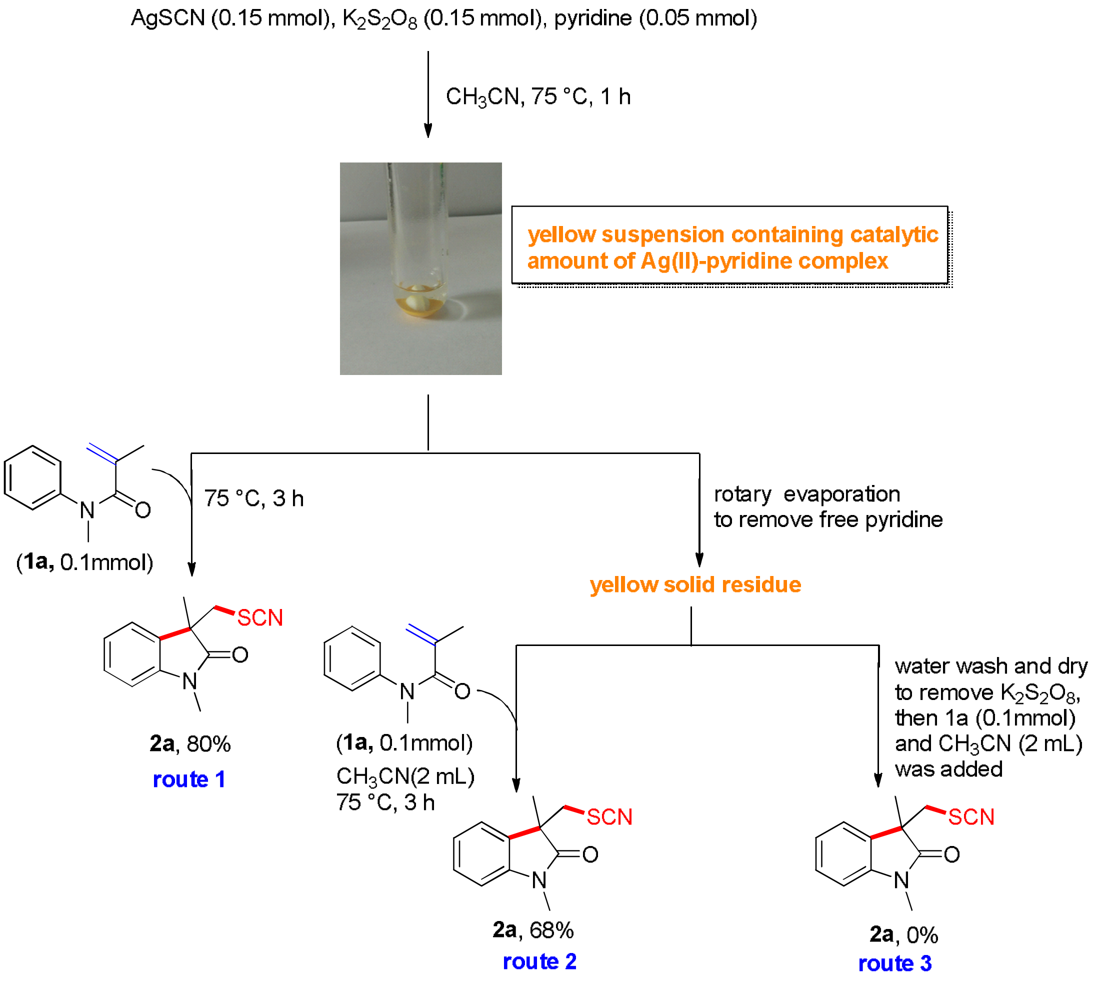

In the above experiment, we found that stirring of AgSCN/$K_2S_2O_8$/pyridine mixture in CH_3CN at 75 °C for 1 h led to the formation of colored Ag(II)–pyridine complexes, which could react with **1a** without free pyridine to give **2a** in 68% yield. However, the desired arylthiocyanation of **1a** with silver–pyridine complexes cannot proceed when $K_2S_2O_8$ was removed by water wash of yellow solid residue, which indicated the crucial roles of $K_2S_2O_8$ in oxidizing Ag(I) to Ag(II).

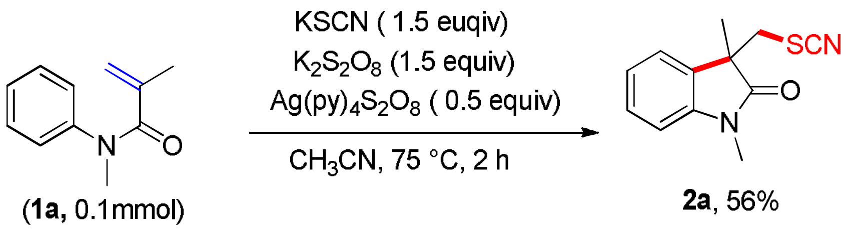

$Ag(py)_4S_2O_8$, which has been well established in the literature[2], was considered to be a possible formation of the Ag(II)–pyridine complex. Accordingly, it was prepared as an orange solid.[2a] Treatment of **1a** with $Ag(py)_4S_2O_8$ (0.5 equiv), KSCN(1.5 equiv), and $K_2S_2O_8$ (1.5 equiv) in CH_3CN at 75 °C for 2 h led to the formation of **2a** in 56% isolated yield. In this experiment, a very quick reduction of orange-Ag(II) to colorless-Ag(I) was observed at the beginning of the reaction which suggested that Ag(II) should be the active species to oxidize NCS^- to NCS• radical.

3. References

[1] Mu, X.; Wu, T.; Wang, H. Y.; Guo, Y. L.; Liu, G. *J. Am. Chem. Soc.* **2012**, *134*, 878.

[2] (a) H. Firouzabadi, P. Salehi, A. R. Sardarian and M. Seddighi, *Synthetic commun.*, 1991, **21**, 1121. (b) H. Firouzabadi, P. Salehi and I. Mohammadpour-Baltork, *Bull. Chem. Soc. Jpn.*, 1992, **65**, 2878. (c) A. Nikolaev, C. Y. Legault, M. Zhang and A. Orellana, *Org. Lett.*, 2018, **20**, 796

6. Copies of ^{1}H-NMR and ^{13}C-NMR spectra

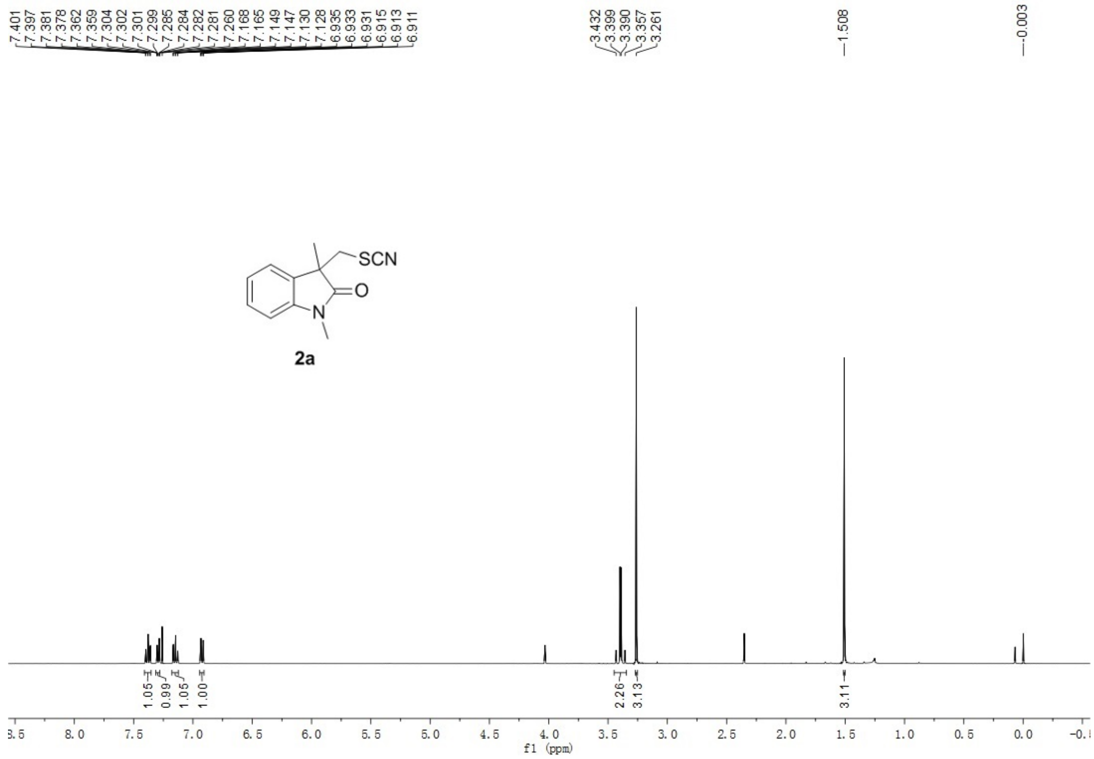

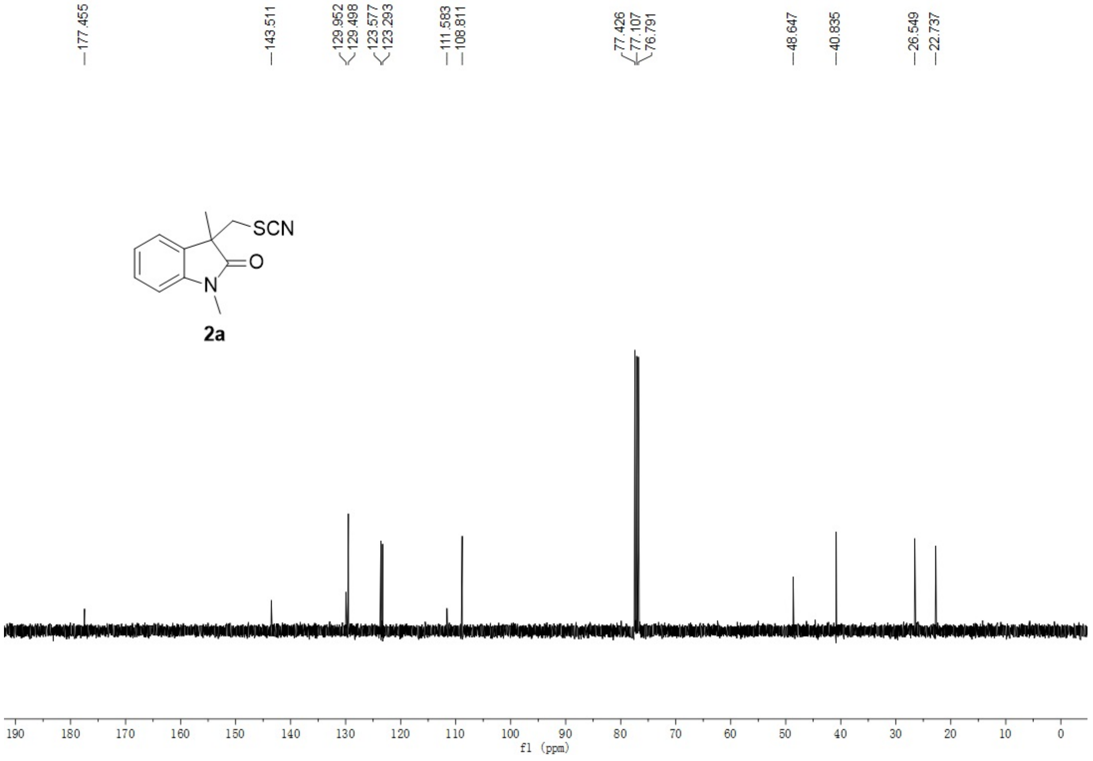

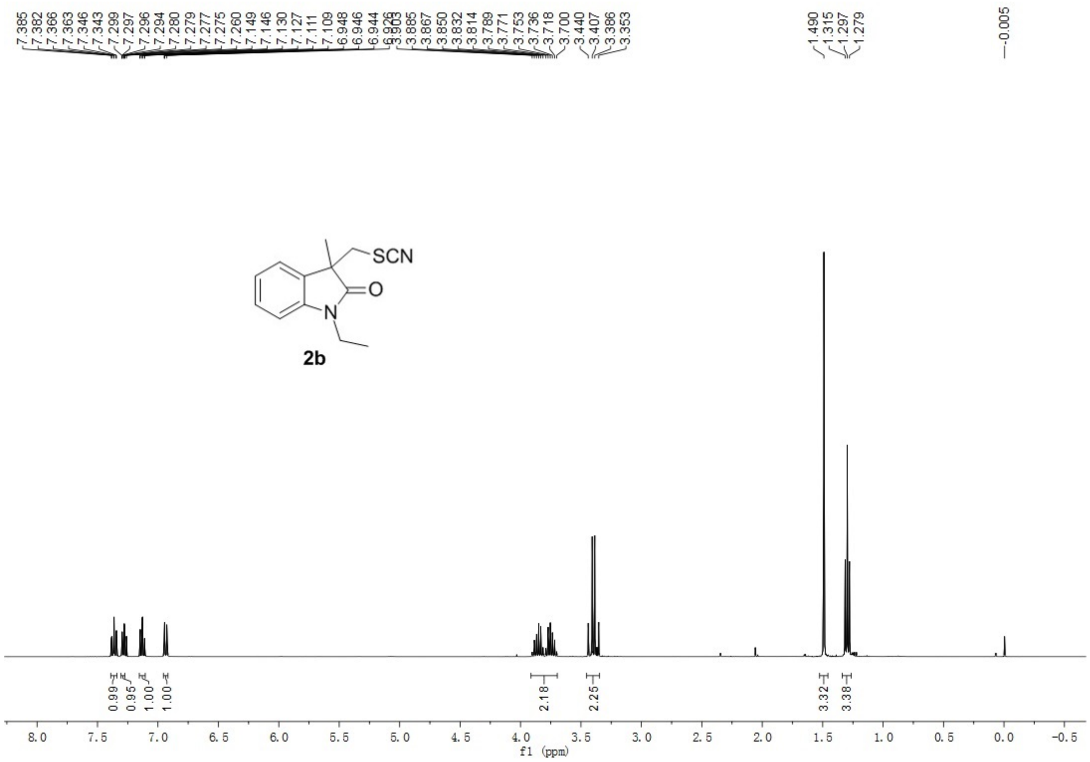
7.385
7.382
7.366
7.363
7.346
7.343
7.299
7.297
7.296
7.294
7.280
7.279
7.277
7.275
7.260
7.149
7.146
7.130
7.127
7.111
7.109
6.948
6.946
6.944
6.926
3.903
3.885
3.867
3.850
3.832
3.814
3.789
3.771
3.753
3.736
3.718
3.700
3.440
3.407
3.386
3.353
1.490
1.315
1.297
1.279
-0.005
SCN
O
N
2b
0.99
0.95
1.00
1.00
2.18
2.25
3.32
3.38
8.0 7.5 7.0 6.5 6.0 5.5 5.0 4.5 4.0 3.5 3.0 2.5 2.0 1.5 1.0 0.5 0.0 -0.5
f1 (ppm)

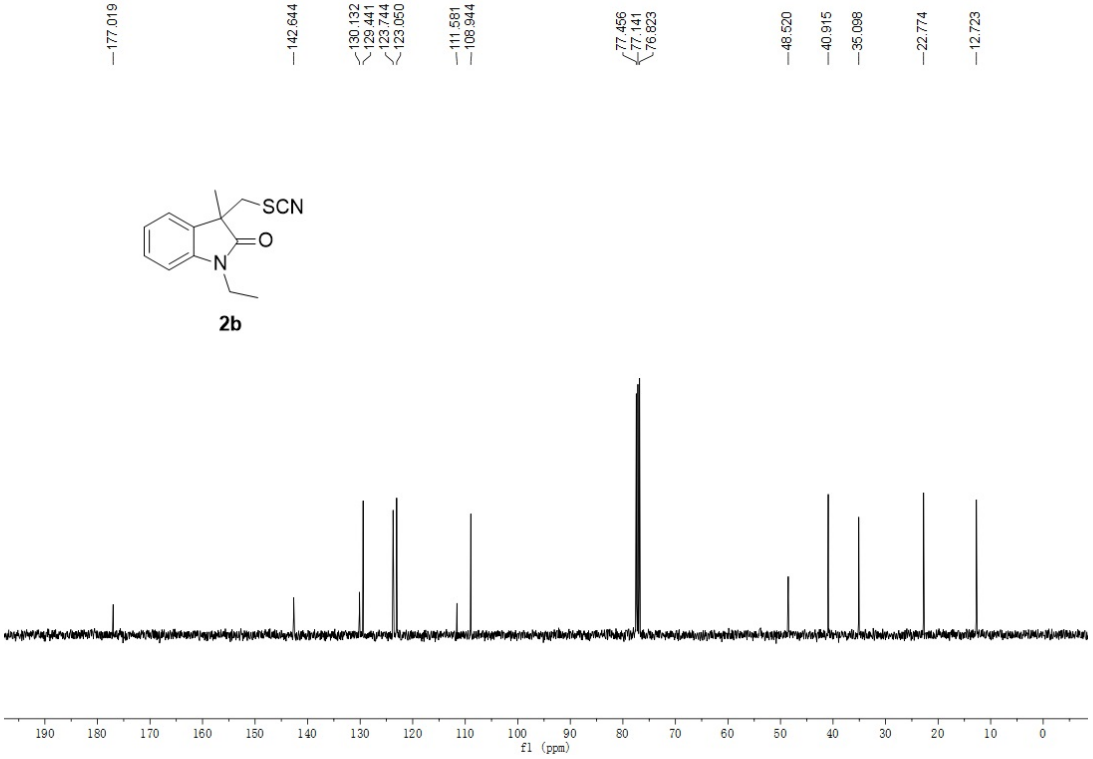
177.019
142.644
130.132
129.441
123.744
123.050
111.581
108.944
77.456
77.141
76.823
48.520
40.915
35.098
22.774
12.723
SCN
O
N
2b
190 180 170 160 150 140 130 120 110 100 90 80 70 60 50 40 30 20 10 0
f1 (ppm)

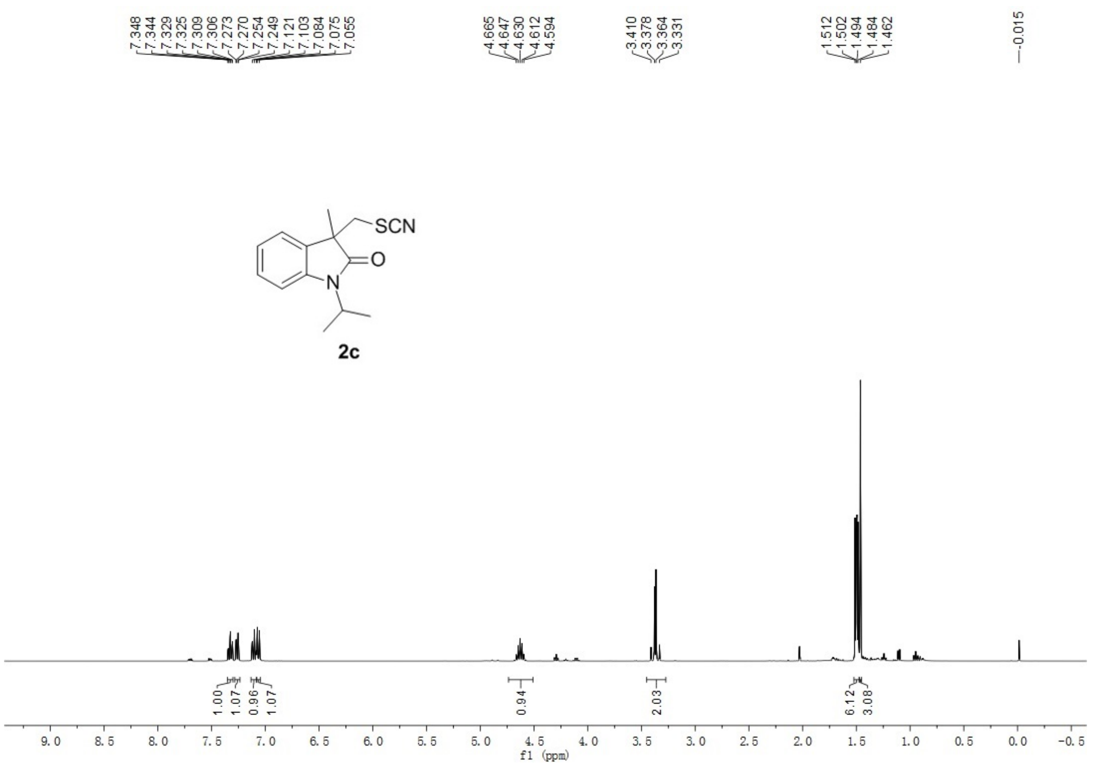

2c

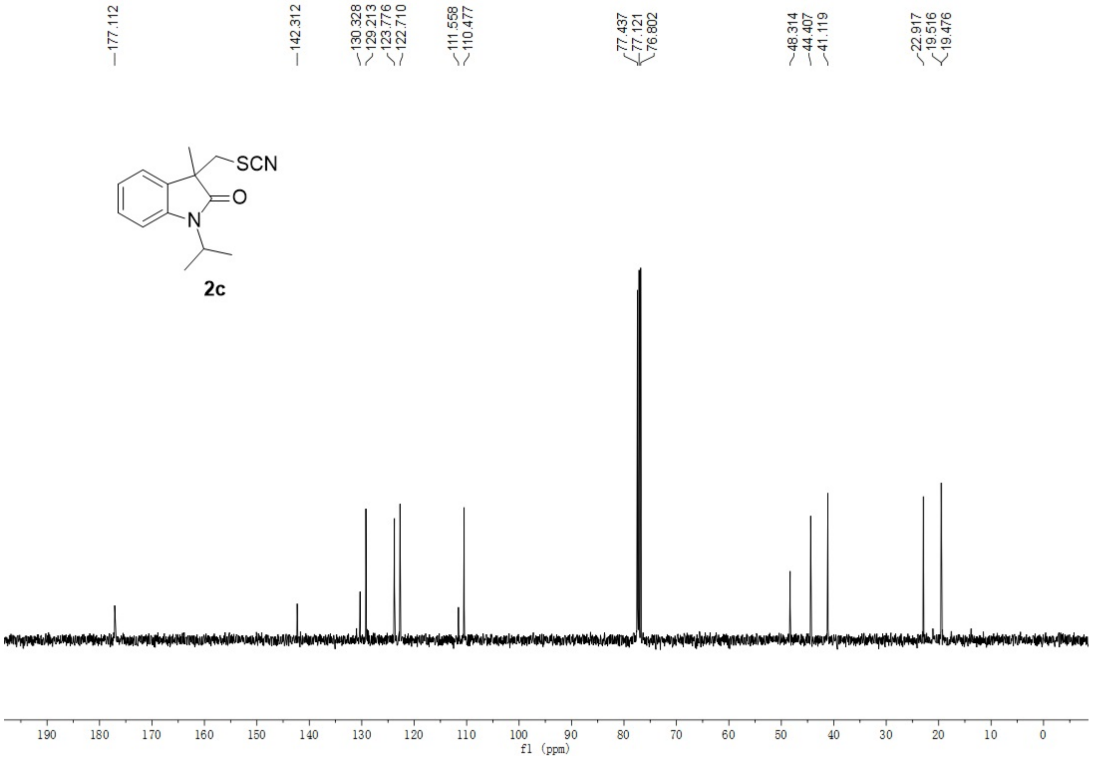

2c

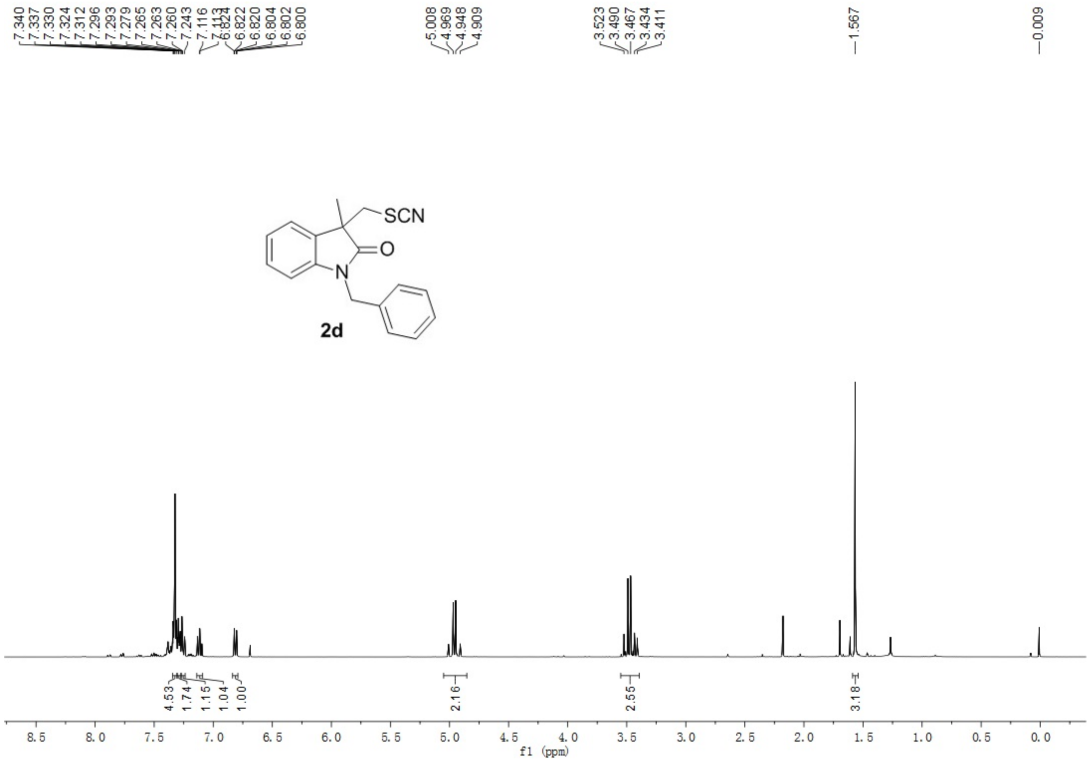
7.340
7.337
7.330
7.324
7.312
7.296
7.293
7.279
7.265
7.263
7.260
7.243
7.116
7.113
6.824
6.822
6.820
6.804
6.802
6.800
5.008
4.969
4.948
4.909
3.523
3.490
3.467
3.434
3.411
1.567
0.009
SCN
O
N
2d
4.53
1.74
1.15
1.04
1.00
2.16
2.55
3.18
f1 (ppm)

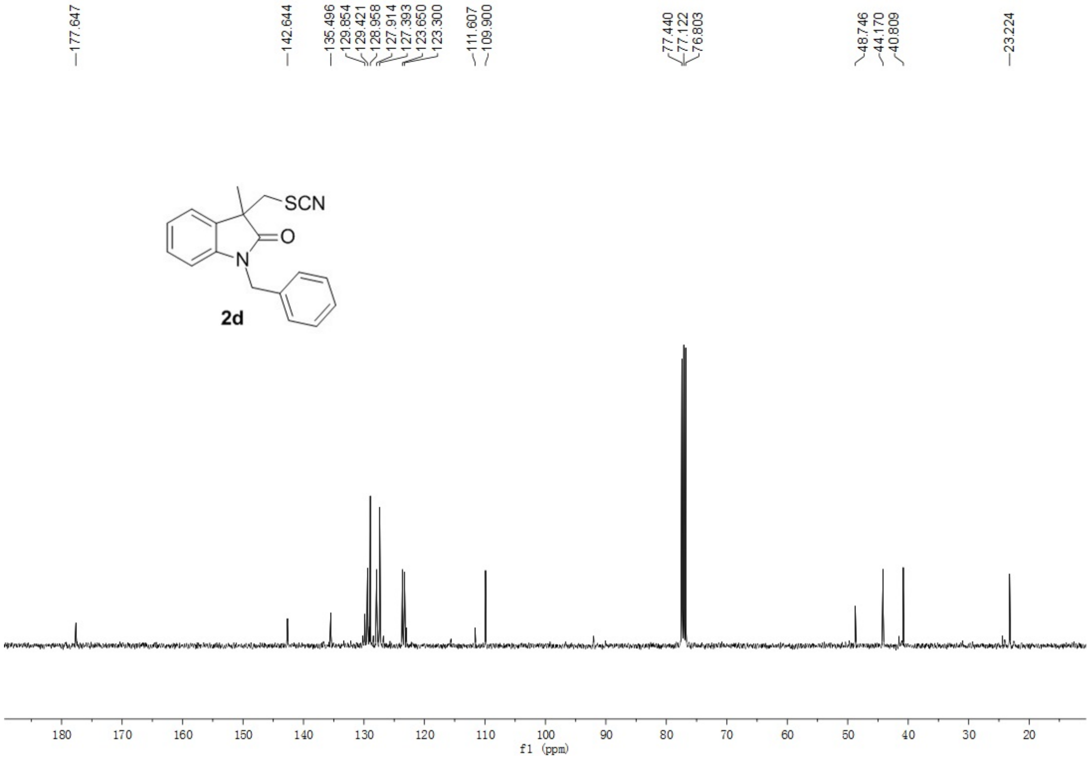
177.647
142.644
135.496
129.854
129.421
128.958
127.914
127.393
123.650
123.300
111.607
109.900
77.440
77.122
76.803
48.746
44.170
40.809
23.224
SCN
O
N
2d
f1 (ppm)

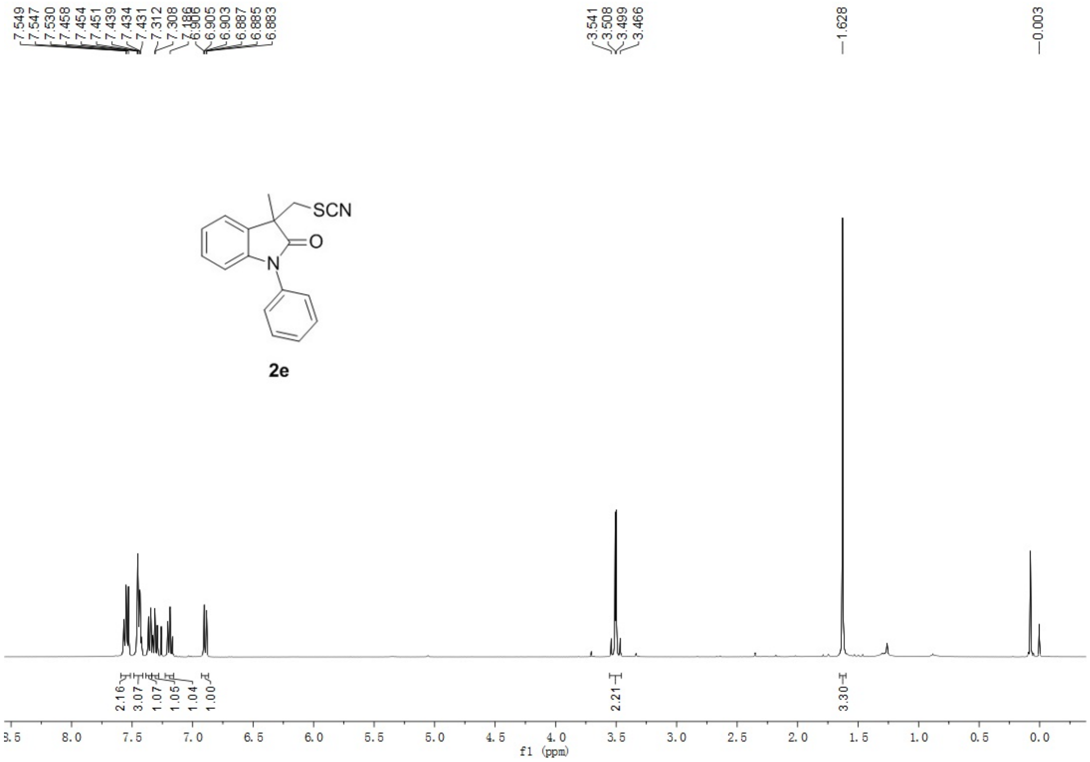

7.549
7.547
7.530
7.458
7.454
7.451
7.439
7.434
7.431
7.312
7.308
6.905
6.903
6.887
6.885
6.883
3.541
3.508
3.499
3.466
1.628
0.003
SCN
O
N
2e
2.16
3.07
1.07
1.05
1.04
1.00
2.21
3.30
f1 (ppm)

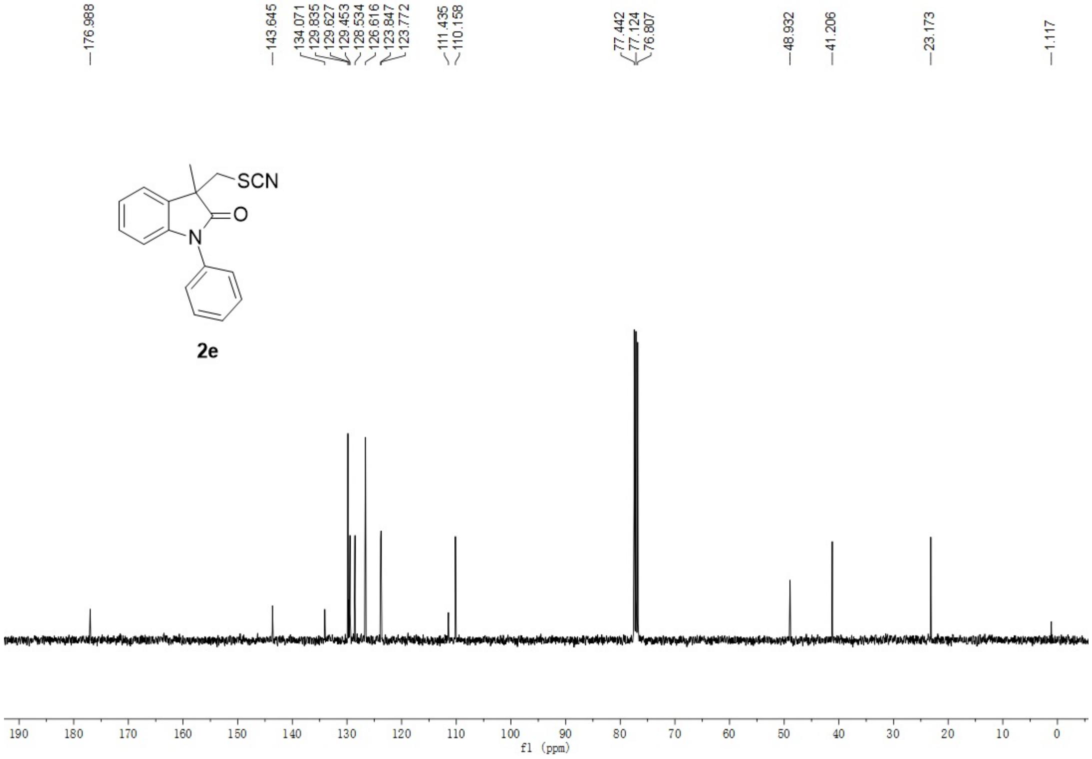

176.988
143.645
134.071
129.835
129.627
129.453
128.534
126.616
123.847
123.772
111.435
110.158
77.442
77.124
76.807
48.932
41.206
23.173
1.117
SCN
O
N
2e
f1 (ppm)

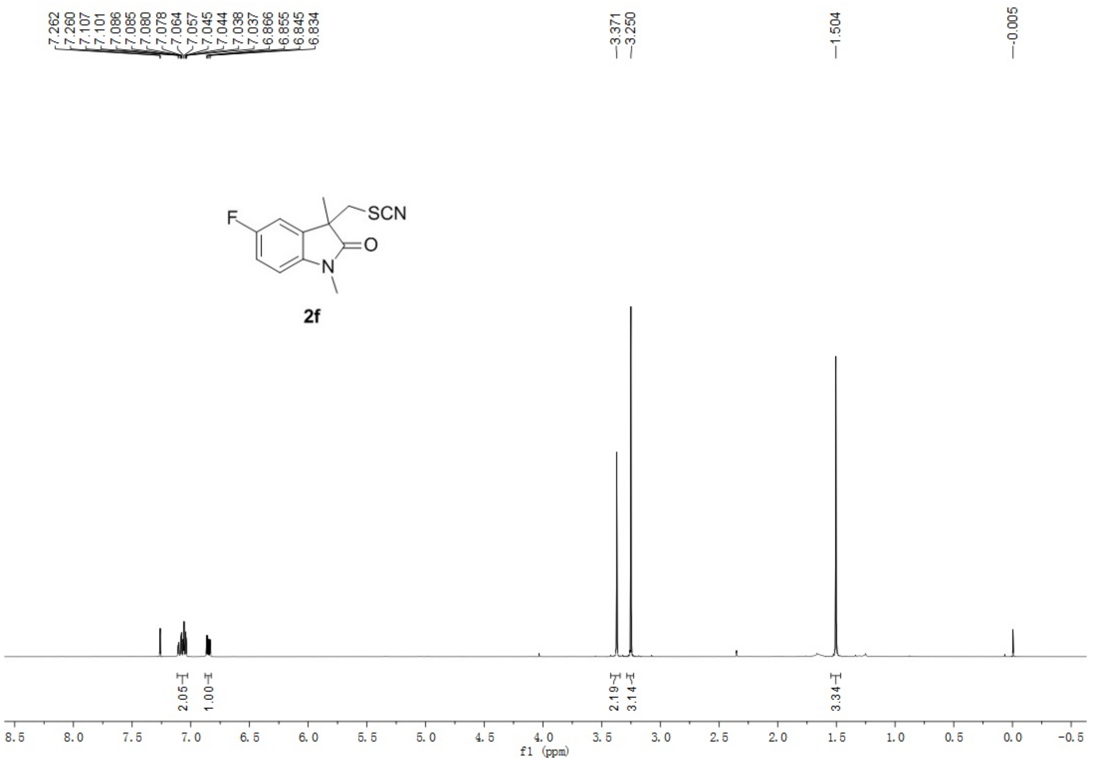

7.262
7.260
7.107
7.101
7.086
7.085
7.080
7.078
7.064
7.057
7.045
7.044
7.038
7.037
6.866
6.855
6.845
6.834
3.371
3.250
1.504
-0.005
F
SCN
O
N
2f
2.05
1.00
2.19
3.14
3.34
f1 (ppm)

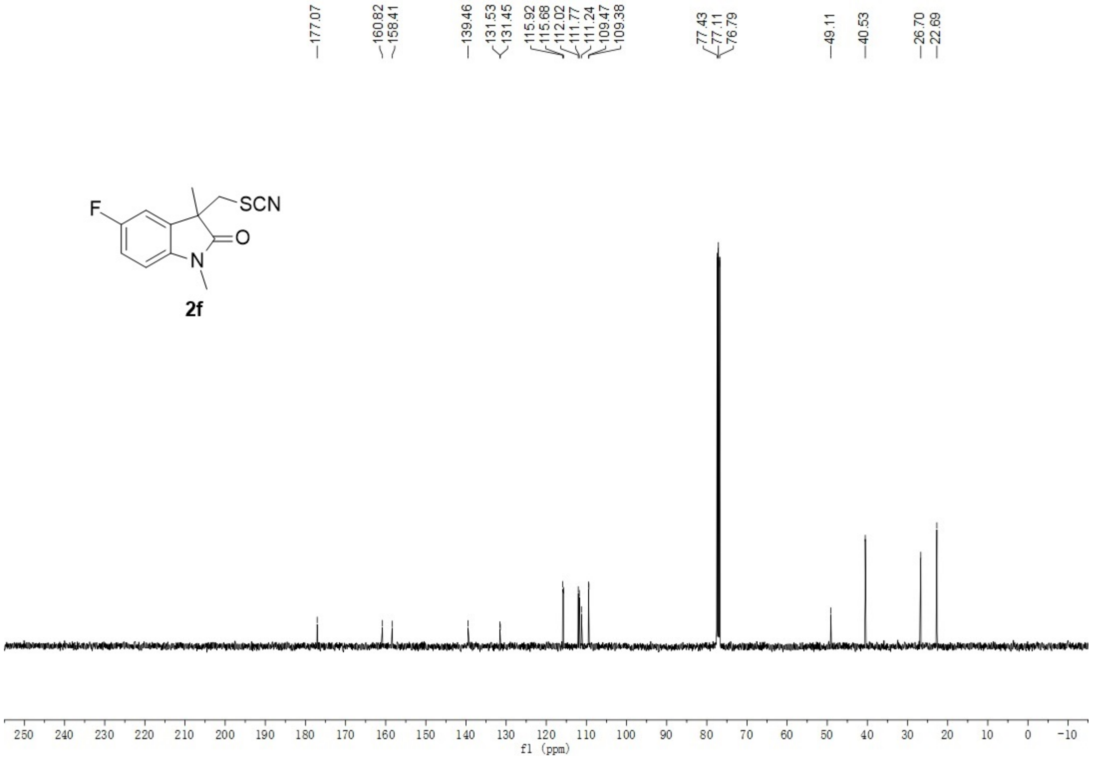

177.07
160.82
158.41
139.46
131.53
131.45
115.92
115.68
112.02
111.77
111.24
109.47
109.38
77.43
77.11
76.79
49.11
40.53
26.70
22.69
F
SCN
O
N
2f
f1 (ppm)

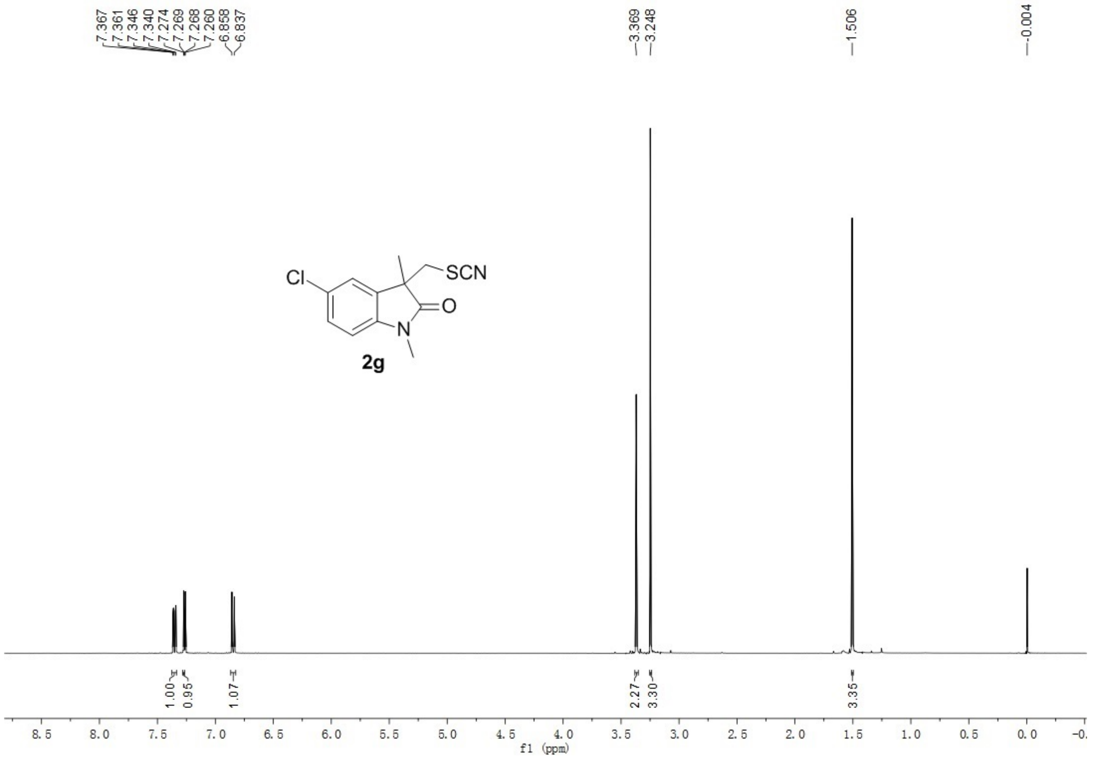

7.367
7.361
7.346
7.340
7.274
7.269
7.268
7.260
6.858
6.837
3.369
3.248
1.506
-0.004
SCN
Cl
O
N
2g
1.00
0.95
1.07
2.27
3.30
3.35
f1 (ppm)

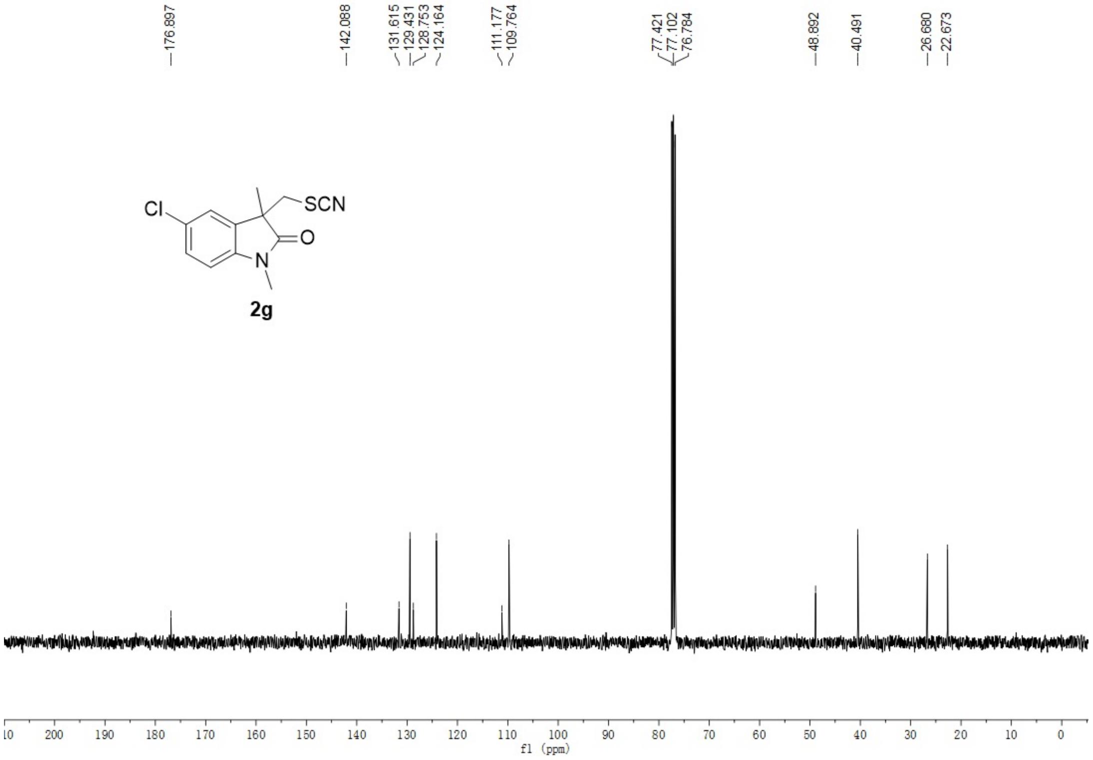

176.897
142.088
131.615
129.431
128.753
124.164
111.177
109.764
77.421
77.102
76.784
48.892
40.491
26.680
22.673
SCN
Cl
O
N
2g
f1 (ppm)

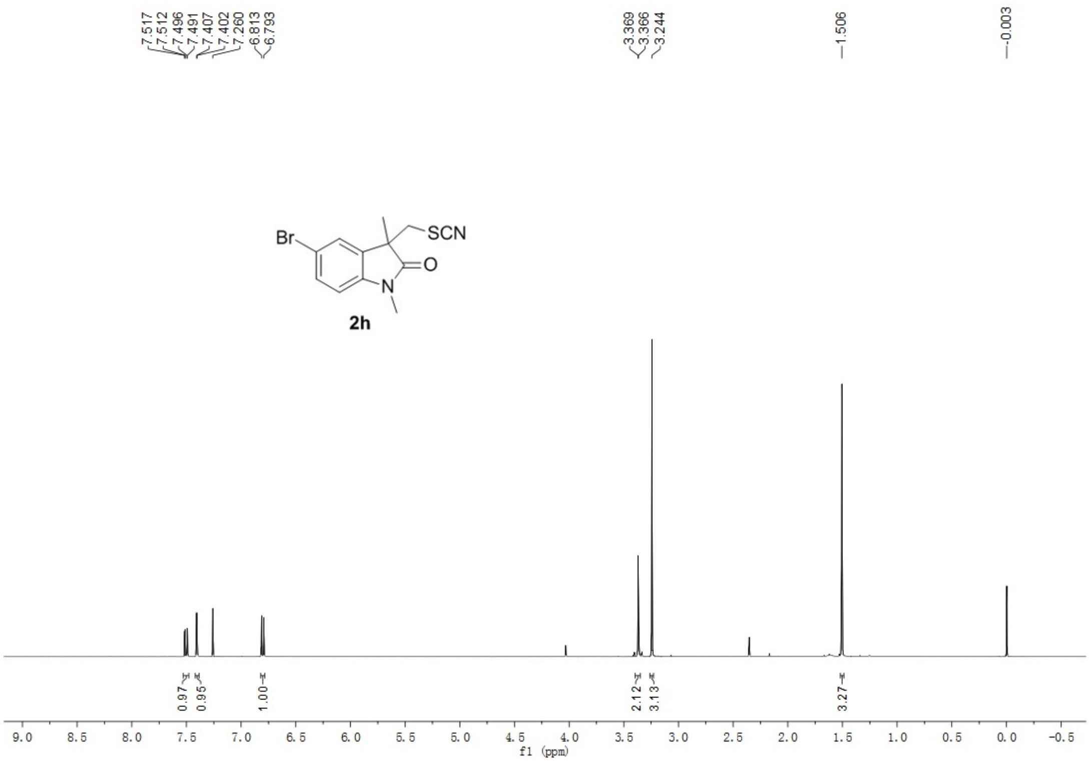

7.517
7.512
7.496
7.491
7.407
7.402
7.260
6.813
6.793
3.369
3.366
3.244
1.506
-0.003
Br
SCN
O
N
2h
0.97
0.95
1.00
2.12
3.13
3.27
9.0 8.5 8.0 7.5 7.0 6.5 6.0 5.5 5.0 4.5 4.0 3.5 3.0 2.5 2.0 1.5 1.0 0.5 0.0 -0.5
f1 (ppm)

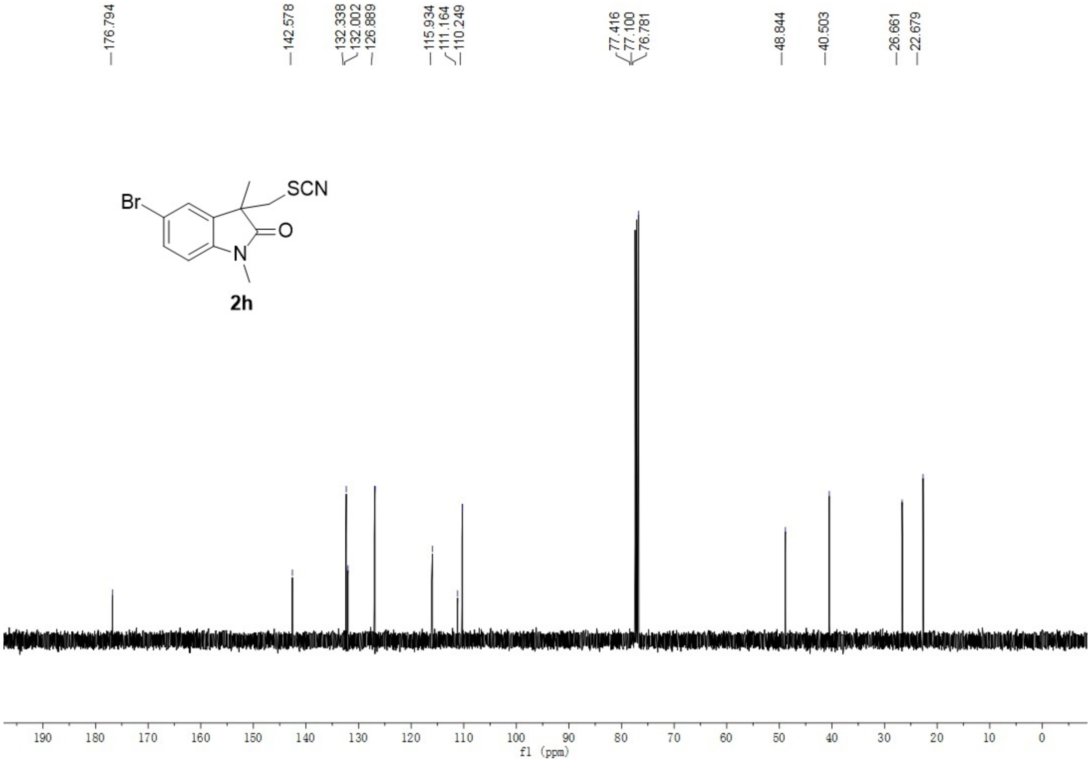

176.794
142.578
132.338
132.002
126.889
115.934
111.164
110.249
77.416
77.100
76.781
48.844
40.503
26.661
22.679
Br
SCN
O
N
2h
190 180 170 160 150 140 130 120 110 100 90 80 70 60 50 40 30 20 10 0
f1 (ppm)

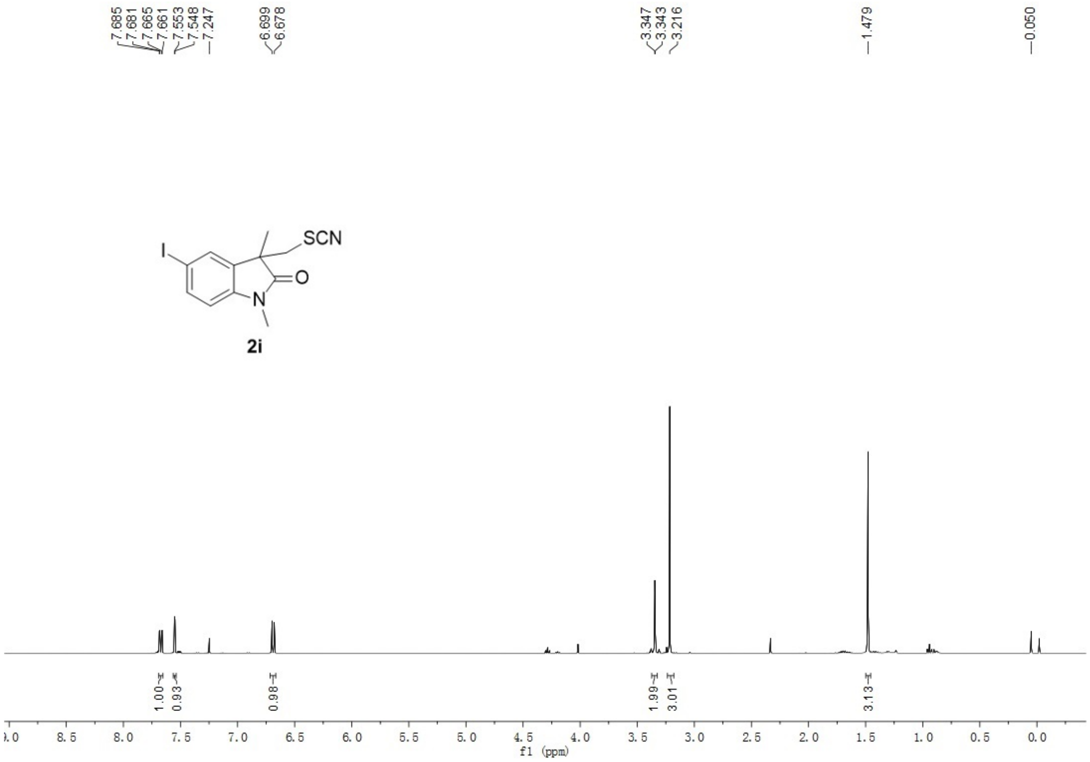
7.685
7.681
7.665
7.661
7.553
7.548
7.247
6.699
6.678
3.347
3.343
3.216
1.479
-0.050
SCN
I
O
N
2i
1.00
0.93
0.98
1.99
3.01
3.13
f1 (ppm)

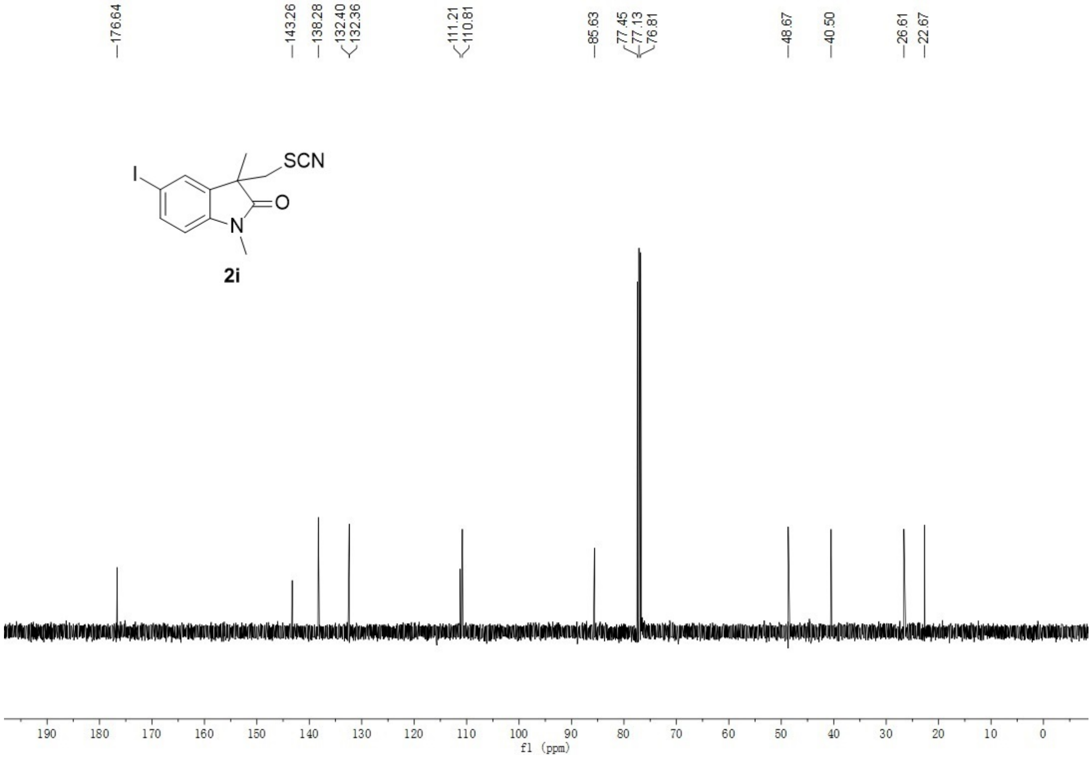
176.64
143.26
138.28
132.40
132.36
111.21
110.81
85.63
77.45
77.13
76.81
48.67
40.50
26.61
22.67
SCN
I
O
N
2i
f1 (ppm)

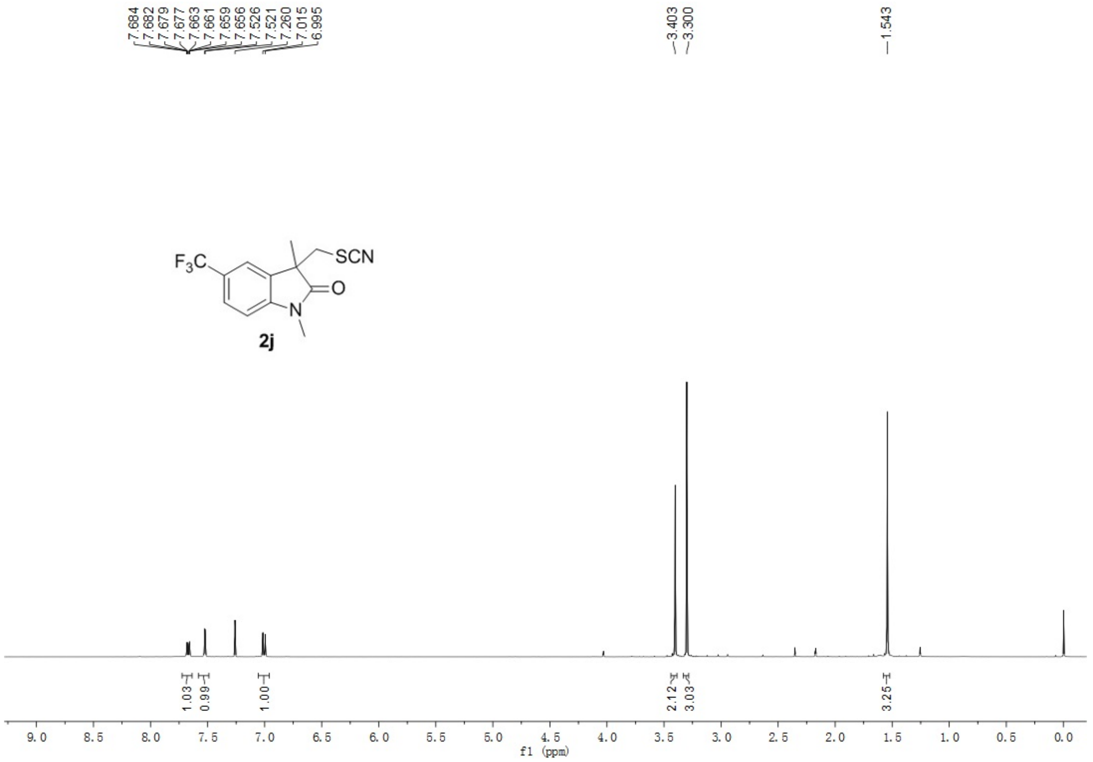
7.684
7.682
7.679
7.677
7.663
7.661
7.659
7.656
7.526
7.521
7.260
7.015
6.995
3.403
3.300
1.543
F_3C
SCN
O
N
2j
1.03
0.99
1.00
2.12
3.03
3.25
f1 (ppm)

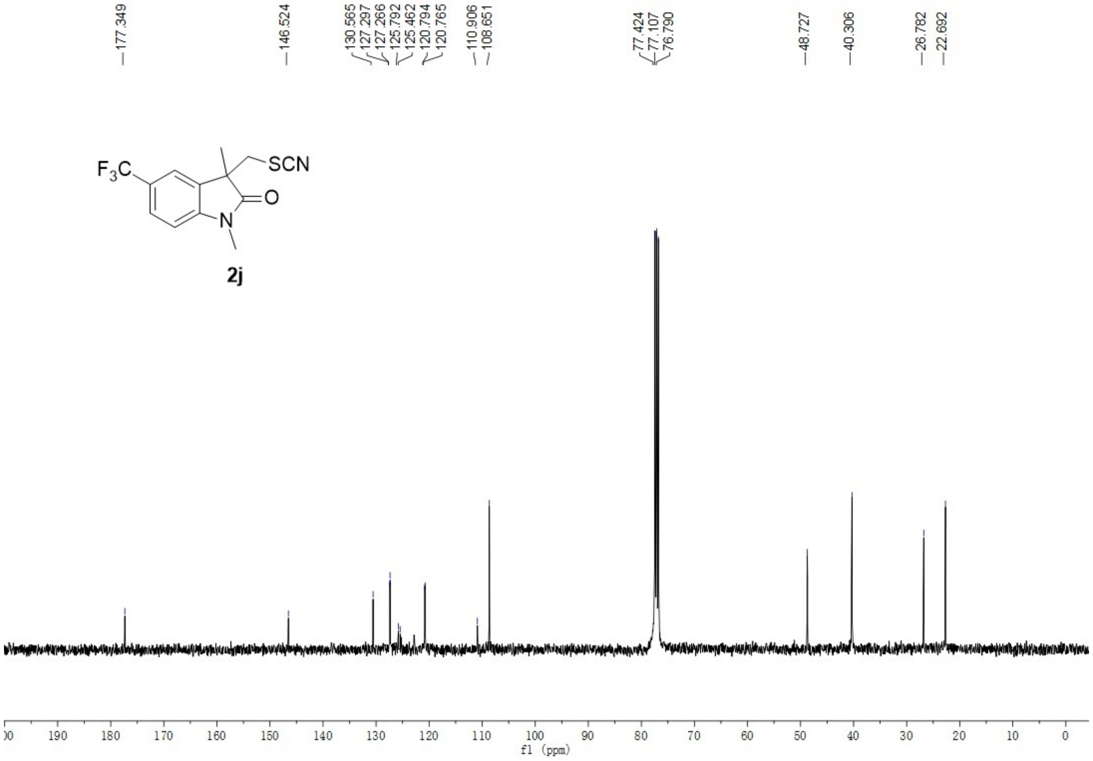
177.349
146.524
130.565
127.297
127.266
125.792
125.462
120.794
120.765
110.906
108.651
77.424
77.107
76.790
48.727
40.306
26.782
22.692
F_3C
SCN
O
N
2j
f1 (ppm)

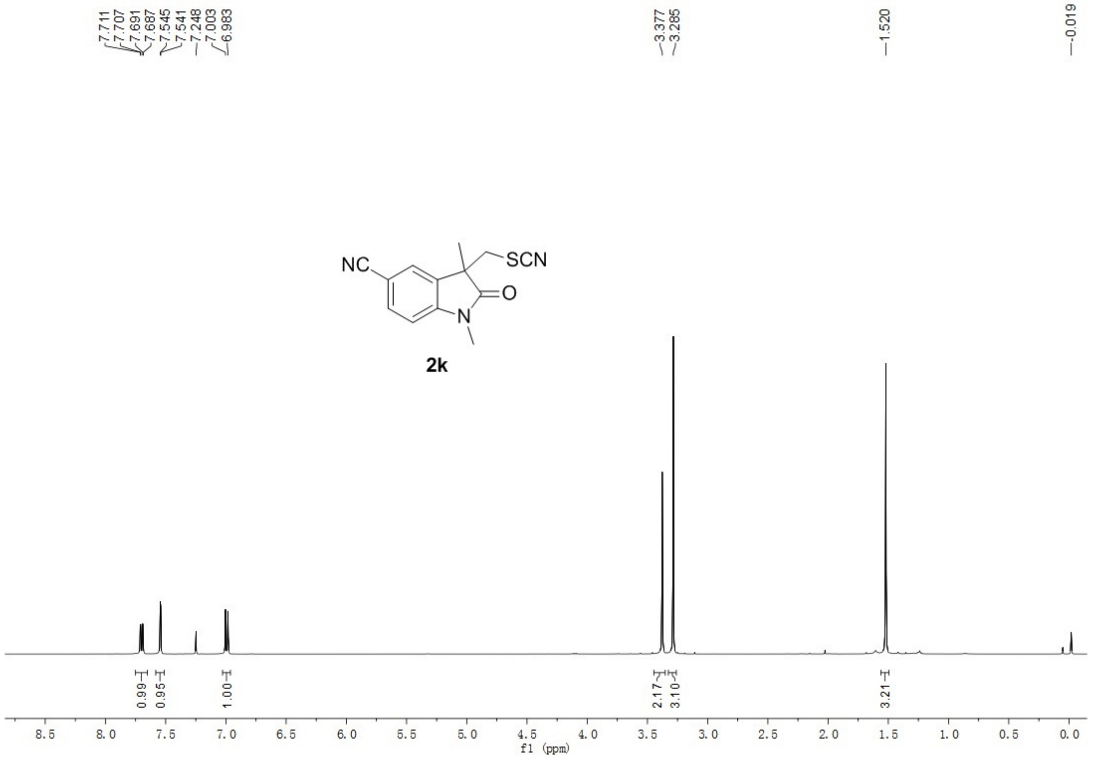

2k

7.711
7.707
7.691
7.687
7.545
7.541
7.248
7.003
6.983
3.377
3.285
1.520
-0.019
0.99
0.95
1.00
2.17
3.10
3.21
f1 (ppm)

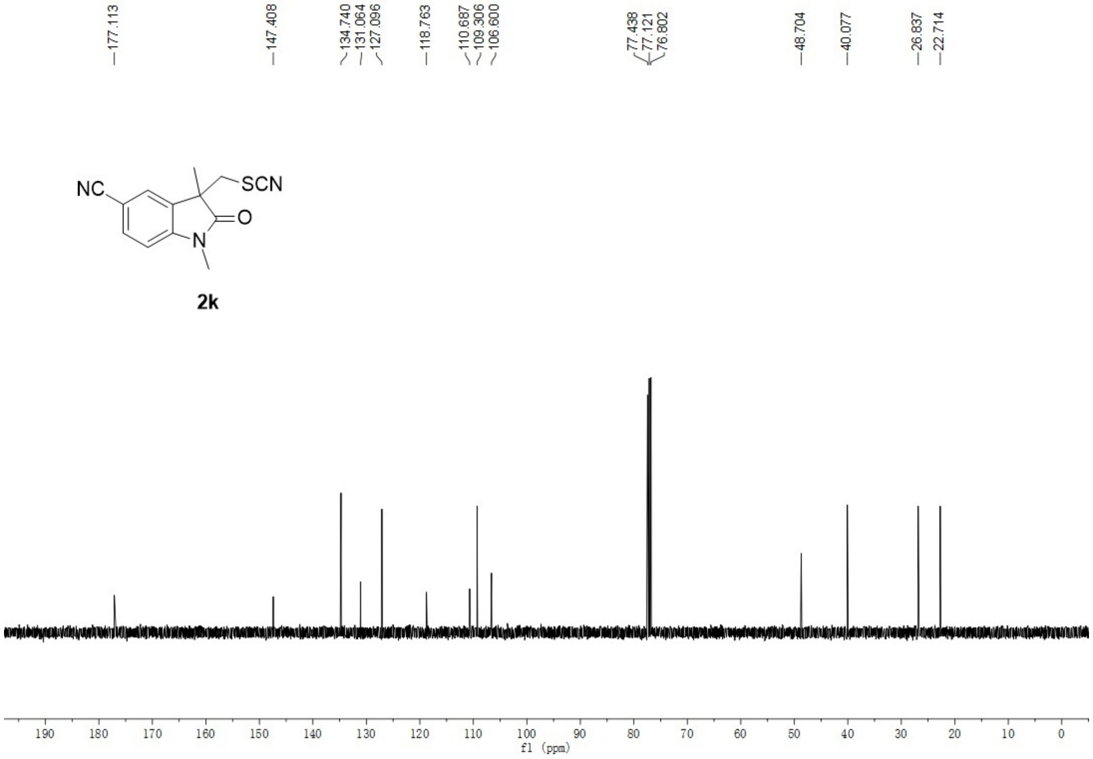

2k

177.113
147.408
134.740
131.064
127.096
118.763
110.687
109.306
106.600
77.438
77.121
76.802
48.704
40.077
26.837
22.714
f1 (ppm)

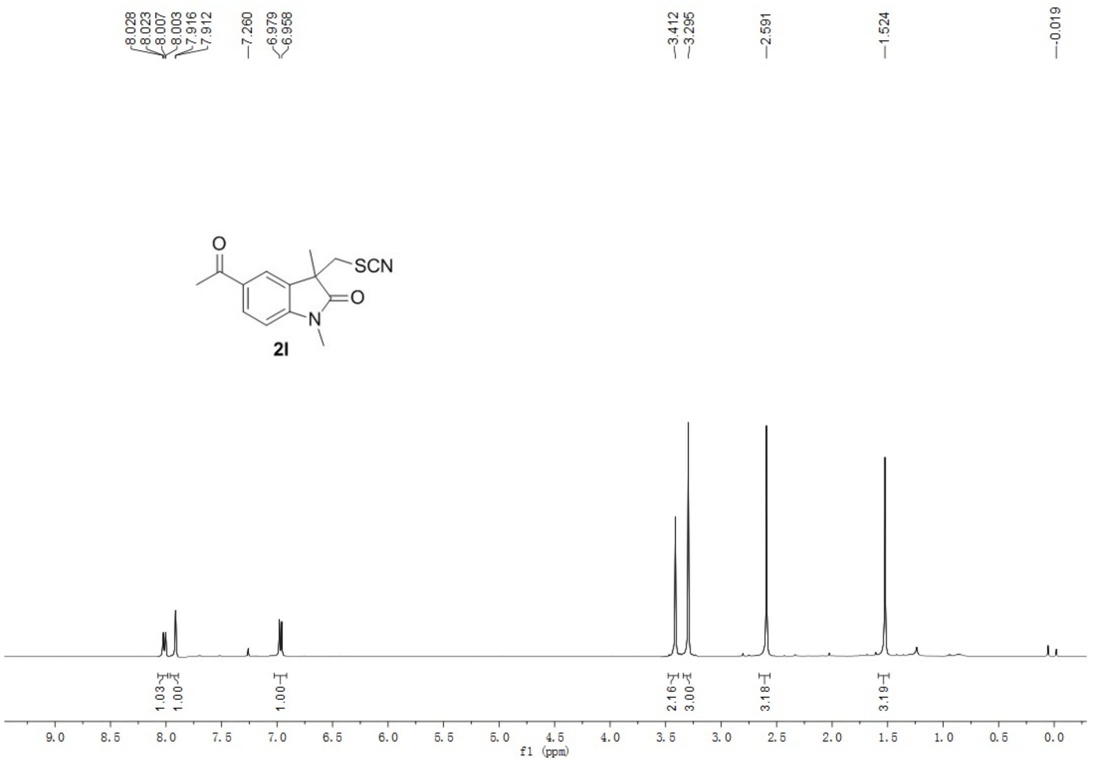
8.028
8.023
8.007
8.003
7.916
7.912
7.260
6.979
6.958
3.412
3.295
2.591
1.524
-0.019
SCN
O
N
2l
1.03
1.00
1.00
2.16
3.00
3.18
3.19
f1 (ppm)

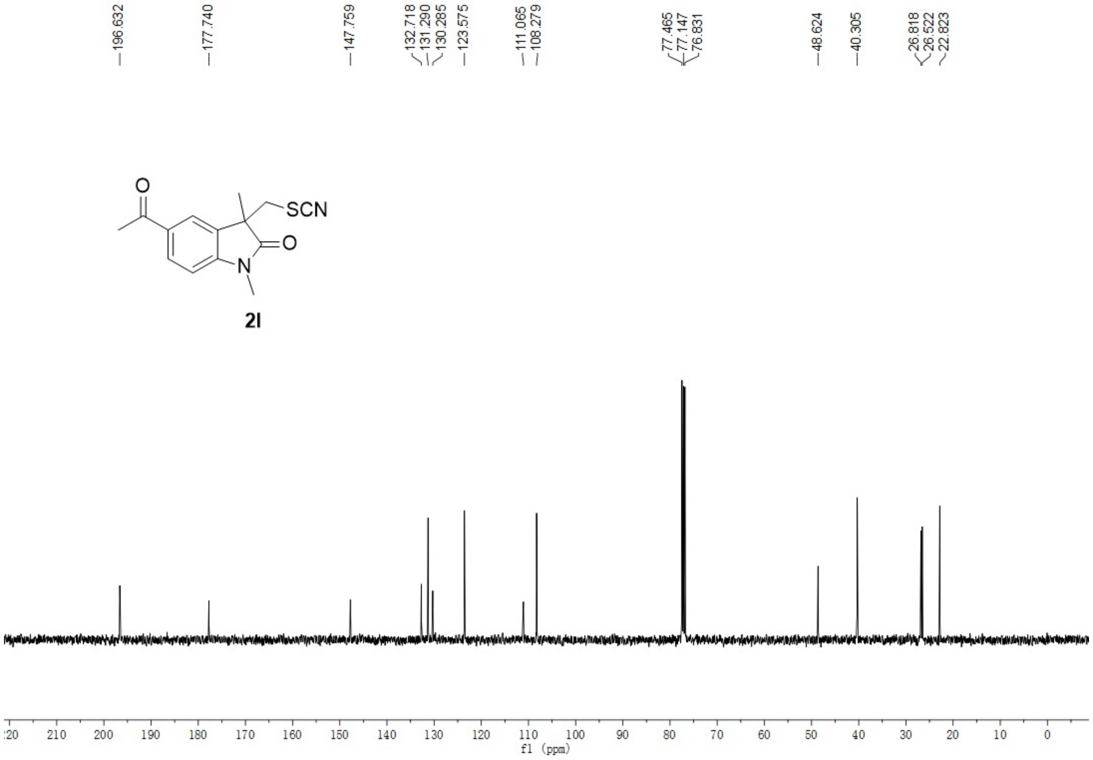
196.632
177.740
147.759
132.718
131.290
130.285
123.575
111.065
108.279
77.465
77.147
76.831
48.624
40.305
26.818
26.522
22.823
SCN
O
N
2l
f1 (ppm)

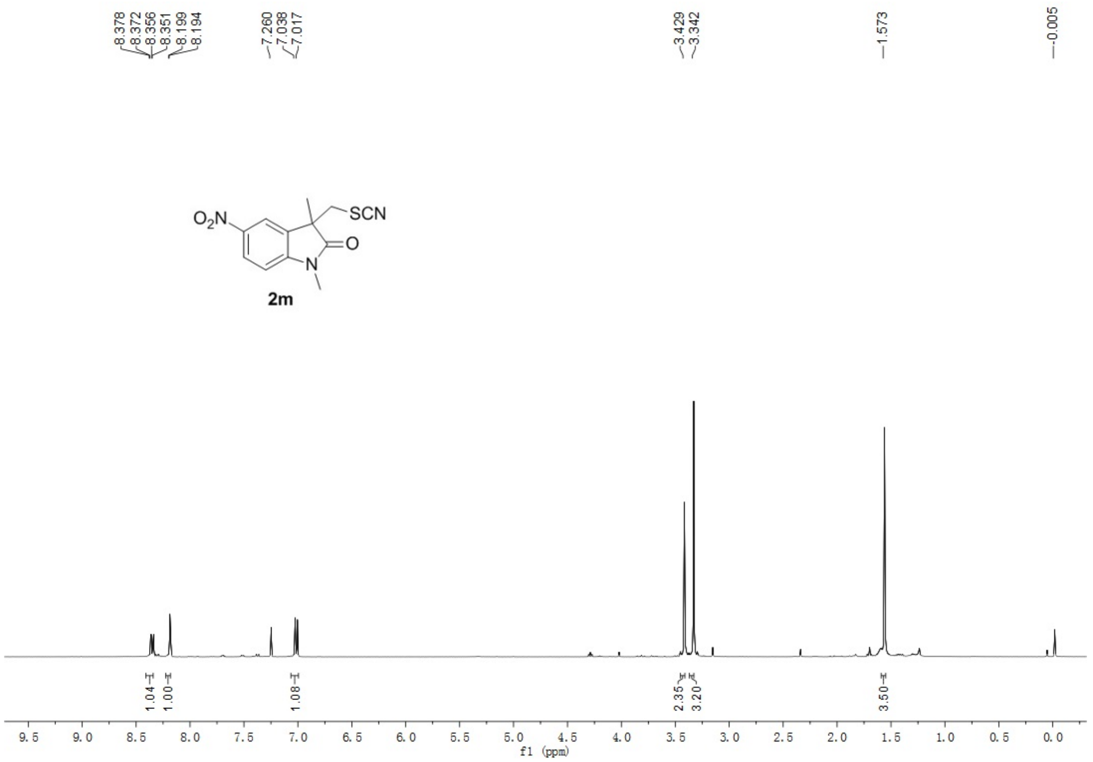

8.378
8.372
8.356
8.351
8.199
8.194
7.260
7.038
7.017
3.429
3.342
1.573
-0.005
O2N
SCN
O
N
2m
1.04
1.00
1.08
2.35
3.20
3.50
9.5 9.0 8.5 8.0 7.5 7.0 6.5 6.0 5.5 5.0 4.5 4.0 3.5 3.0 2.5 2.0 1.5 1.0 0.5 0.0
f1 (ppm)

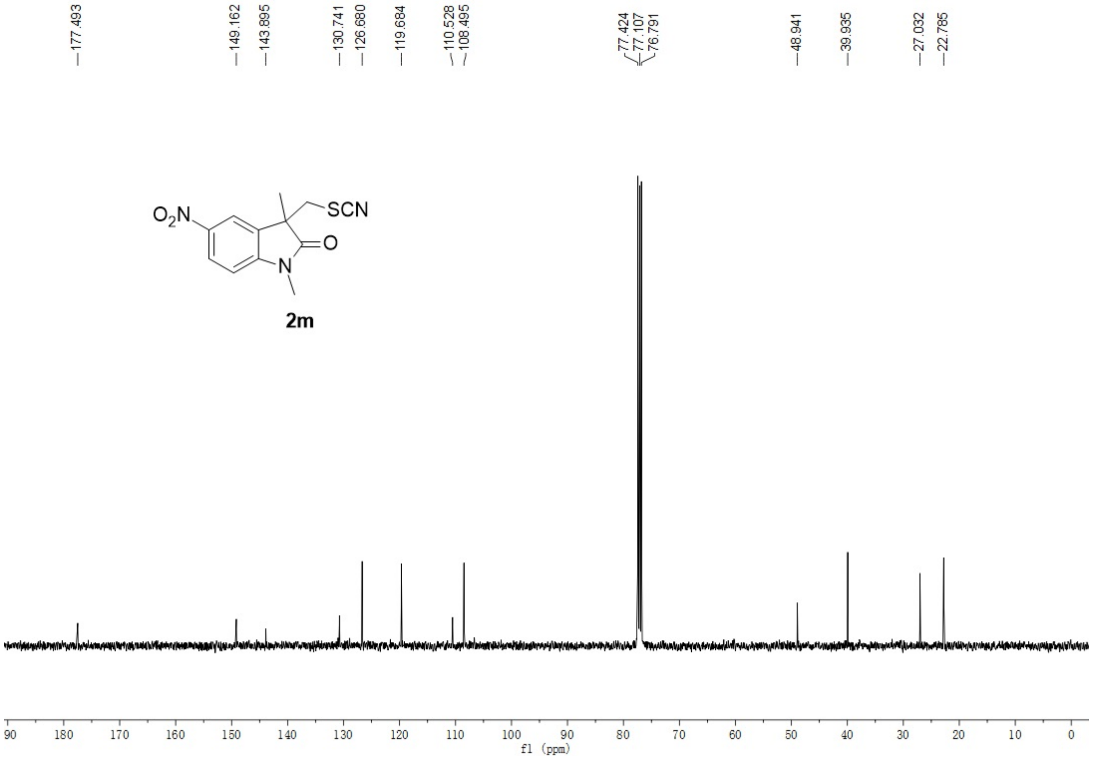

177.493
149.162
143.895
130.741
126.680
119.684
110.528
108.495
77.424
77.107
76.791
48.941
39.935
27.032
22.785
O2N
SCN
O
N
2m
90 180 170 160 150 140 130 120 110 100 90 80 70 60 50 40 30 20 10 0
f1 (ppm)

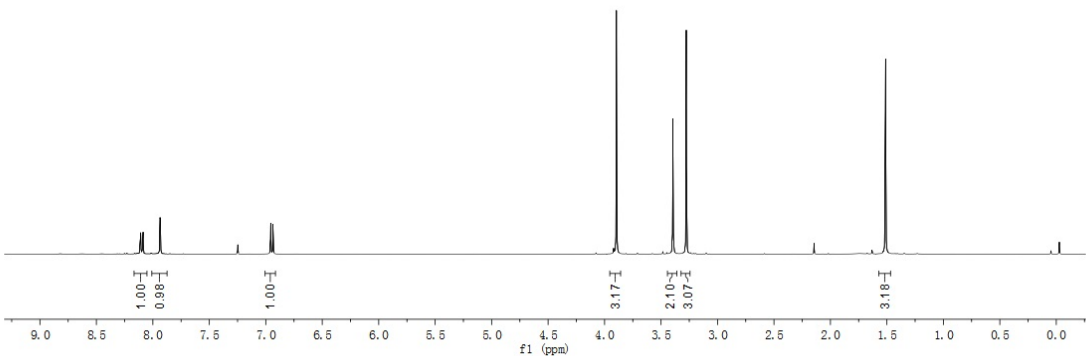
8.111
8.107
8.091
8.086
7.938
7.933
7.248
6.956
6.936
3.895
3.395
3.277
1.512
-0.027
1.00
0.98
1.00
3.17
2.10
3.07
3.18
f1 (ppm)

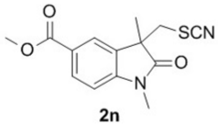
SCN
O
O
O
N

2n

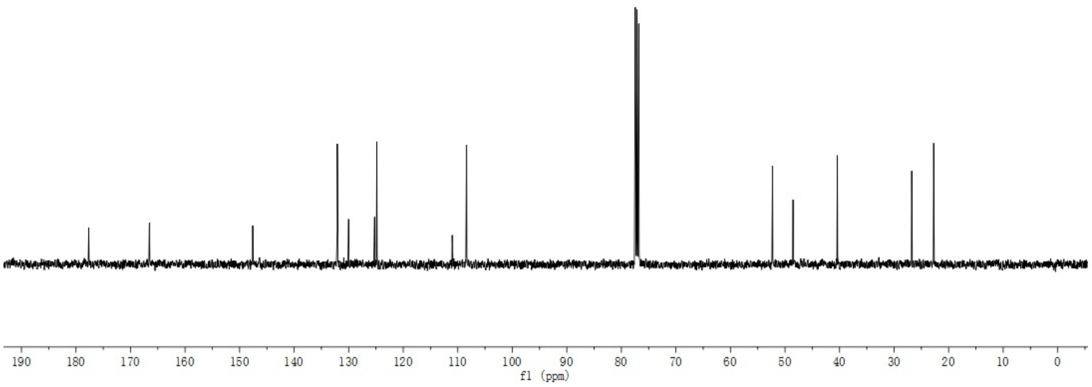
177.658
166.537
147.593
132.080
130.018
125.256
124.833
111.021
108.385
77.453
77.137
76.819
52.280
48.521
40.401
26.776
22.742
f1 (ppm)

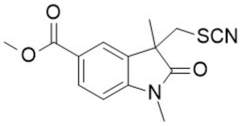
SCN
O
O
O
N

2n

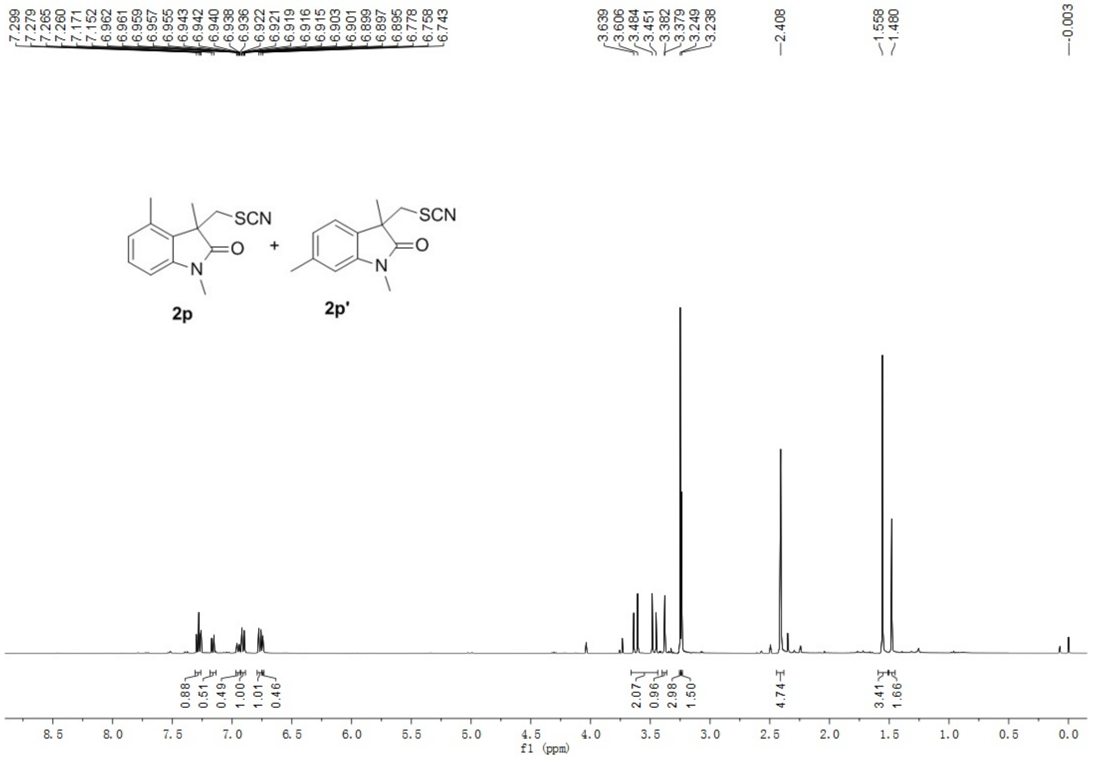

7.299
7.279
7.265
7.260
7.171
7.152
6.962
6.961
6.959
6.957
6.955
6.943
6.942
6.940
6.938
6.936
6.922
6.921
6.919
6.916
6.915
6.903
6.901
6.899
6.897
6.895
6.778
6.758
6.743
3.639
3.606
3.484
3.451
3.382
3.379
3.249
3.238
2.408
1.558
1.480
-0.003
SCN
O
N
+
2p
2p'
0.88
0.51
0.49
1.00
1.01
0.46
2.07
0.96
2.98
1.50
4.74
3.41
1.66
8.5 8.0 7.5 7.0 6.5 6.0 5.5 5.0 4.5 4.0 3.5 3.0 2.5 2.0 1.5 1.0 0.5 0.0
f1 (ppm)

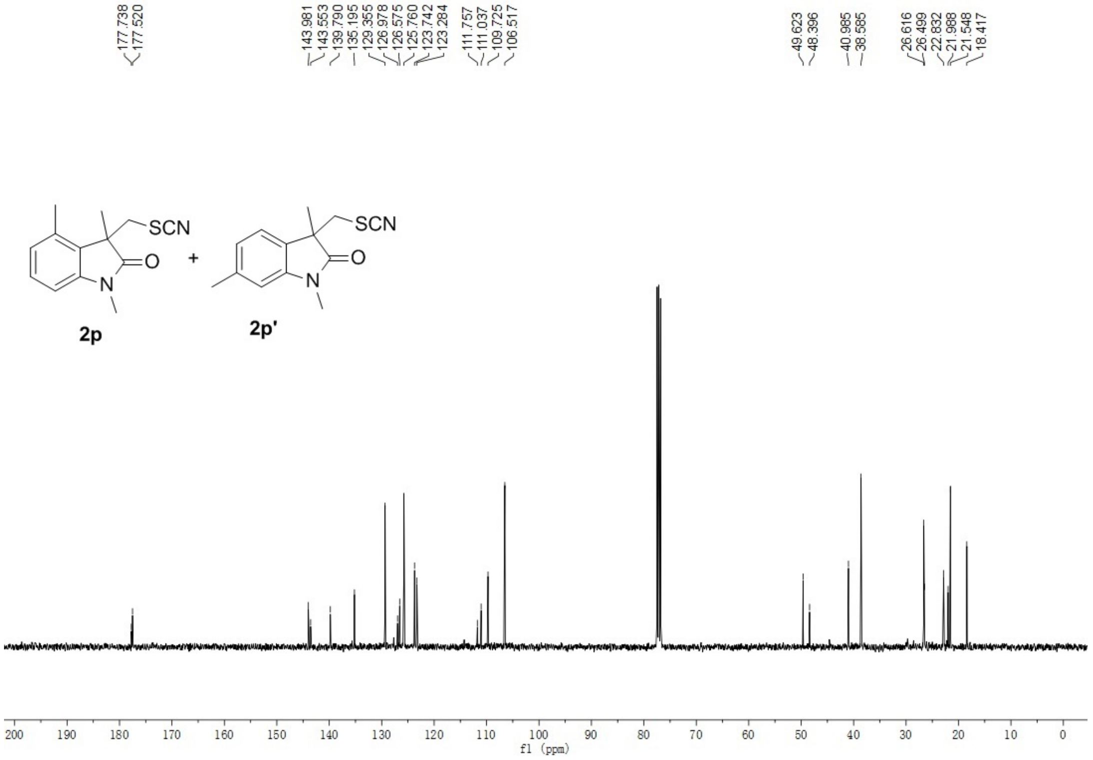

177.738
177.520
143.981
143.553
139.790
135.195
129.355
126.978
126.575
125.760
123.742
123.284
111.757
111.037
109.725
106.517
49.623
48.396
40.985
38.585
26.616
26.499
22.832
21.988
21.548
18.417
SCN
O
N
+
2p
2p'
200 190 180 170 160 150 140 130 120 110 100 90 80 70 60 50 40 30 20 10 0
f1 (ppm)

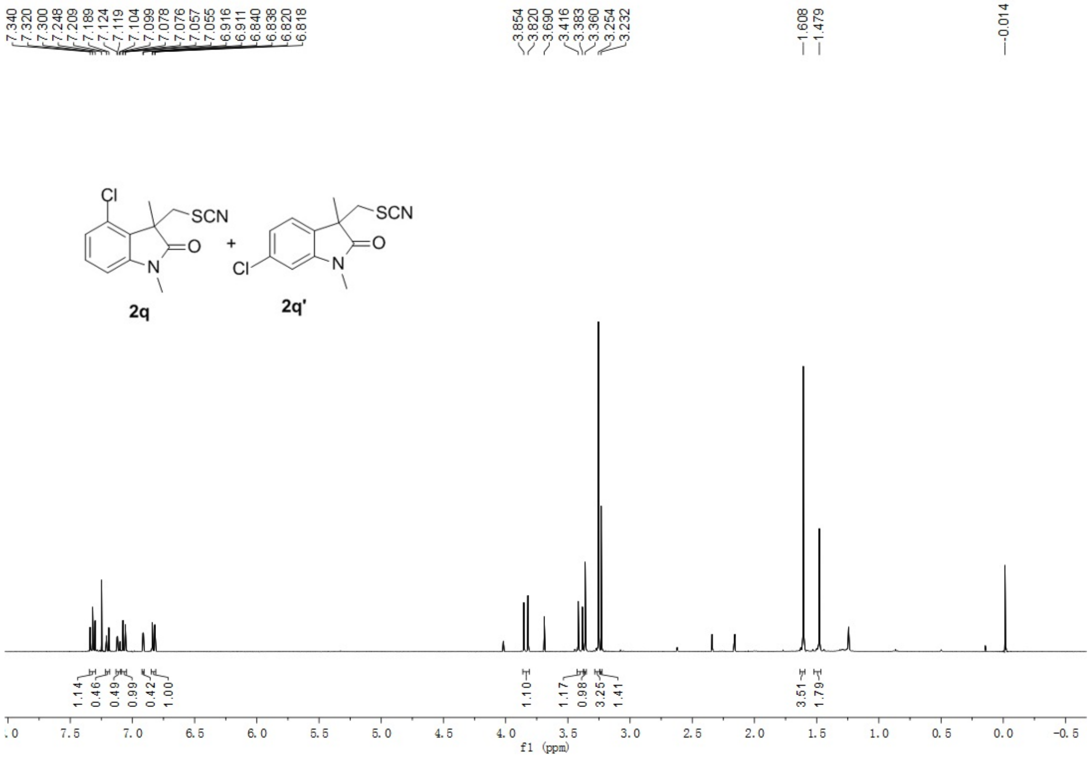

7.340 7.320 7.300 7.248 7.209 7.189 7.124 7.119 7.104 7.099 7.078 7.076 7.057 7.055 6.916 6.911 6.840 6.838 6.820 6.818
3.854 3.820 3.690 3.416 3.383 3.360 3.254 3.232
1.608 1.479
-0.014
Cl
SCN
O
N
2q
2q'
1.14 0.46 0.49 0.99 0.42 1.00
1.10
1.17 0.98 3.25 1.41
3.51 1.79
f1 (ppm)

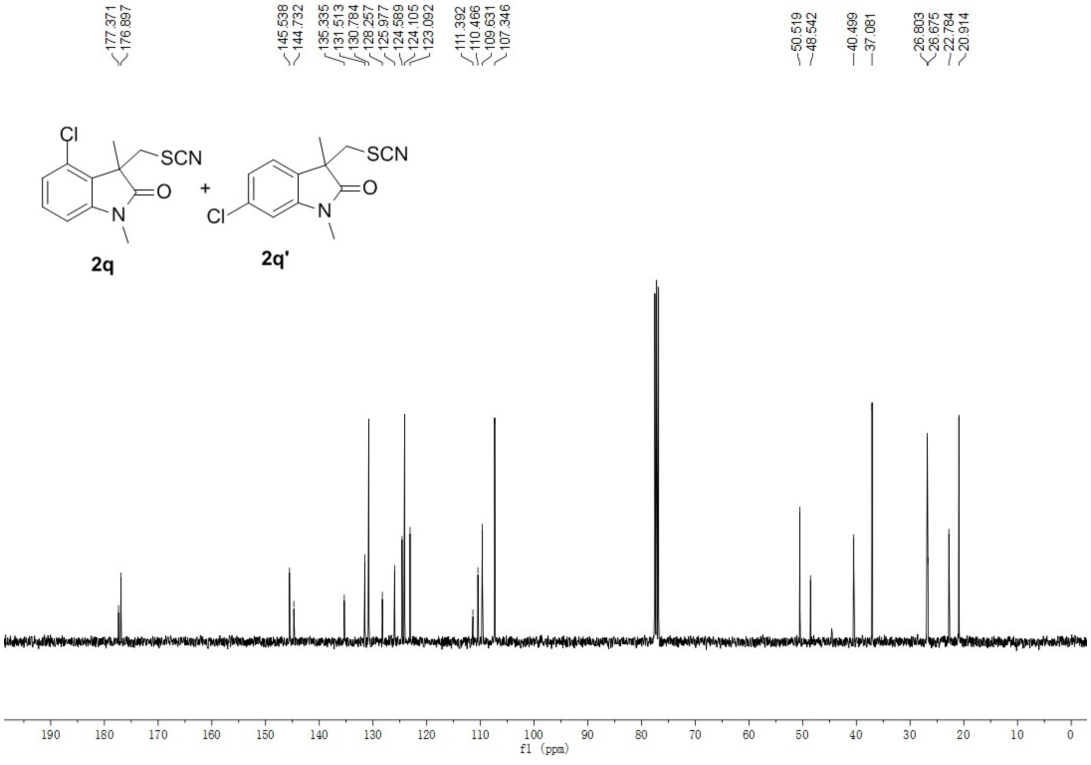

177.371 176.897
145.538 144.732 135.335 131.513 130.784 128.257 125.977 124.589 124.105 123.092
111.392 110.466 109.631 107.346
50.519 48.542
40.499 37.081
26.803 26.675 22.784 20.914
Cl
SCN
O
N
2q
2q'
f1 (ppm)

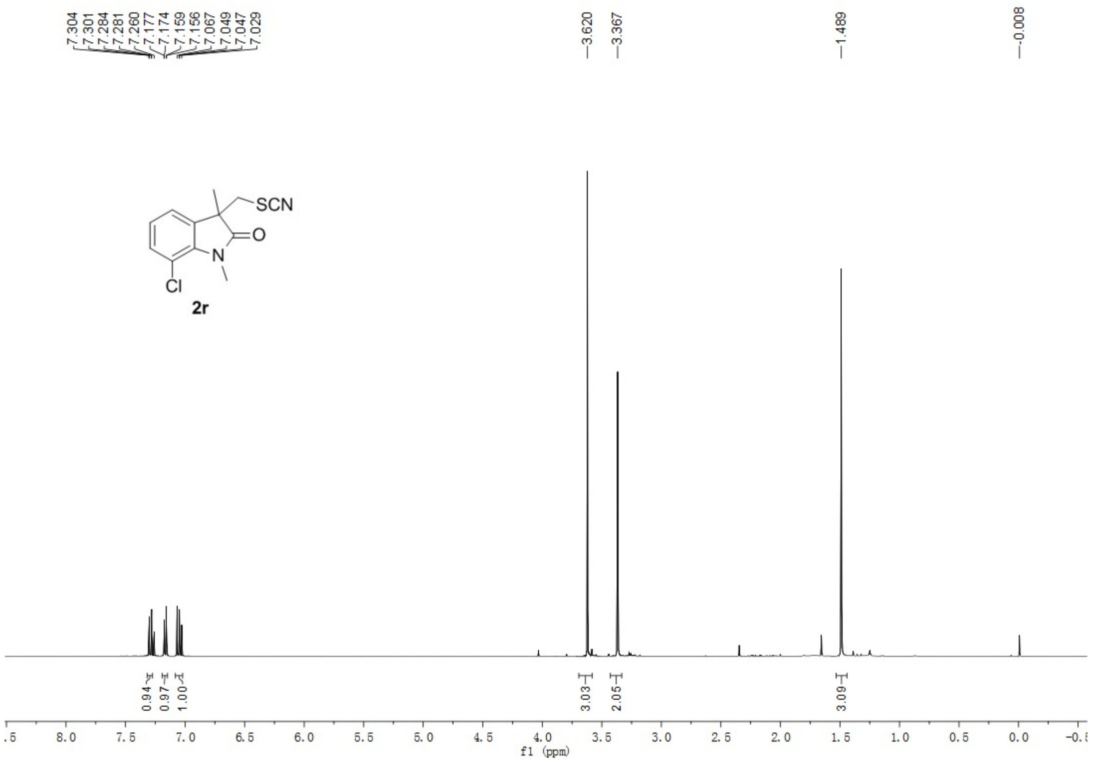
7.304
7.301
7.284
7.281
7.260
7.177
7.174
7.159
7.156
7.067
7.049
7.047
7.029
3.620
3.367
1.489
-0.008
SCN
O
N
Cl
2r
0.94
0.97
1.00
3.03
2.05
3.09
f1 (ppm)

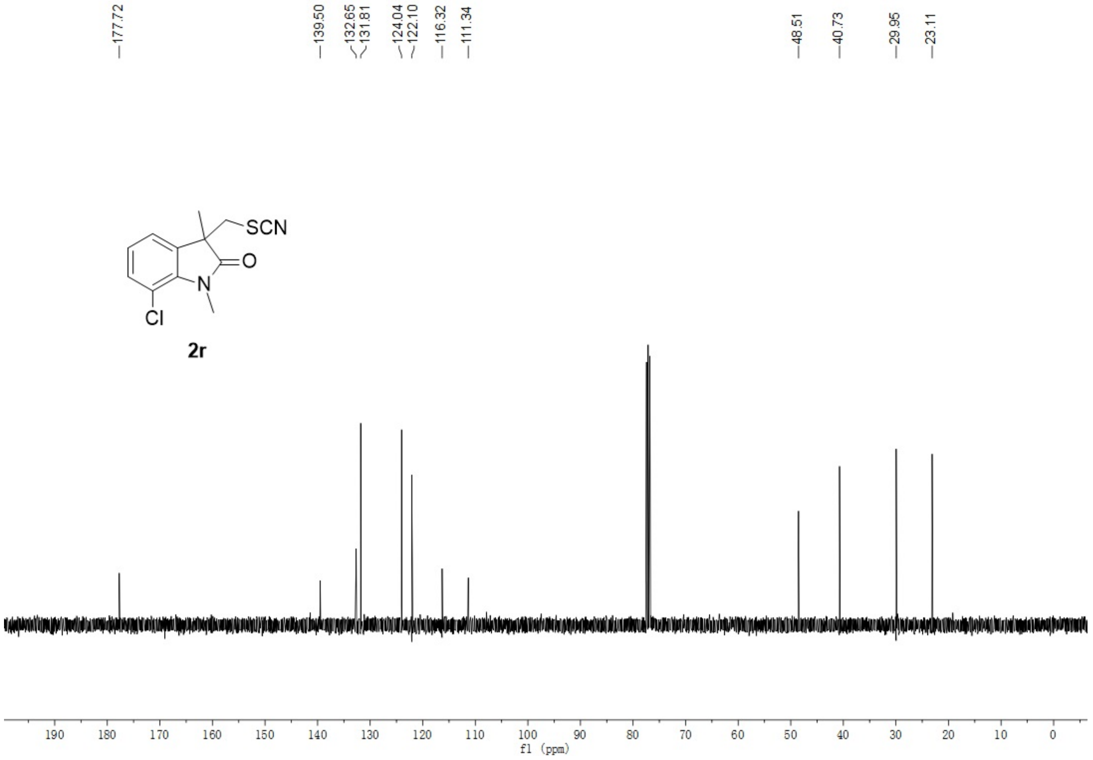
177.72
139.50
132.65
131.81
124.04
122.10
116.32
111.34
48.51
40.73
29.95
23.11
SCN
O
N
Cl
2r
f1 (ppm)

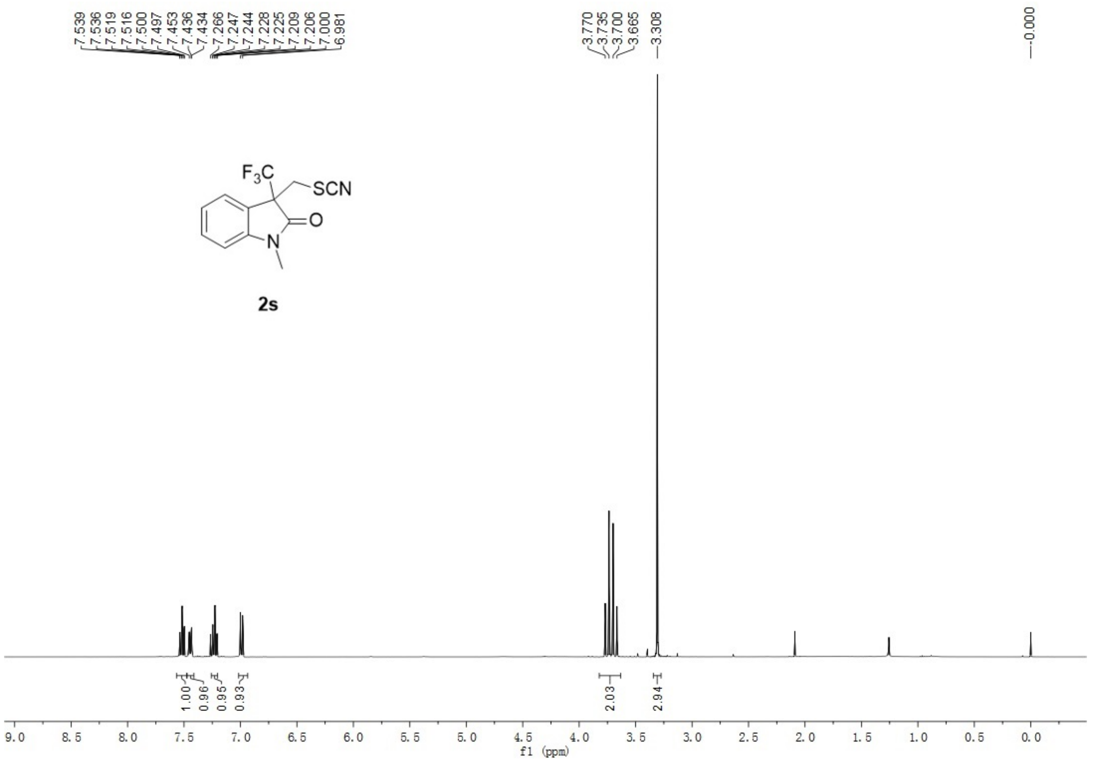
7.539
7.536
7.519
7.516
7.500
7.497
7.453
7.436
7.434
7.266
7.247
7.244
7.228
7.225
7.209
7.206
7.000
6.981
3.770
3.735
3.700
3.665
3.308
0.000
F_3C
SCN
O
N
2s
1.00
0.96
0.95
0.93
2.03
2.94
9.0 8.5 8.0 7.5 7.0 6.5 6.0 5.5 5.0 4.5 4.0 3.5 3.0 2.5 2.0 1.5 1.0 0.5 0.0
f1 (ppm)

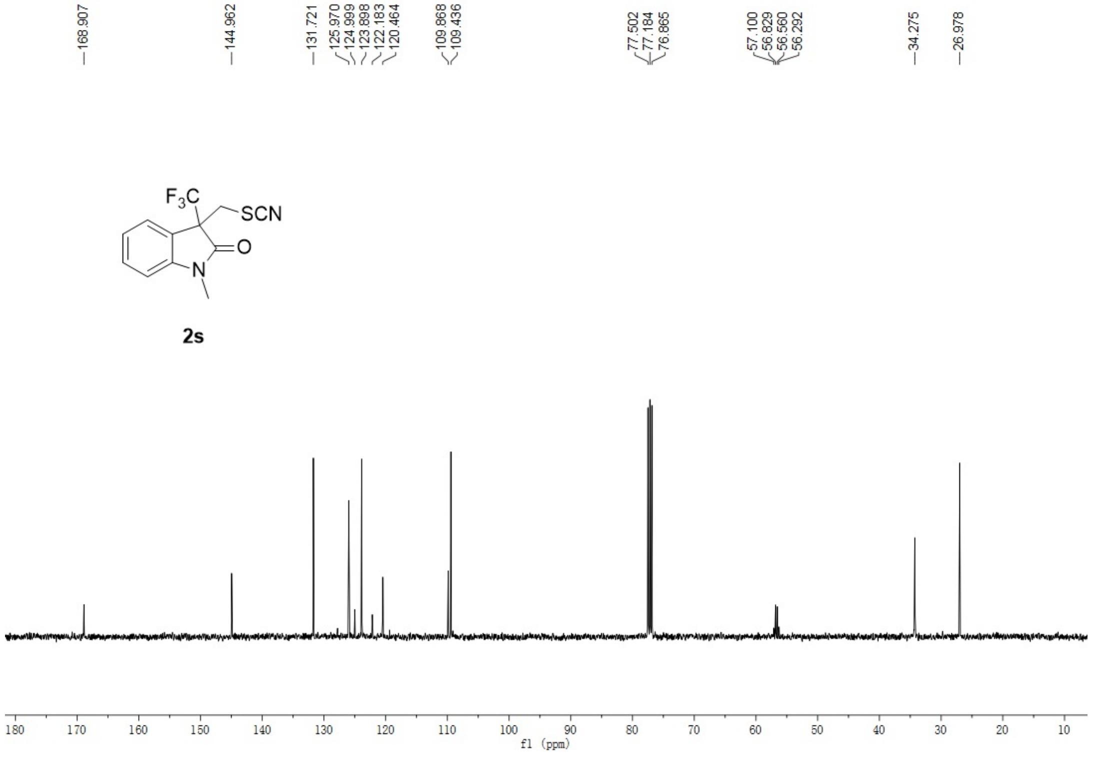
168.907
144.962
131.721
125.970
124.999
123.898
122.183
120.464
109.868
109.436
77.502
77.184
76.865
57.100
56.829
56.560
56.292
34.275
26.978
F_3C
SCN
O
N
2s
180 170 160 150 140 130 120 110 100 90 80 70 60 50 40 30 20 10
f1 (ppm)

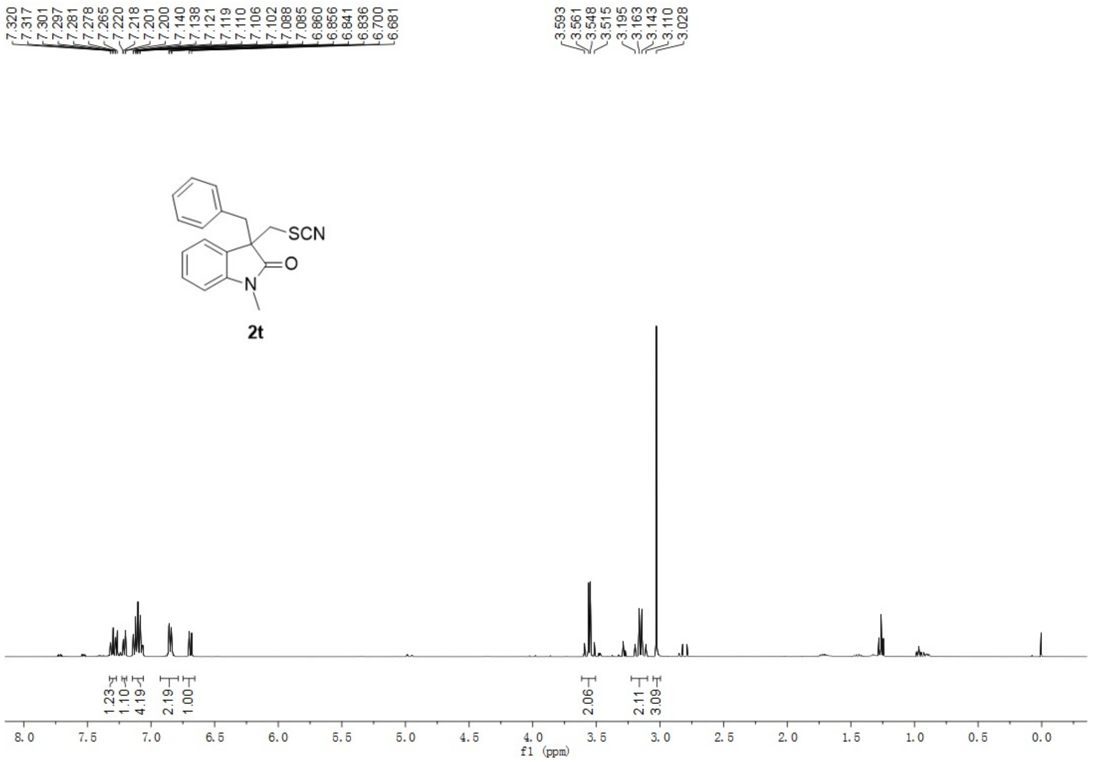
7.320
7.317
7.301
7.297
7.281
7.278
7.265
7.220
7.218
7.201
7.200
7.140
7.138
7.121
7.119
7.110
7.106
7.102
7.088
7.085
6.860
6.856
6.841
6.836
6.700
6.681
3.593
3.561
3.548
3.515
3.195
3.163
3.143
3.110
3.028
SCN
O
N
2t
1.23
1.10
4.19
2.19
1.00
2.06
2.11
3.09
f1 (ppm)

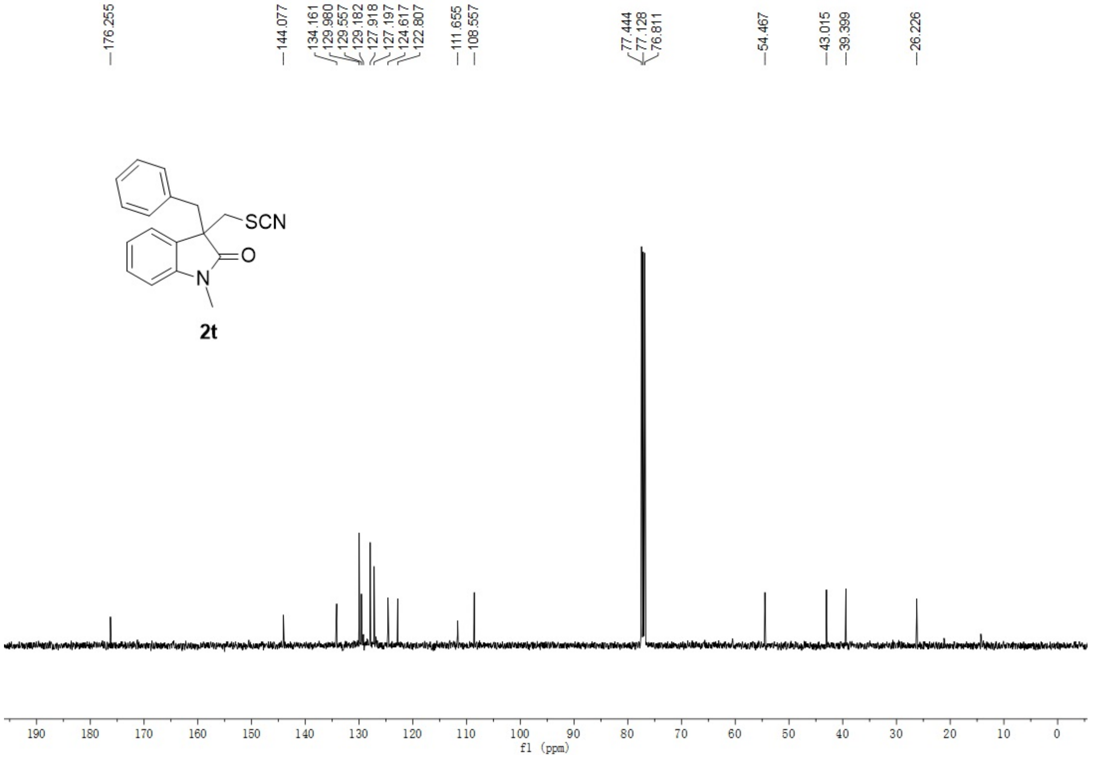
176.255
144.077
134.161
129.980
129.557
129.182
127.918
127.197
124.617
122.807
111.655
108.557
77.444
77.128
76.811
54.467
43.015
39.399
26.226
SCN
O
N
2t
f1 (ppm)

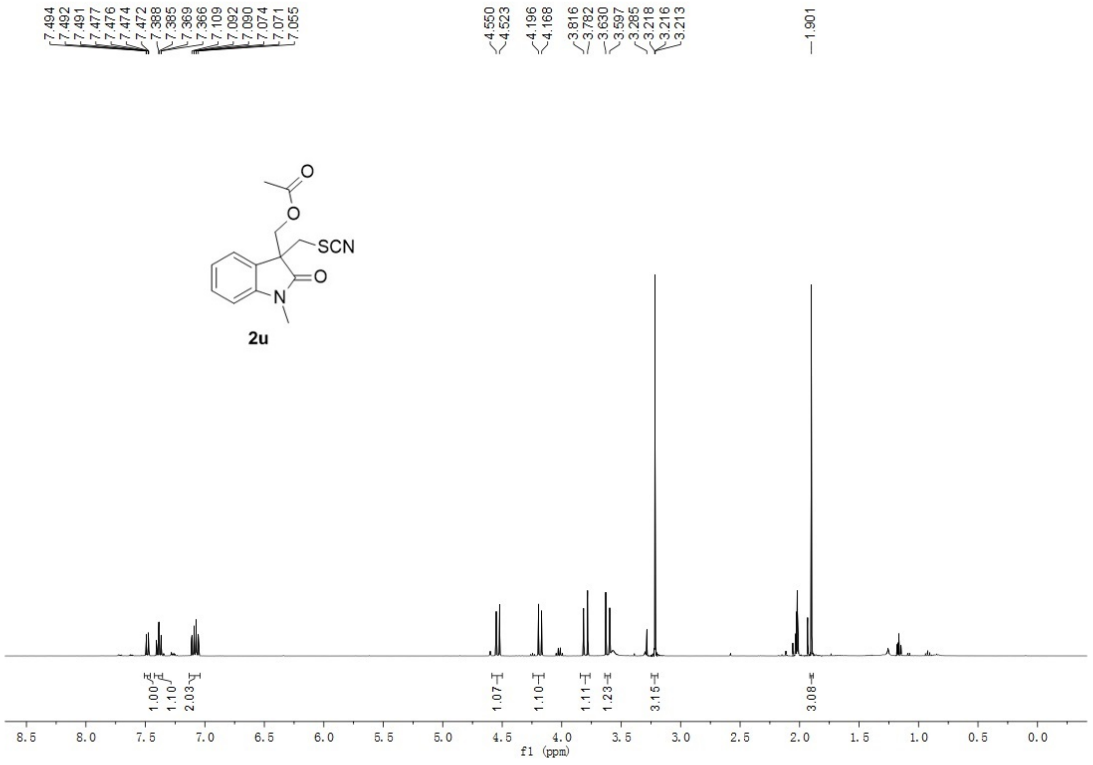
7.494
7.492
7.491
7.477
7.476
7.474
7.472
7.388
7.385
7.369
7.366
7.109
7.092
7.090
7.074
7.071
7.055
4.550
4.523
4.196
4.168
3.816
3.782
3.630
3.597
3.285
3.218
3.216
3.213
1.901
SCN
O
N
2u
1.00
1.10
2.03
1.07
1.10
1.11
1.23
3.15
3.08
8.5 8.0 7.5 7.0 6.5 6.0 5.5 5.0 4.5 4.0 3.5 3.0 2.5 2.0 1.5 1.0 0.5 0.0
f1 (ppm)

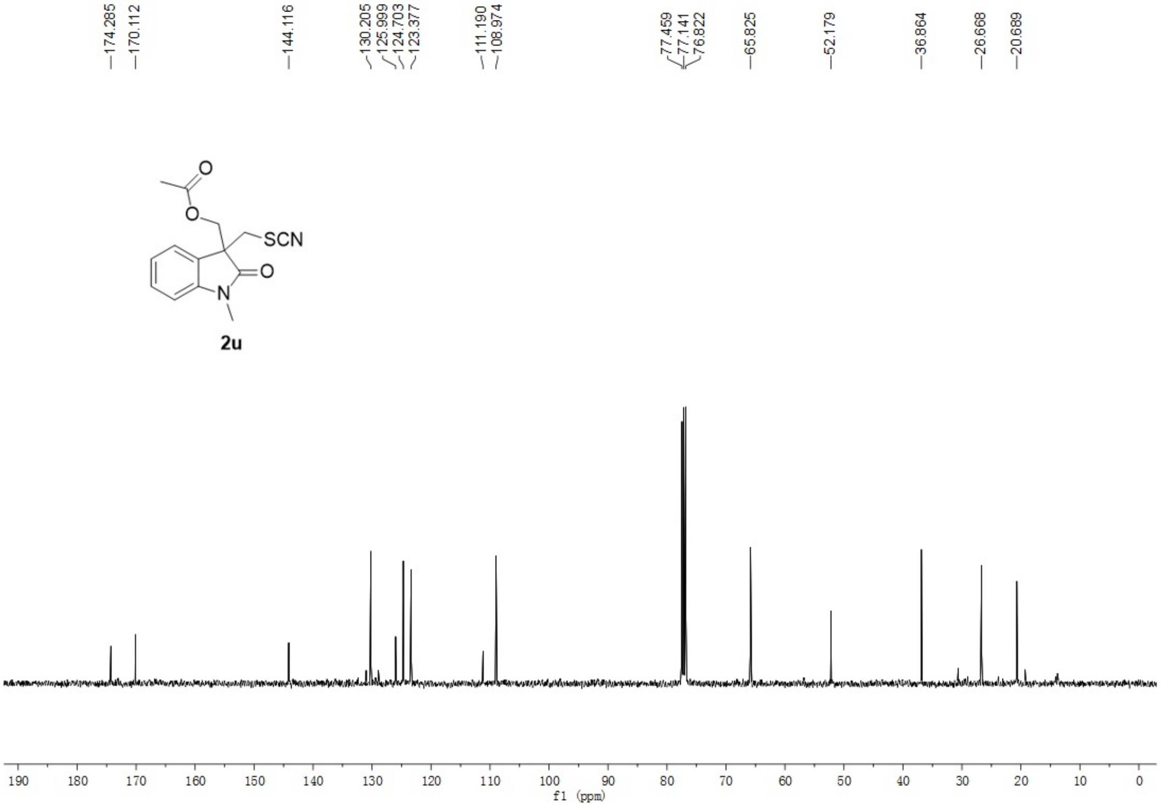
174.285
170.112
144.116
130.205
125.999
124.703
123.377
111.190
108.974
77.459
77.141
76.822
65.825
52.179
36.864
26.668
20.689
SCN
O
N
2u
190 180 170 160 150 140 130 120 110 100 90 80 70 60 50 40 30 20 10 0
f1 (ppm)

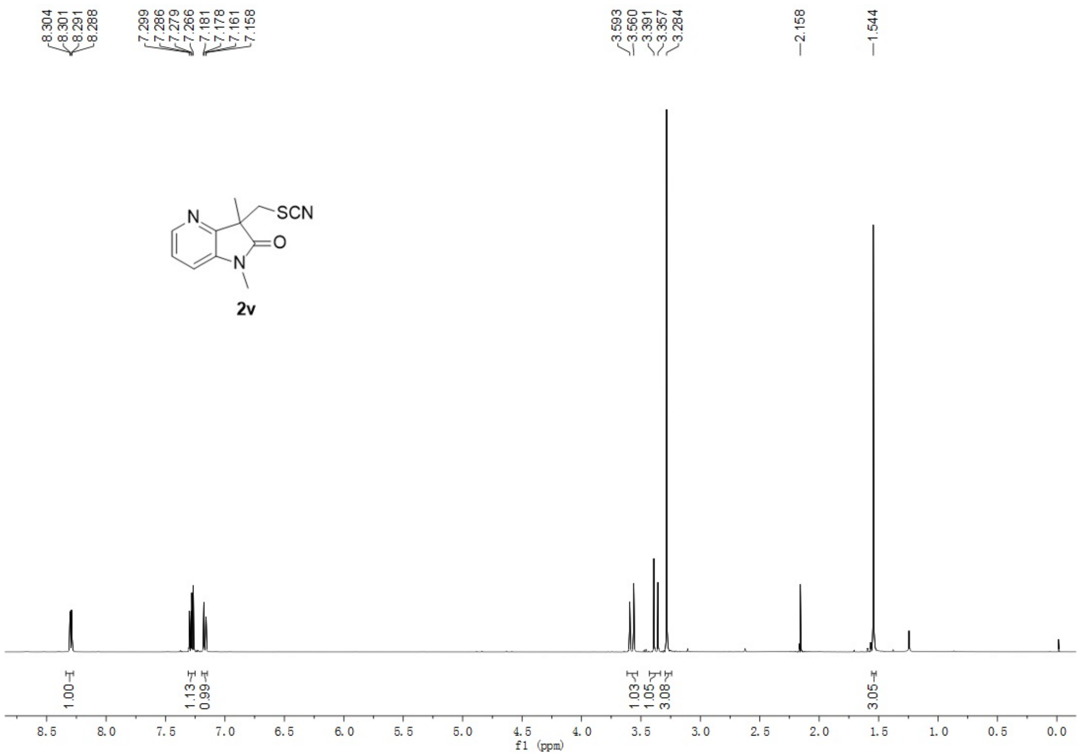

8.304
8.301
8.291
8.288
7.299
7.286
7.279
7.266
7.181
7.178
7.161
7.158
3.593
3.560
3.391
3.357
3.284
2.158
1.544
SCN
N
O
2v
1.00
1.13
0.99
1.03
1.05
3.08
3.05
8.5 8.0 7.5 7.0 6.5 6.0 5.5 5.0 4.5 4.0 3.5 3.0 2.5 2.0 1.5 1.0 0.5 0.0
f1 (ppm)

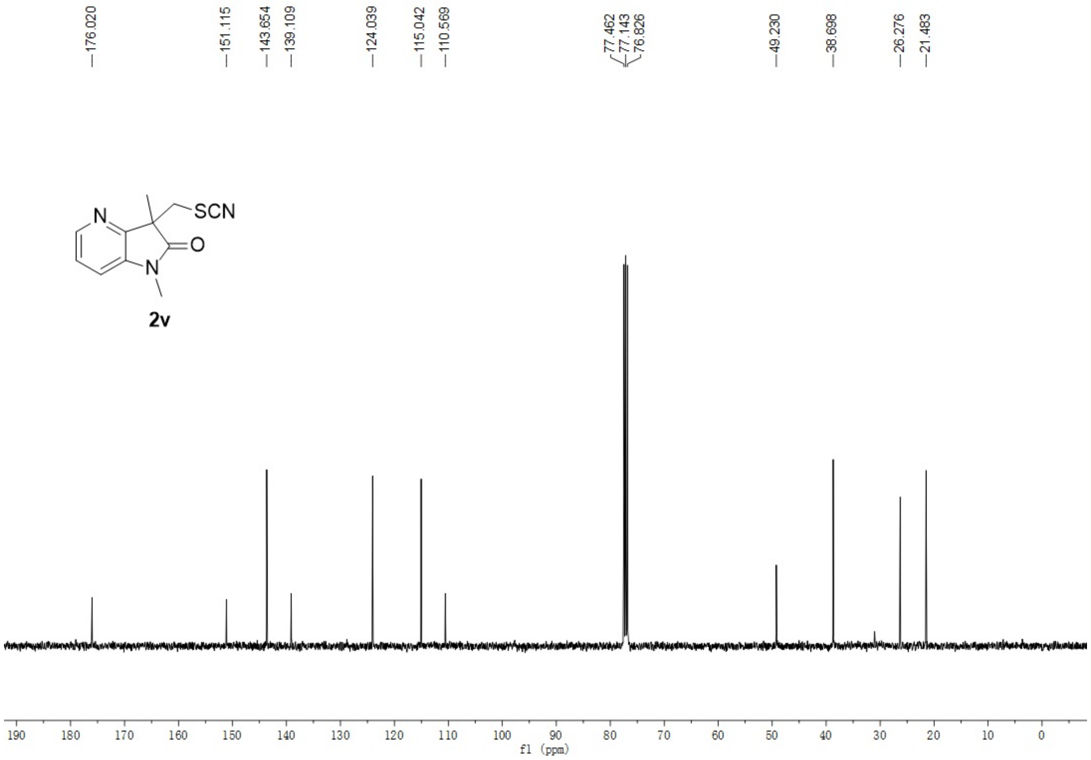

176.020
151.115
143.654
139.109
124.039
115.042
110.569
77.462
77.143
76.826
49.230
38.698
26.276
21.483
SCN
N
O
2v
190 180 170 160 150 140 130 120 110 100 90 80 70 60 50 40 30 20 10 0
f1 (ppm)

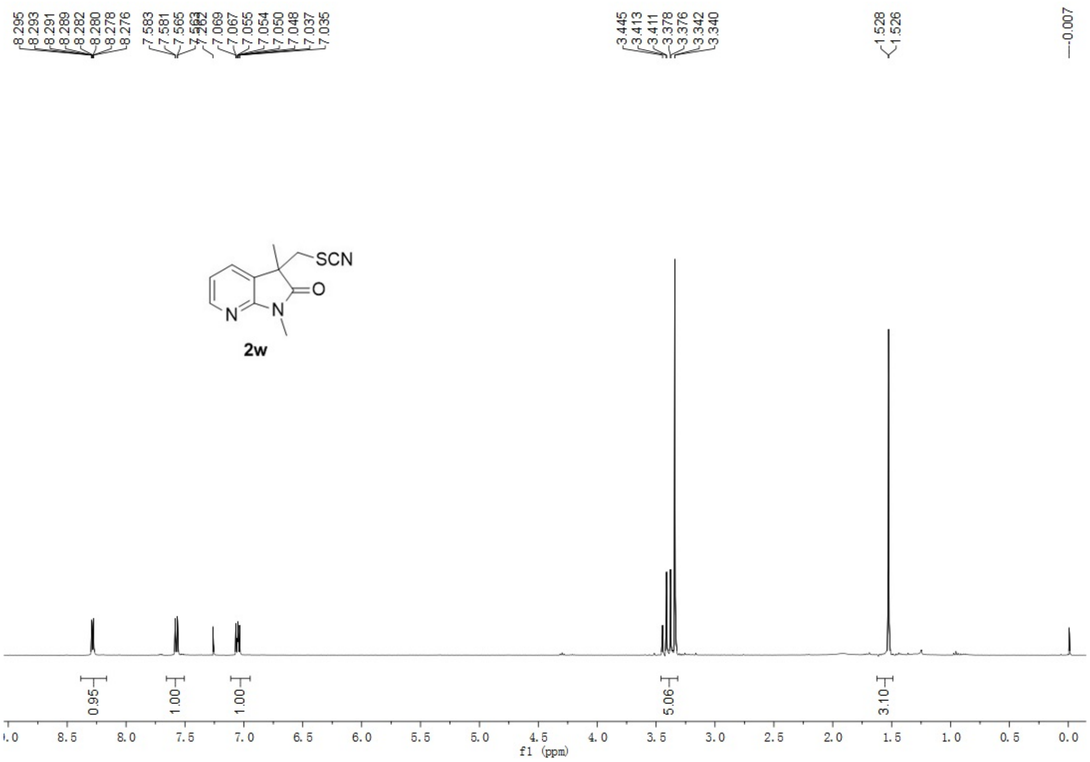

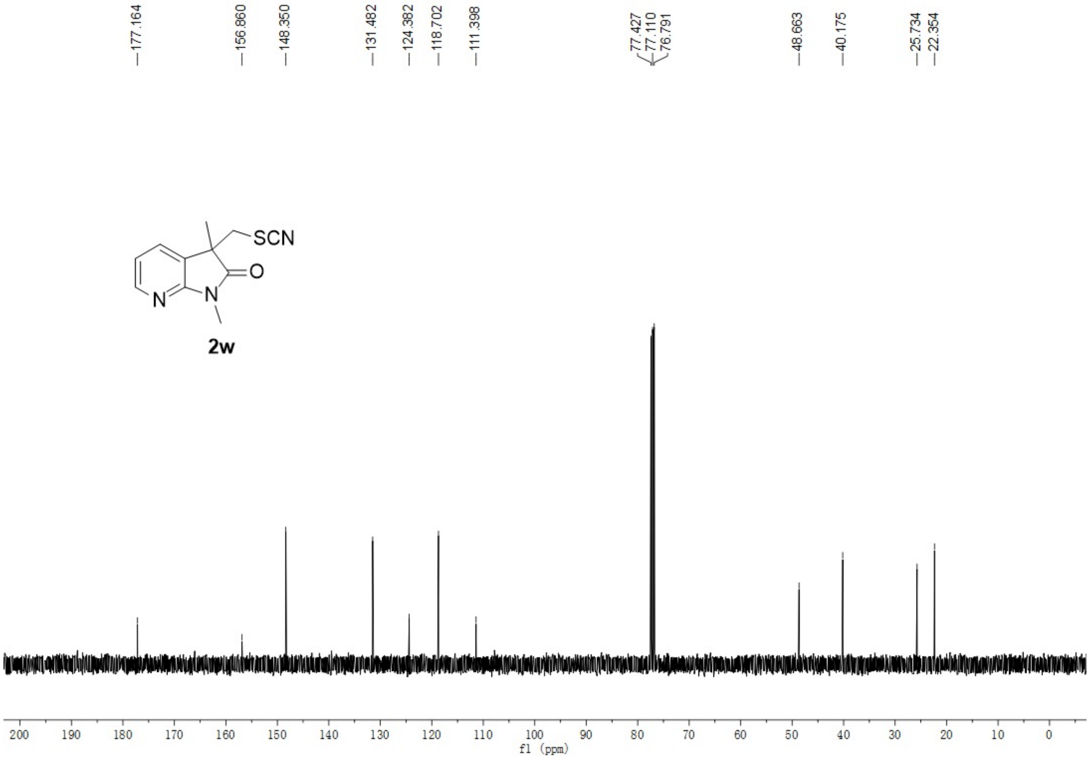

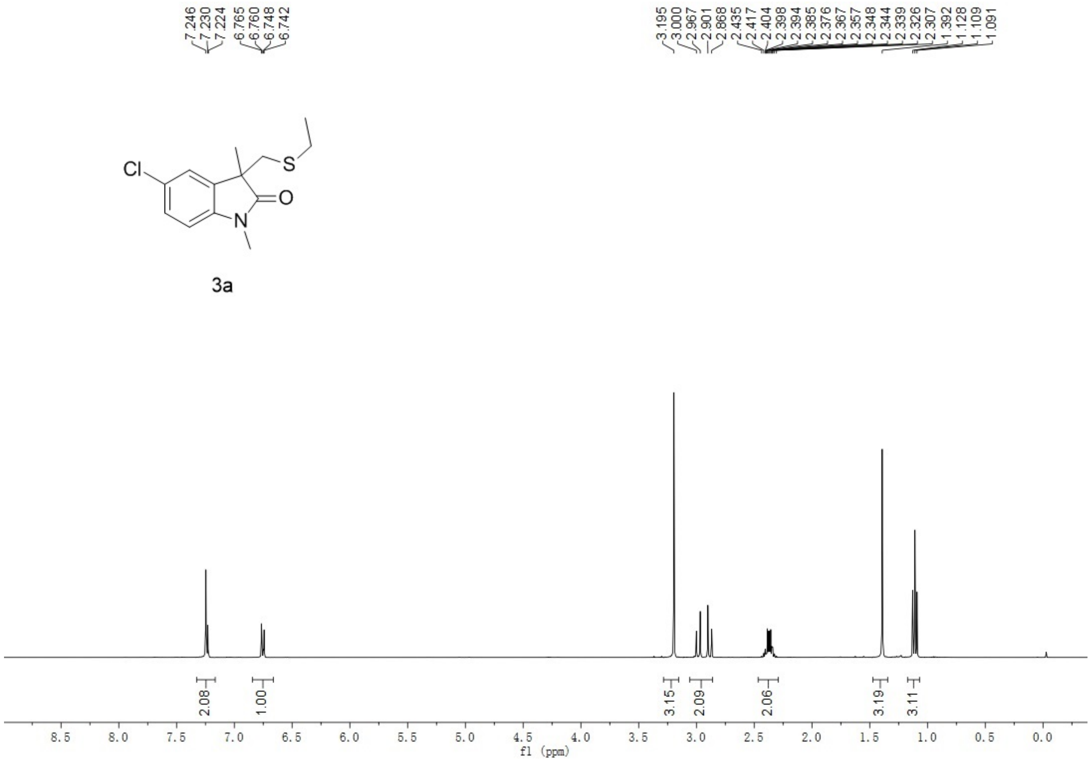

7.246
7.230
7.224
6.765
6.760
6.748
6.742
3.195
3.000
2.967
2.901
2.868
2.435
2.417
2.404
2.398
2.394
2.385
2.376
2.367
2.357
2.348
2.344
2.339
2.326
2.307
1.392
1.128
1.109
1.091
Cl
S
O
N
2.08
1.00
3.15
2.09
2.06
3.19
3.11
8.5 8.0 7.5 7.0 6.5 6.0 5.5 5.0 4.5 4.0 3.5 3.0 2.5 2.0 1.5 1.0 0.5 0.0
f1 (ppm)

3a

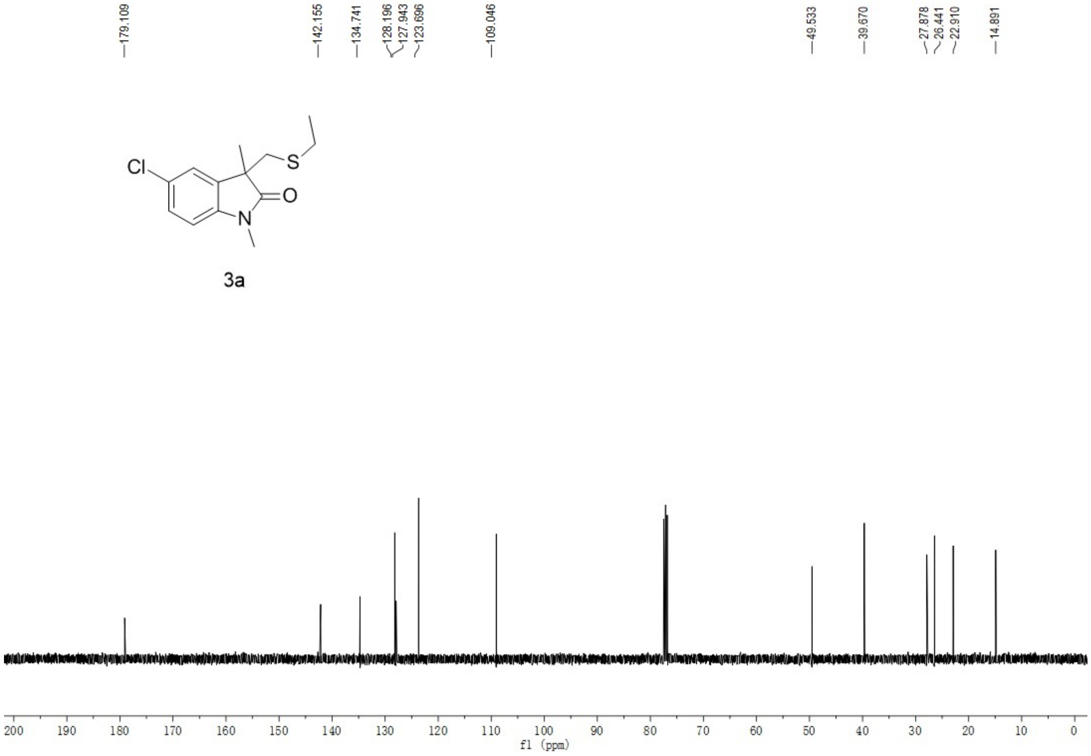

179.109
142.155
134.741
128.196
127.943
123.696
109.046
49.533
39.670
27.878
26.441
22.910
14.891
Cl
S
O
N
200 190 180 170 160 150 140 130 120 110 100 90 80 70 60 50 40 30 20 10 0
f1 (ppm)

3a

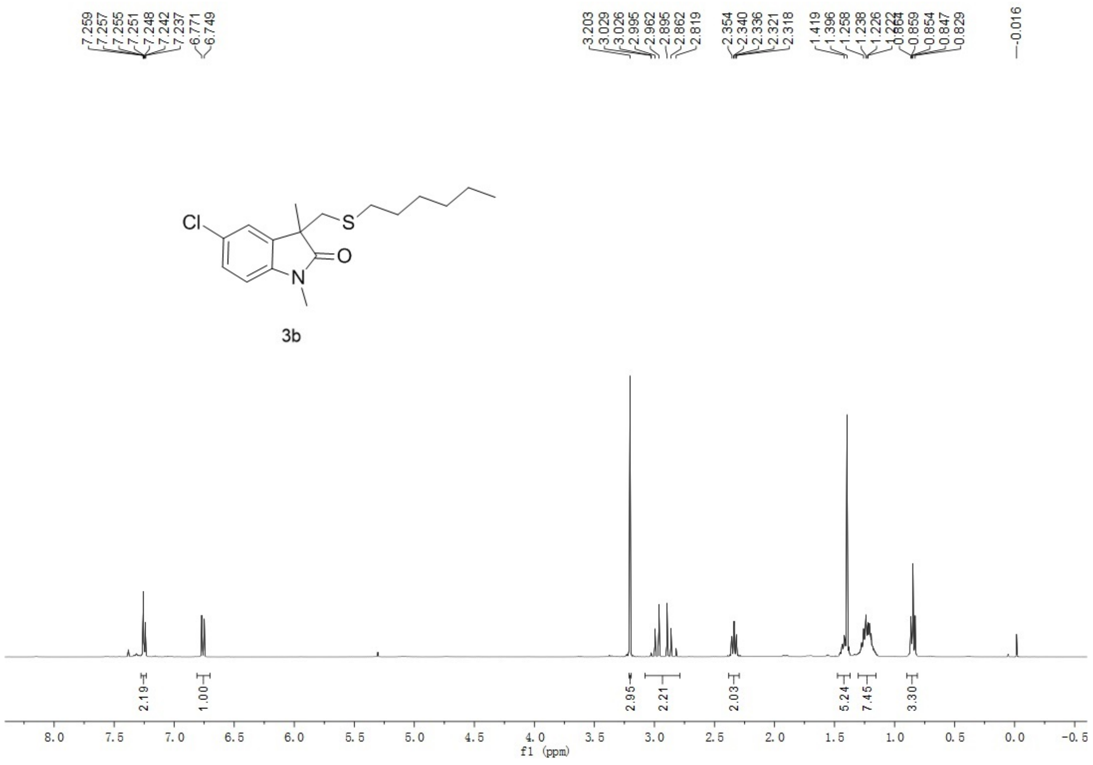

7.259
7.257
7.255
7.251
7.248
7.242
7.237
6.771
6.749
3.203
3.029
3.026
2.995
2.962
2.895
2.862
2.819
2.354
2.340
2.336
2.321
2.318
1.419
1.396
1.258
1.238
1.226
1.222
0.864
0.859
0.854
0.847
0.829
-0.016
Cl
S
O
N
3b
2.19
1.00
2.95
2.21
2.03
5.24
7.45
3.30
f1 (ppm)

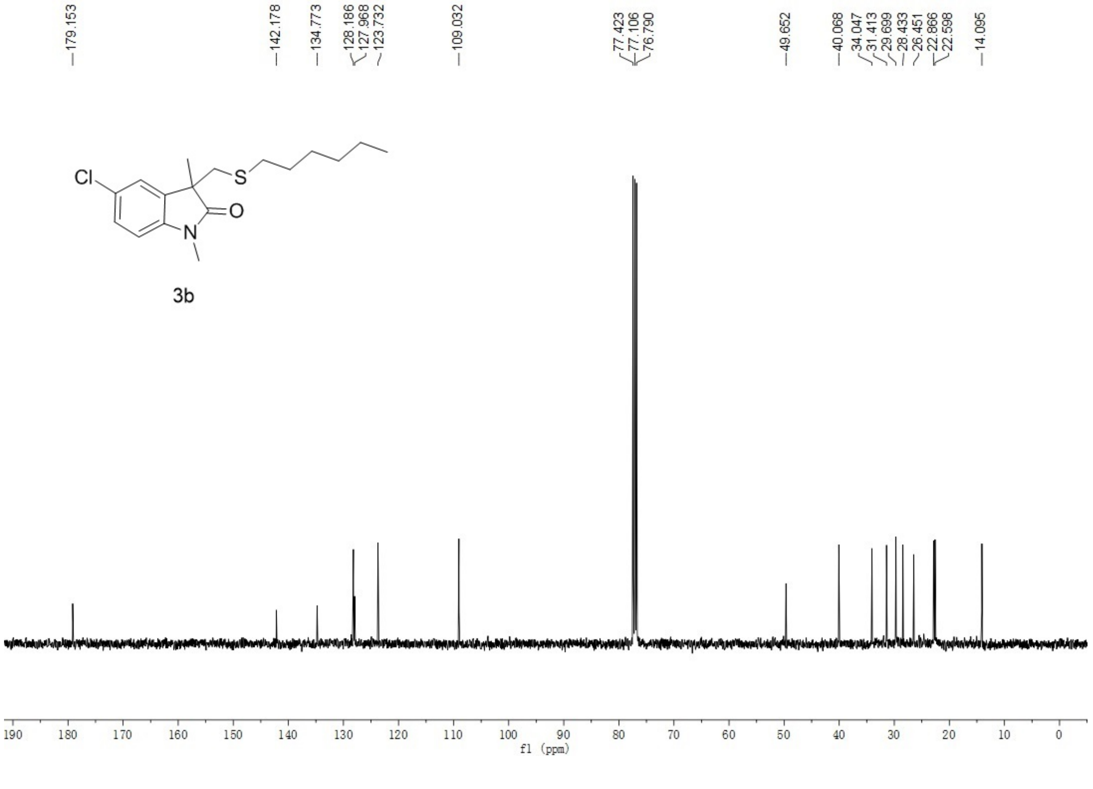

179.153
142.178
134.773
128.186
127.968
123.732
109.032
77.423
77.106
76.790
49.652
40.068
34.047
31.413
29.699
28.433
26.451
22.866
22.598
14.095
Cl
S
O
N
3b
f1 (ppm)

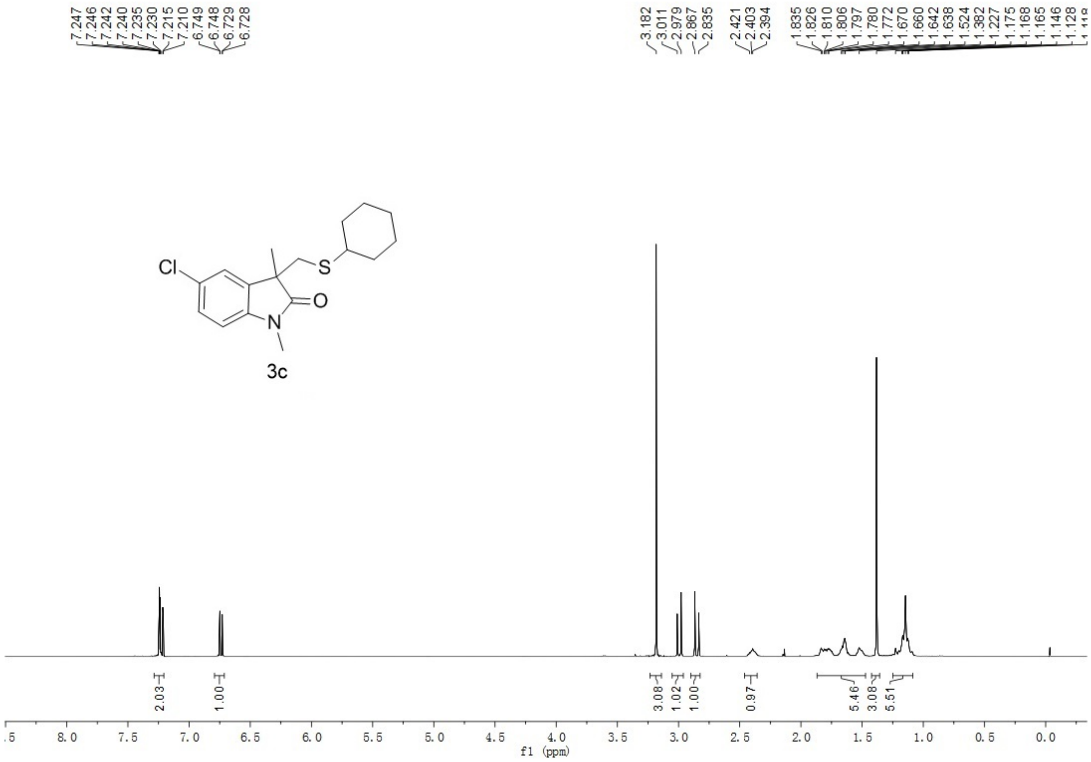
7.247
7.246
7.242
7.240
7.235
7.230
7.215
7.210
6.749
6.748
6.729
6.728
3.182
3.011
2.979
2.867
2.835
2.421
2.403
2.394
1.835
1.826
1.810
1.806
1.797
1.780
1.772
1.670
1.660
1.642
1.638
1.524
1.382
1.227
1.175
1.168
1.165
1.146
1.128
Cl
S
O
N
3c
2.03
1.00
3.08
1.02
1.00
0.97
5.46
3.08
5.51
f1 (ppm)

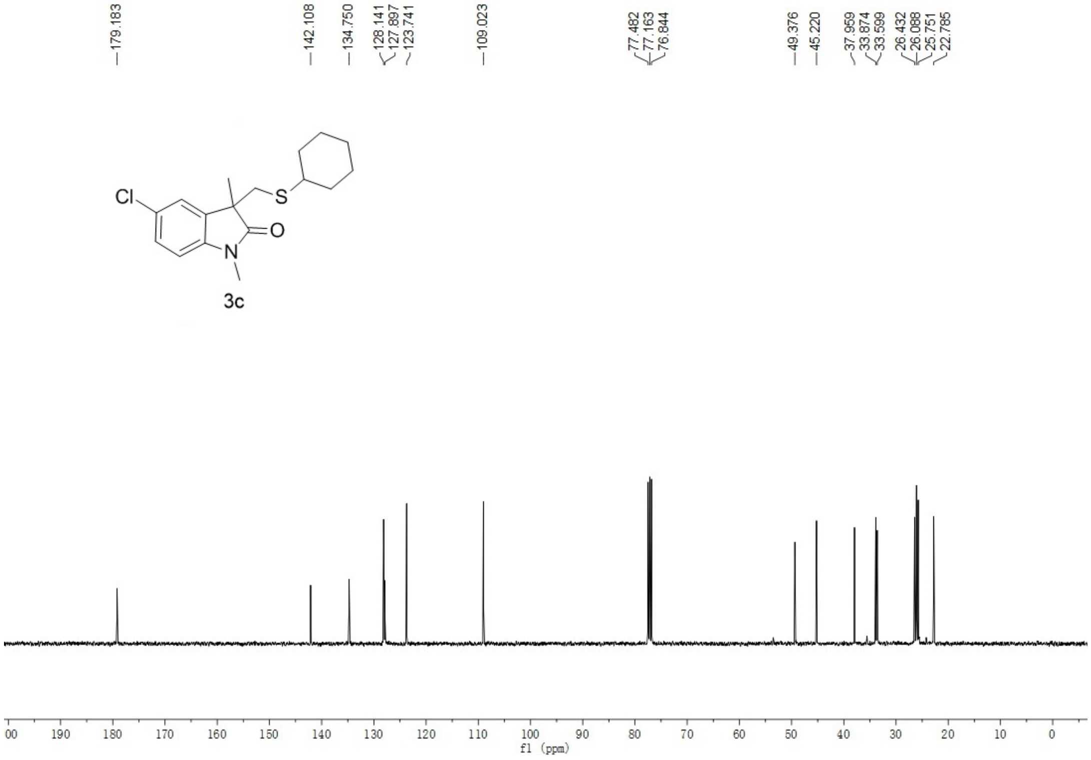
179.183
142.108
134.750
128.141
127.897
123.741
109.023
77.482
77.163
76.844
49.376
45.220
37.959
33.874
33.599
26.432
26.088
25.751
22.785
Cl
S
O
N
3c
f1 (ppm)

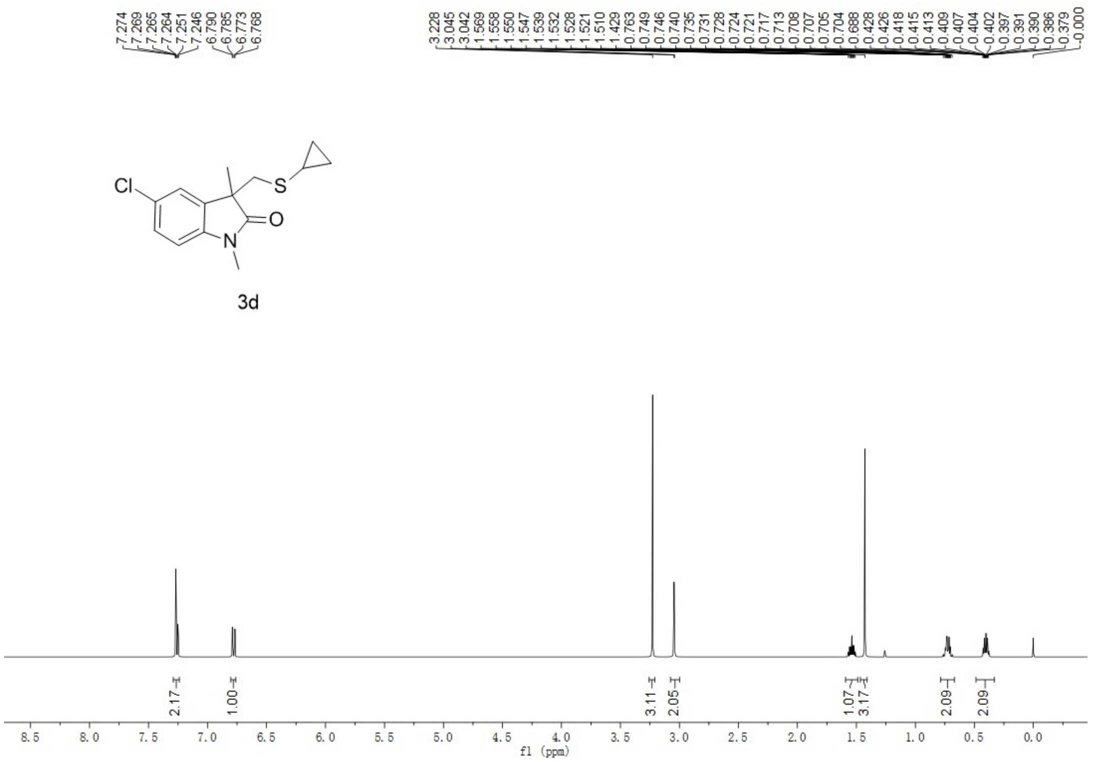
7.274
7.269
7.265
7.264
7.251
7.246
6.790
6.785
6.773
6.768
3.228
3.045
3.042
1.569
1.558
1.550
1.547
1.539
1.532
1.528
1.521
1.510
1.429
0.763
0.749
0.746
0.740
0.735
0.731
0.728
0.724
0.721
0.717
0.713
0.708
0.707
0.705
0.704
0.688
0.428
0.426
0.418
0.415
0.413
0.409
0.407
0.404
0.402
0.397
0.391
0.390
0.386
0.379
0.000
Cl
S
O
N
3d
2.17
1.00
3.11
2.05
1.07
3.17
2.09
2.09
f1 (ppm)

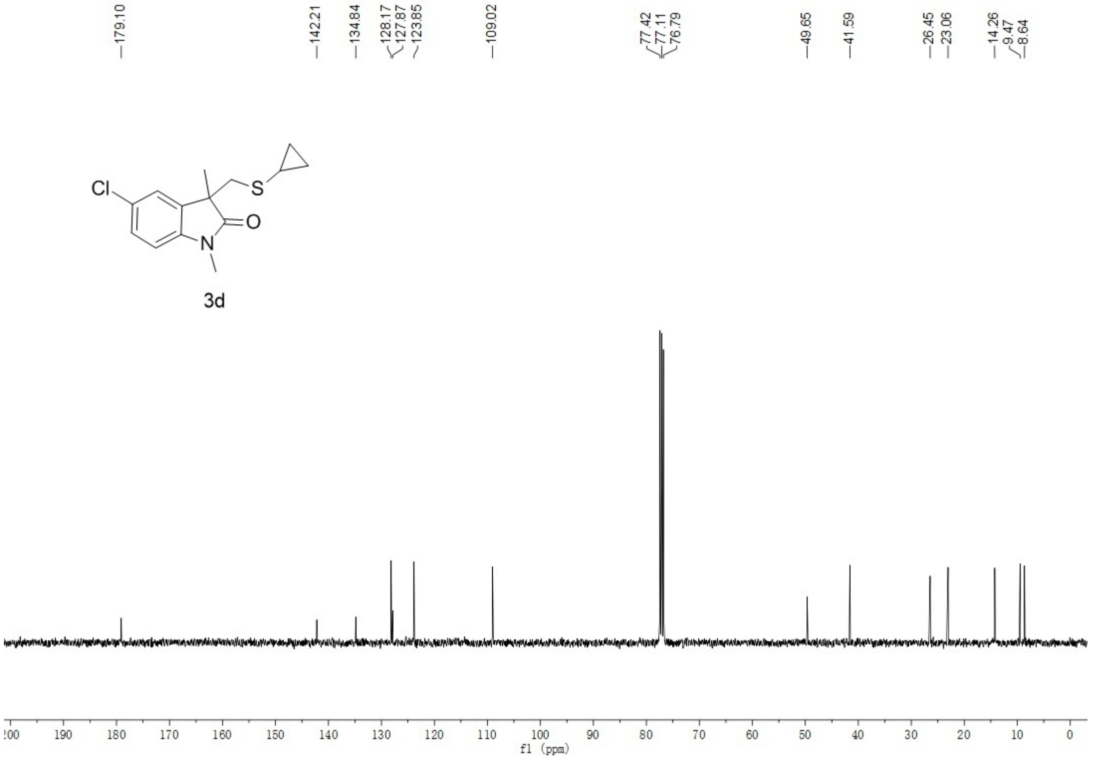
179.10
142.21
134.84
128.17
127.87
123.85
109.02
77.42
77.11
76.79
49.65
41.59
26.45
23.06
14.26
9.47
8.64
Cl
S
O
N
3d
f1 (ppm)

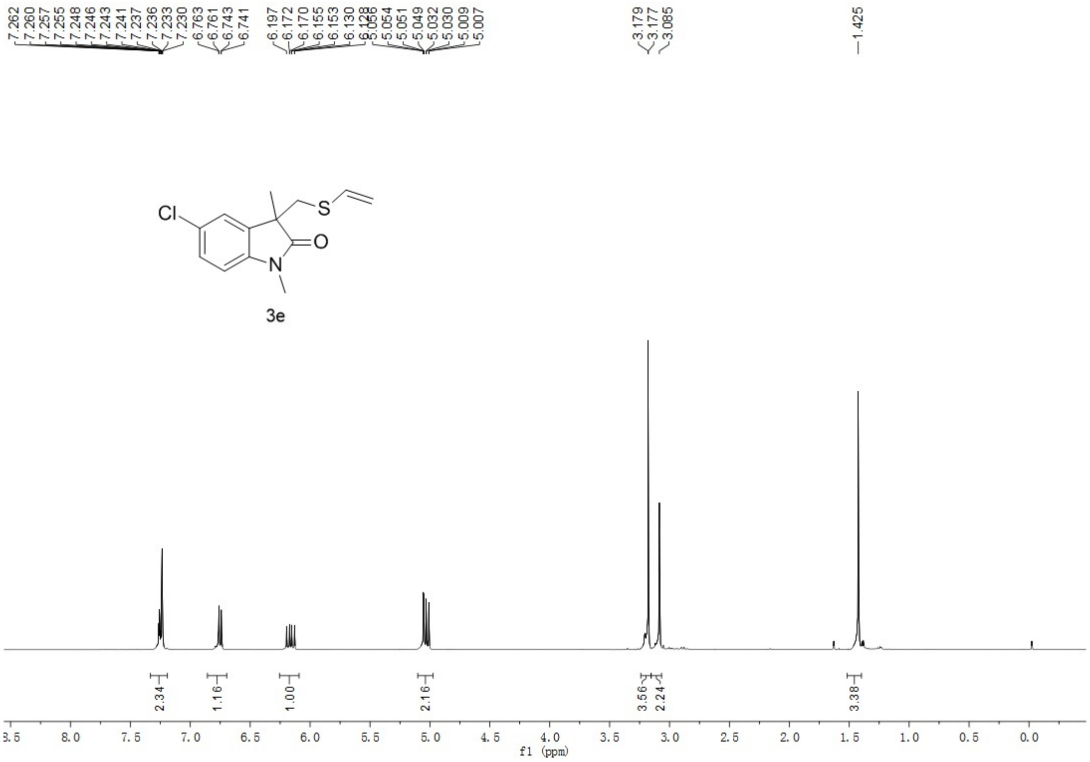
7.262
7.260
7.257
7.255
7.248
7.246
7.243
7.241
7.237
7.236
7.233
7.230
6.763
6.761
6.743
6.741
6.197
6.172
6.170
6.155
6.153
6.130
5.054
5.051
5.049
5.032
5.030
5.009
5.007
3.179
3.177
3.085
1.425
Cl
S
O
N
3e
2.34
1.16
1.00
2.16
3.56
2.24
3.38
f1 (ppm)

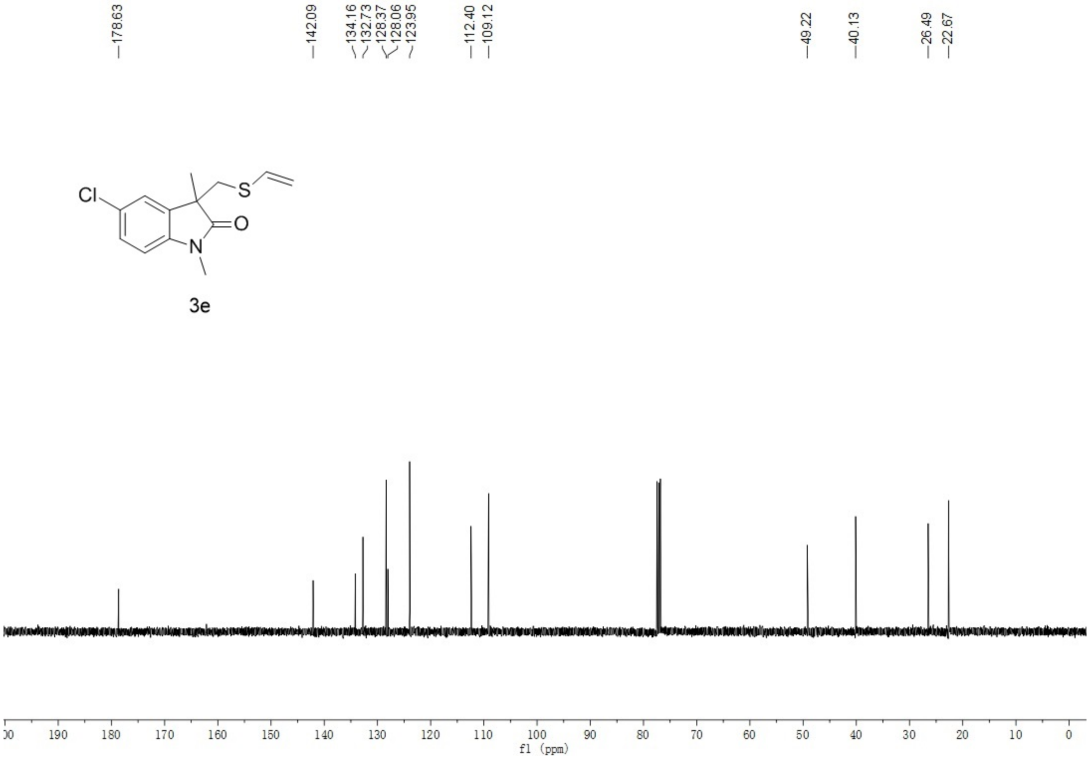
178.63
142.09
134.16
132.73
128.37
128.06
123.95
112.40
109.12
49.22
40.13
26.49
22.67
Cl
S
O
N
3e
f1 (ppm)

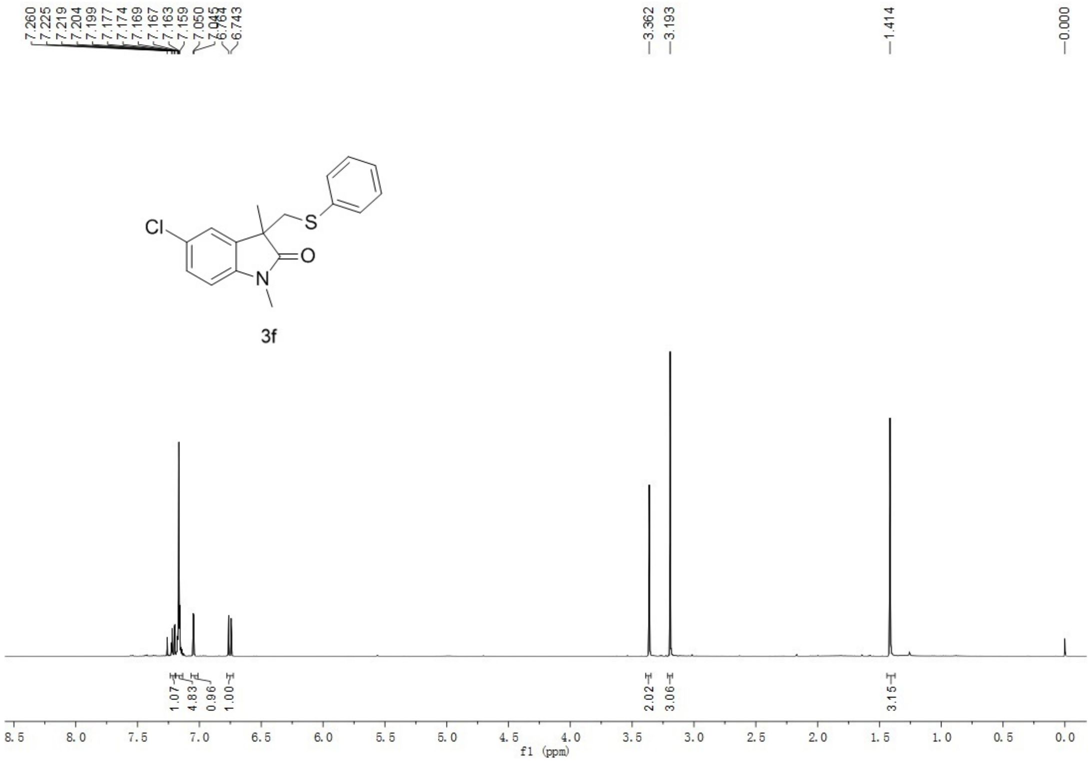
7.260
7.225
7.219
7.204
7.199
7.177
7.174
7.169
7.167
7.163
7.159
7.050
7.045
6.764
6.743
3.362
3.193
1.414
0.000
Cl
S
O
N
3f
1.07
4.83
0.96
1.00
2.02
3.06
3.15
f1 (ppm)

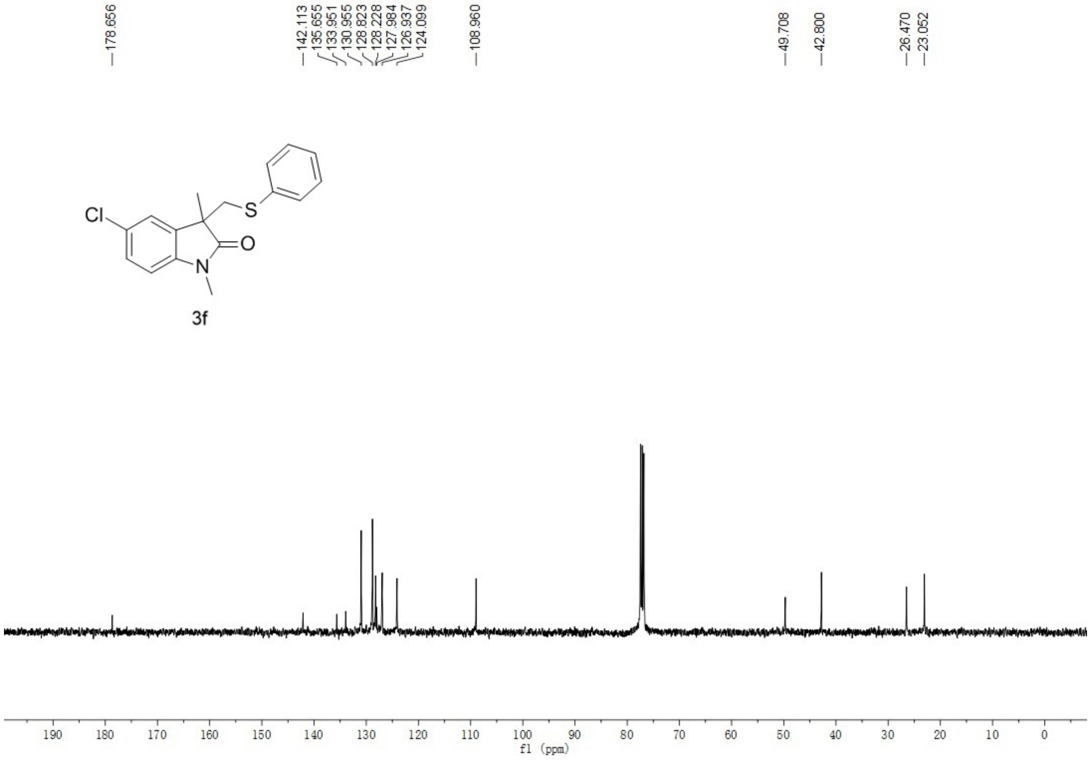
178.656
142.113
135.655
133.951
130.955
128.823
128.228
127.984
126.937
124.099
108.960
49.708
42.800
26.470
23.052
Cl
S
O
N
3f
f1 (ppm)

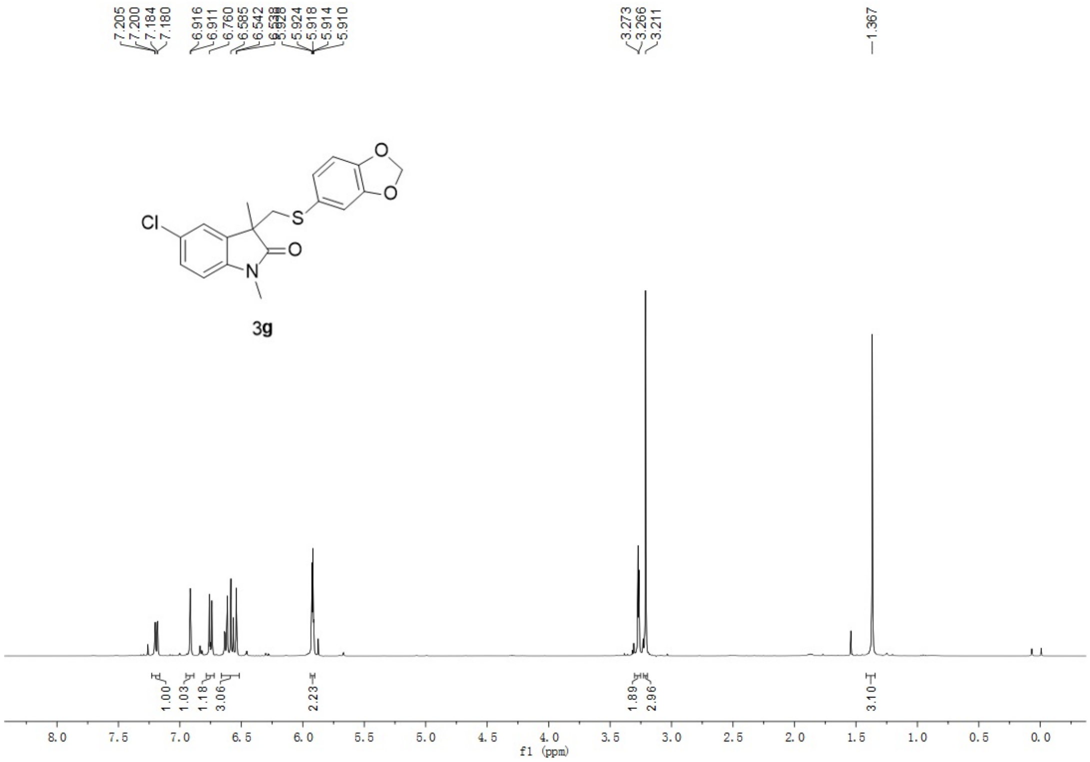
7.205
7.200
7.184
7.180
6.916
6.911
6.760
6.585
6.542
5.924
5.918
5.914
5.910
3.273
3.266
3.211
1.367
Cl
S
O
O
O
N
3g
1.00
1.03
1.18
3.06
2.23
1.89
2.96
3.10
8.0
7.5
7.0
6.5
6.0
5.5
5.0
4.5
4.0
3.5
3.0
2.5
2.0
1.5
1.0
0.5
0.0
f1 (ppm)

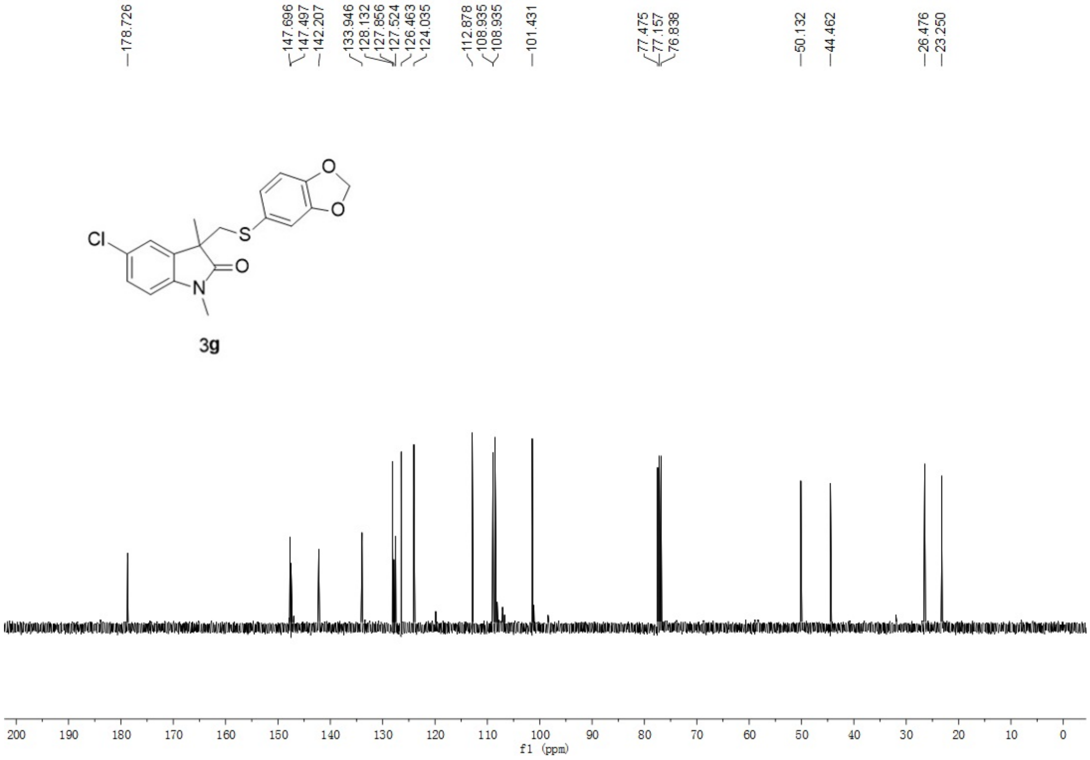
178.726
147.696
147.497
142.207
133.946
128.132
127.856
127.524
126.463
124.035
112.878
108.935
108.935
101.431
77.475
77.157
76.838
50.132
44.462
26.476
23.250
Cl
S
O
O
O
N
3g
200
190
180
170
160
150
140
130
120
110
100
90
80
70
60
50
40
30
20
10
0
f1 (ppm)

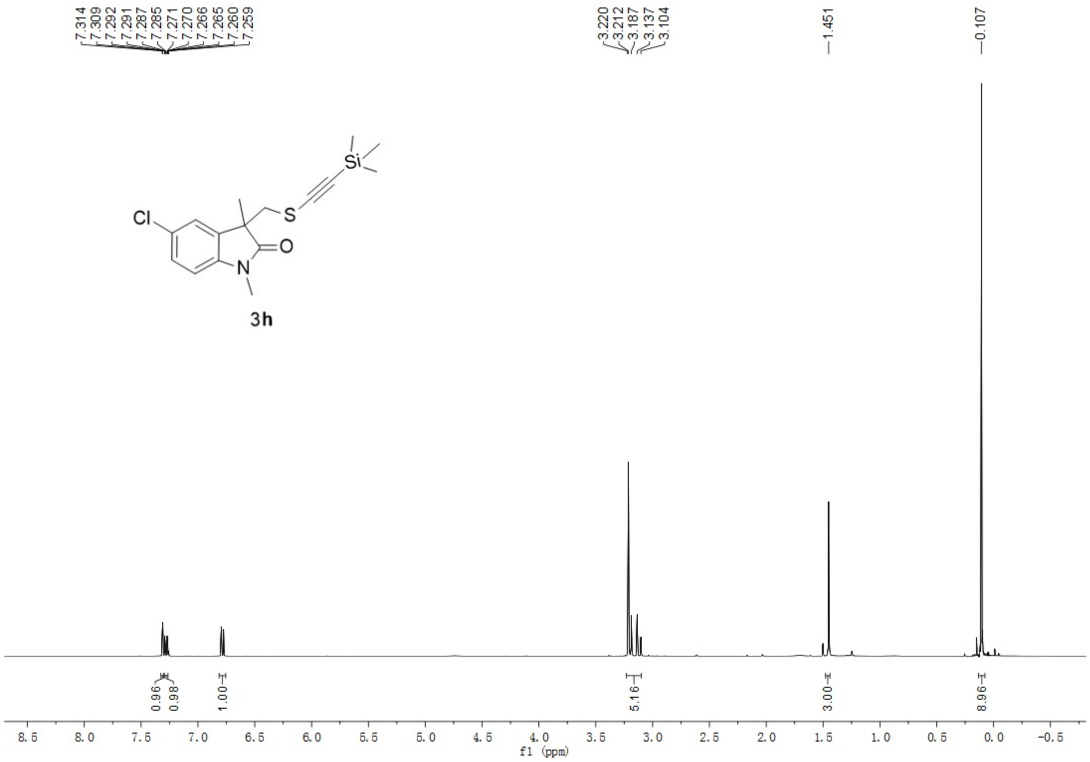
7.314 7.309 7.292 7.291 7.287 7.285 7.271 7.270 7.266 7.265 7.260 7.259
3.220 3.212 3.187 3.137 3.104
1.451
0.107
Cl
S
Si
O
N
3h
0.96
0.98
1.00
5.16
3.00
8.96
f1 (ppm)

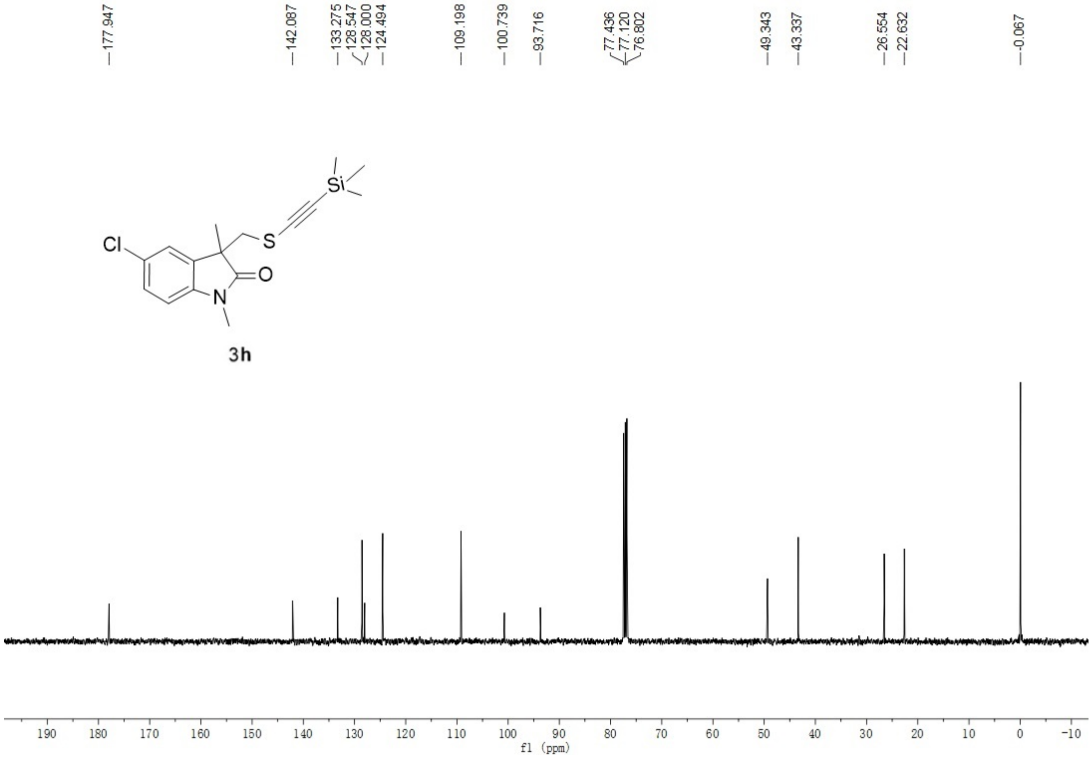
177.947
142.087
133.275 128.547 128.000 124.494
109.198
100.739
93.716
77.436 77.120 76.802
49.343
43.337
26.554 22.632
-0.067
Cl
S
Si
O
N
3h
f1 (ppm)

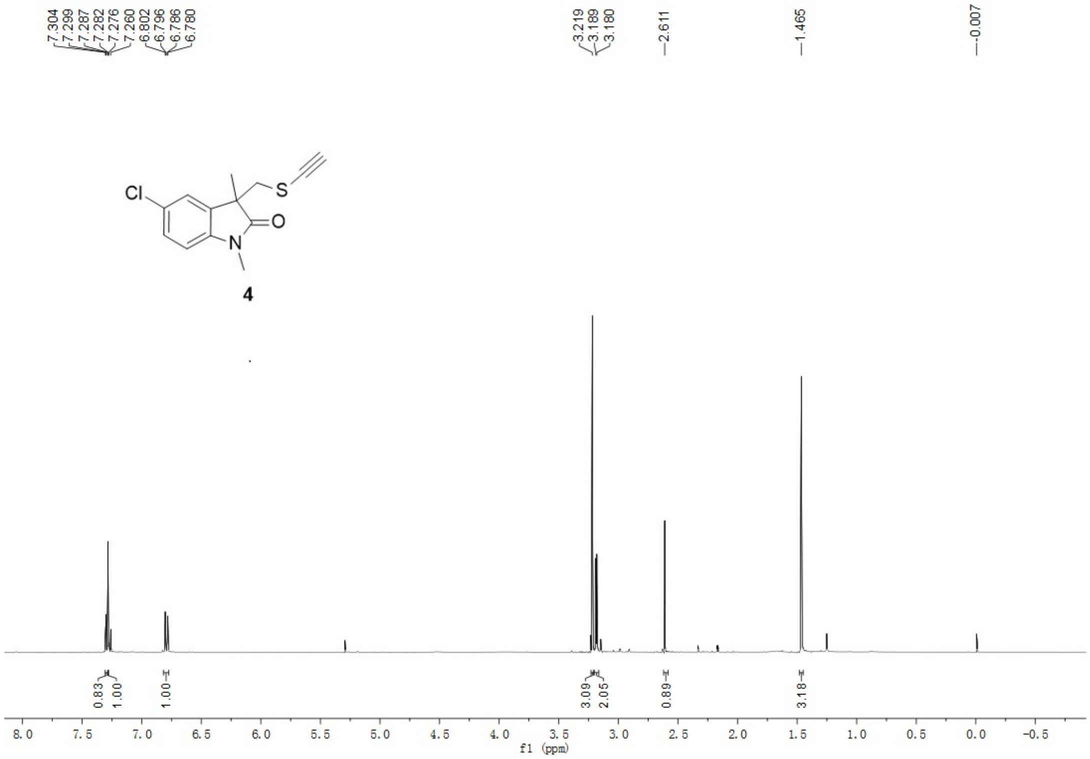
7.304
7.299
7.287
7.282
7.276
7.260
6.802
6.796
6.786
6.780
3.219
3.189
3.180
2.611
1.465
-0.007
Cl
S
O
N
4
0.83
1.00
1.00
3.09
2.05
0.89
3.18
f1 (ppm)

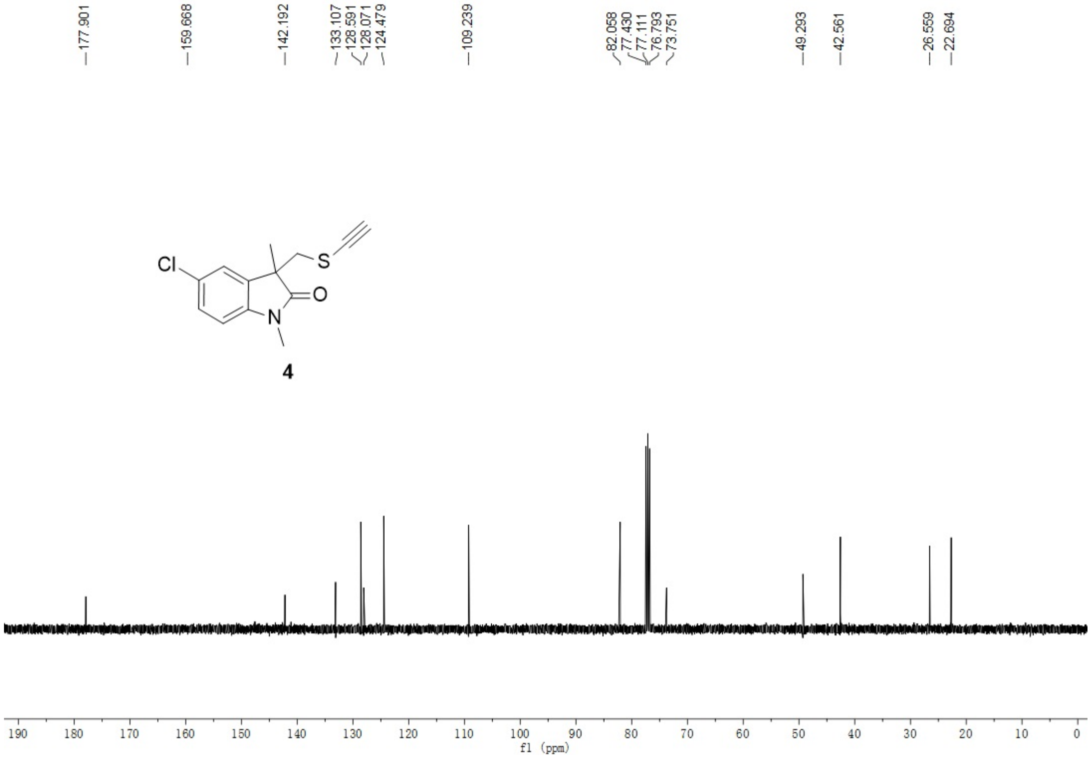
177.901
159.668
142.192
133.107
128.591
128.071
124.479
109.239
82.058
77.430
77.111
76.793
73.751
49.293
42.561
26.559
22.694
Cl
S
O
N
4
f1 (ppm)

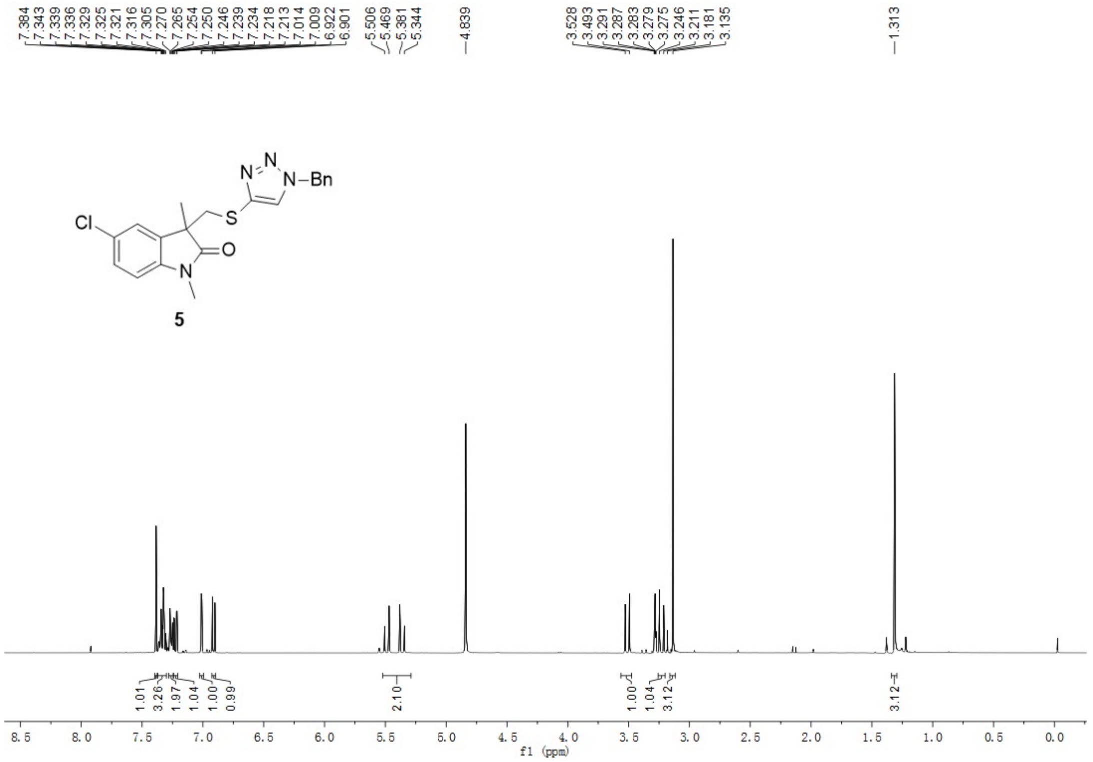
7.384
7.343
7.339
7.336
7.329
7.325
7.321
7.316
7.305
7.270
7.265
7.254
7.250
7.246
7.239
7.234
7.218
7.213
7.014
7.009
6.922
6.901
5.506
5.469
5.381
5.344
4.839
3.528
3.493
3.291
3.287
3.283
3.279
3.275
3.246
3.211
3.181
3.135
1.313
N
N
N–Bn
S
Cl
O
N
5
1.01
3.26
1.97
1.04
1.00
0.99
2.10
1.00
1.04
3.12
3.12
8.5 8.0 7.5 7.0 6.5 6.0 5.5 5.0 4.5 4.0 3.5 3.0 2.5 2.0 1.5 1.0 0.5 0.0
f1 (ppm)

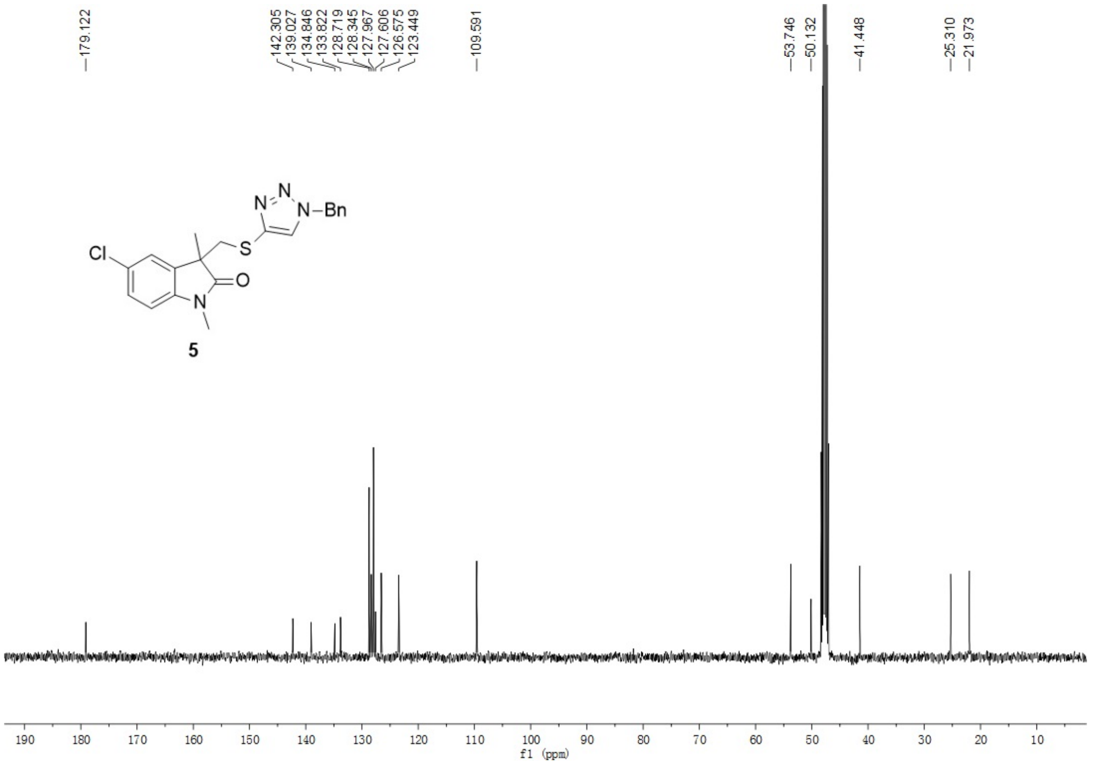
179.122
142.305
139.027
134.846
133.822
128.719
128.345
127.967
127.606
126.575
123.449
109.591
53.746
50.132
41.448
25.310
21.973
N
N
N–Bn
S
Cl
O
N
5
190 180 170 160 150 140 130 120 110 100 90 80 70 60 50 40 30 20 10
f1 (ppm)

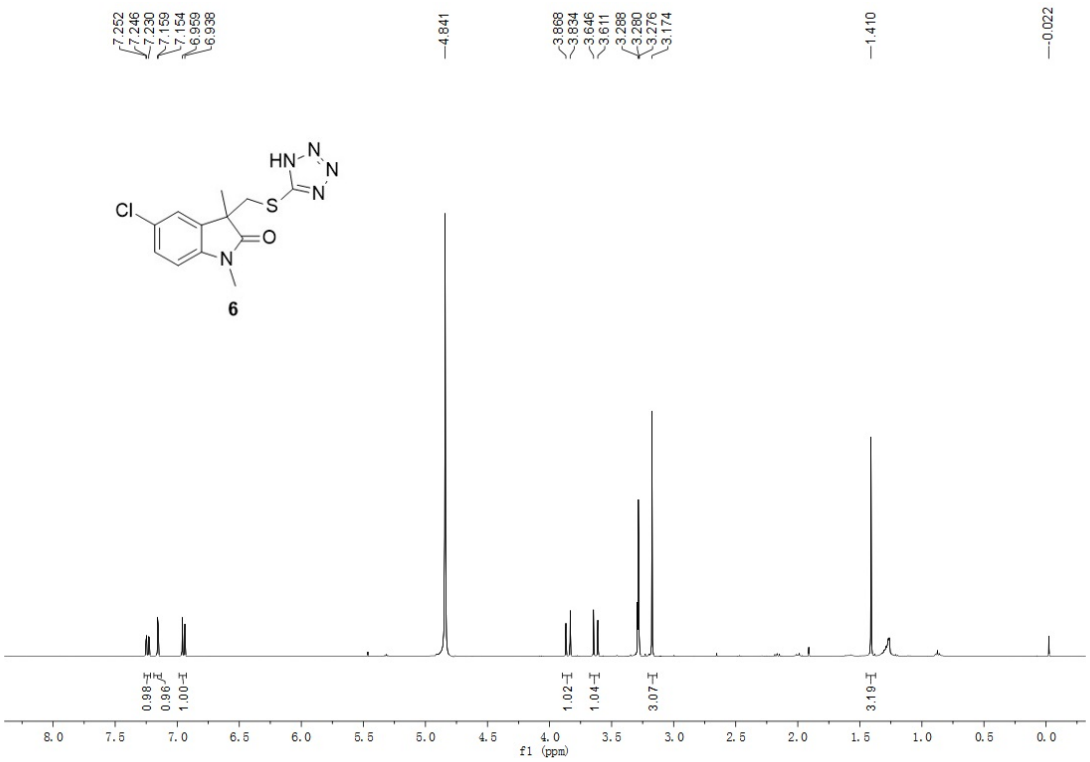
7.252
7.246
7.230
7.159
7.154
6.959
6.938
4.841
3.868
3.834
3.646
3.611
3.288
3.280
3.276
3.174
1.410
-0.022
HN
N
N
N
S
Cl
O
N
6
0.98
0.96
1.00
1.02
1.04
3.07
3.19
8.0
7.5
7.0
6.5
6.0
5.5
5.0
4.5
4.0
3.5
3.0
2.5
2.0
1.5
1.0
0.5
0.0
f1 (ppm)

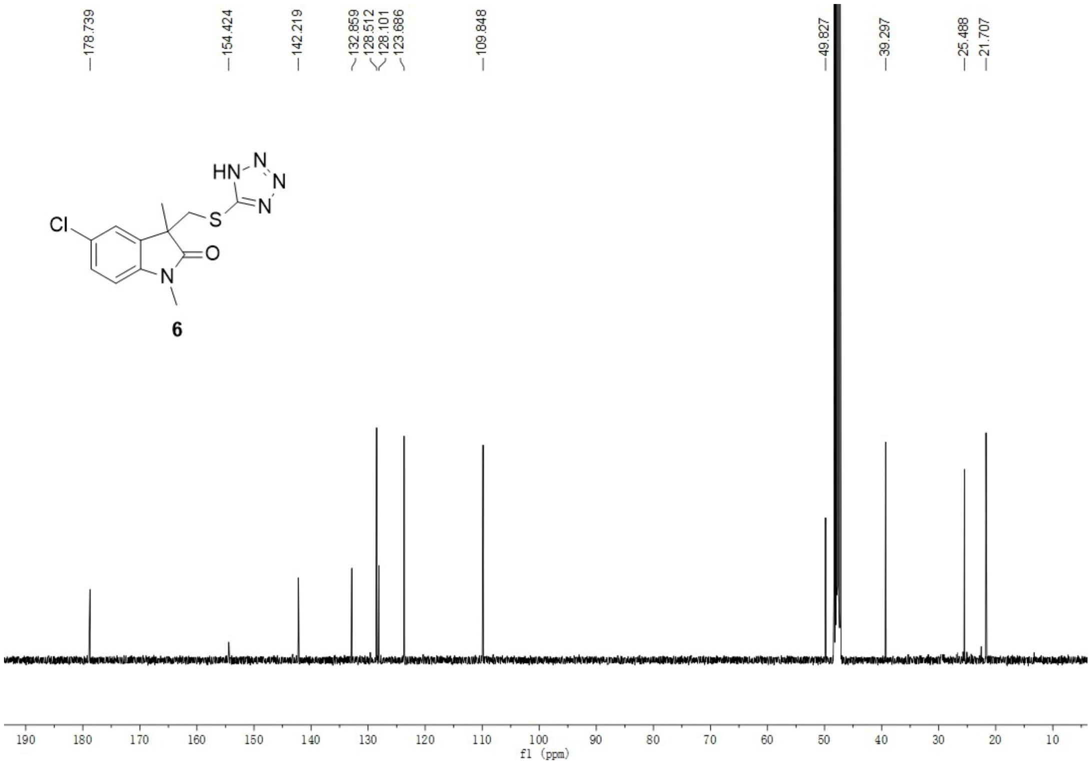
178.739
154.424
142.219
132.859
128.512
128.101
123.686
109.848
49.827
39.297
25.488
21.707
HN
N
N
N
S
Cl
O
N
6
190
180
170
160
150
140
130
120
110
100
90
80
70
60
50
40
30
20
10
f1 (ppm)

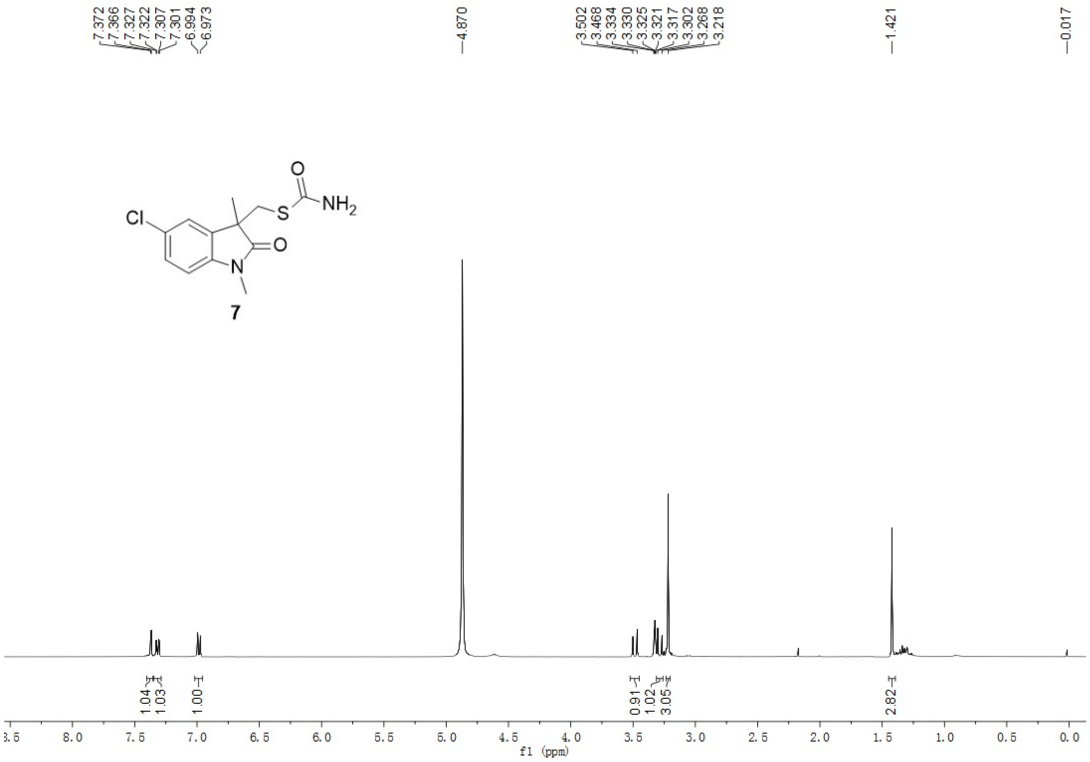
7.372
7.366
7.327
7.322
7.307
7.301
6.994
6.973
4.870
3.502
3.468
3.334
3.330
3.325
3.321
3.317
3.302
3.268
3.218
1.421
0.017
Cl
O
NH2
S
O
N
7
1.04
1.03
1.00
0.91
1.02
3.05
2.82
f1 (ppm)

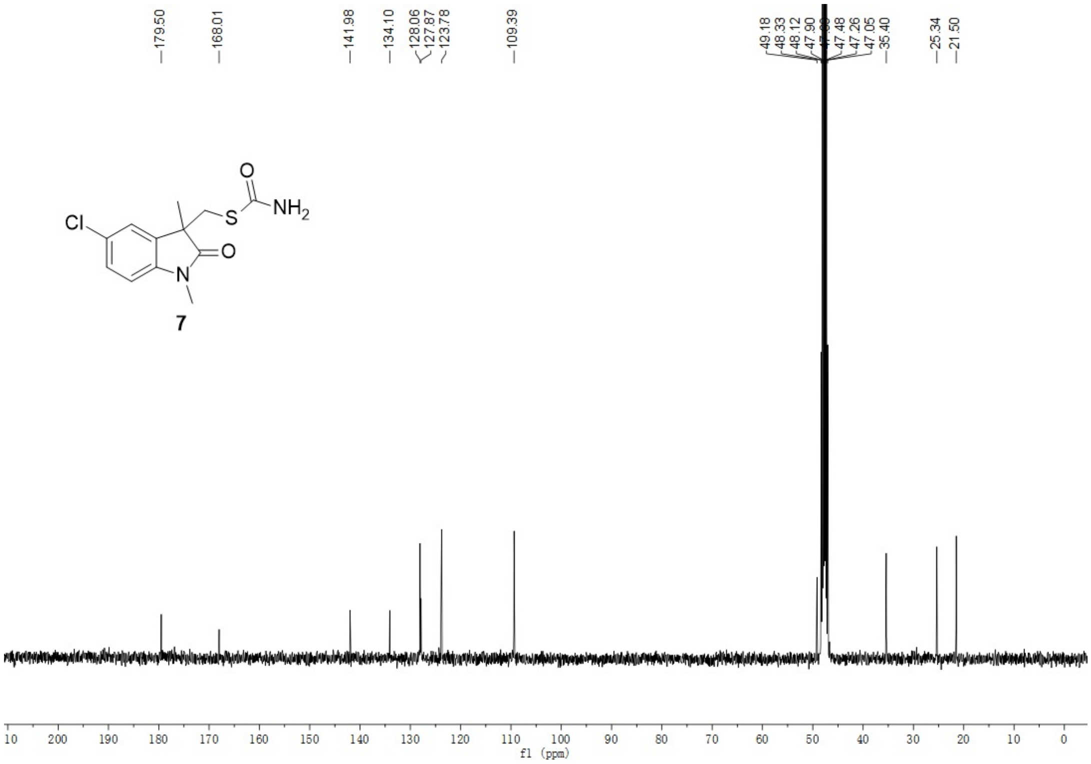
179.50
168.01
141.98
134.10
128.06
127.87
123.78
109.39
49.18
48.33
48.12
47.90
47.48
47.26
47.05
35.40
25.34
21.50
Cl
O
NH2
S
O
N
7
f1 (ppm)

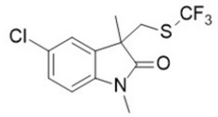
8

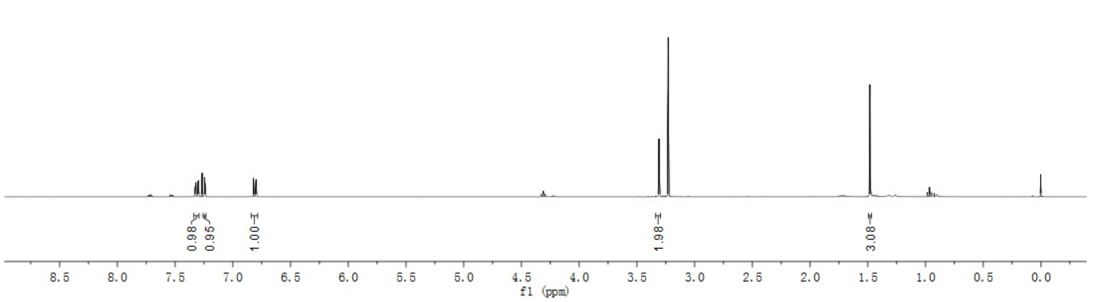

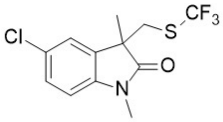
8

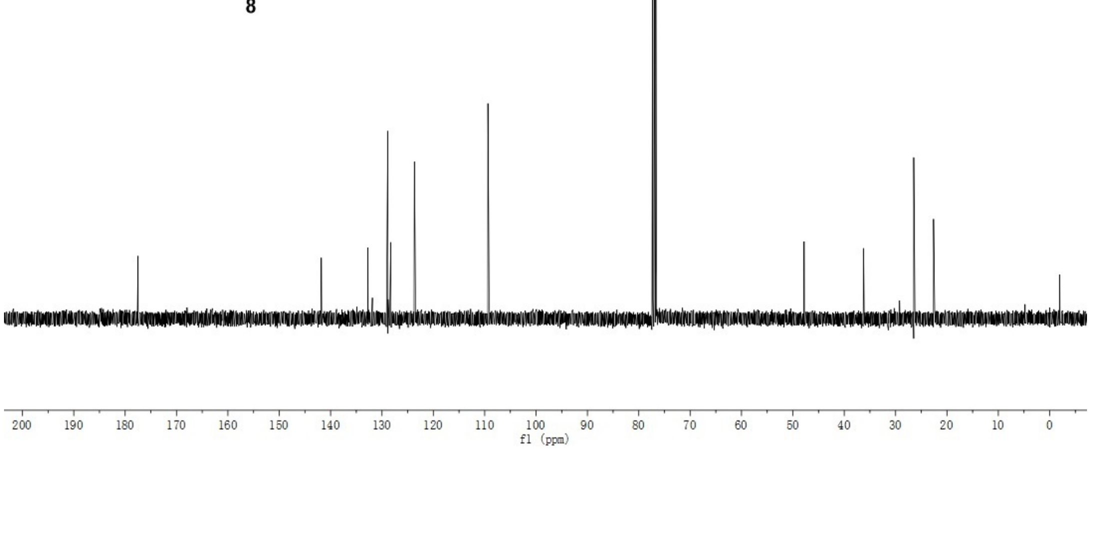

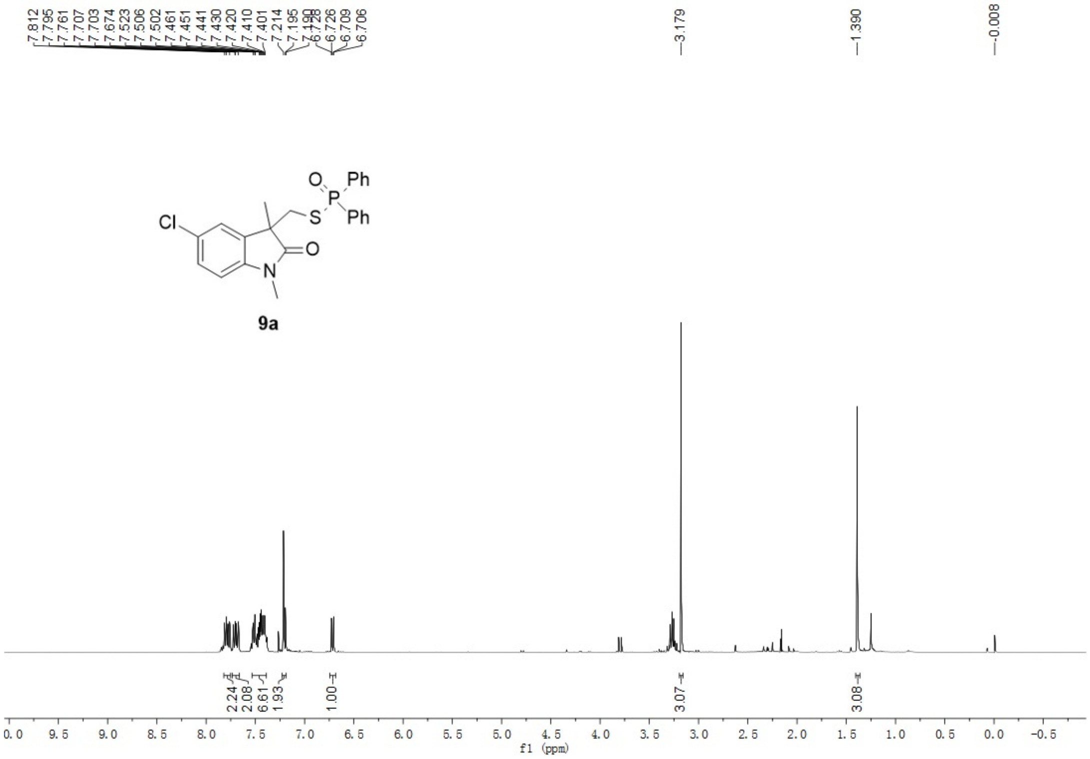
7.812
7.795
7.761
7.707
7.703
7.674
7.523
7.506
7.502
7.461
7.451
7.441
7.430
7.420
7.410
7.401
7.214
7.195
6.726
6.709
6.706
3.179
1.390
-0.008
O
P
Ph
Ph
S
Cl
O
N
9a
2.24
2.08
6.61
1.93
1.00
3.07
3.08
f1 (ppm)

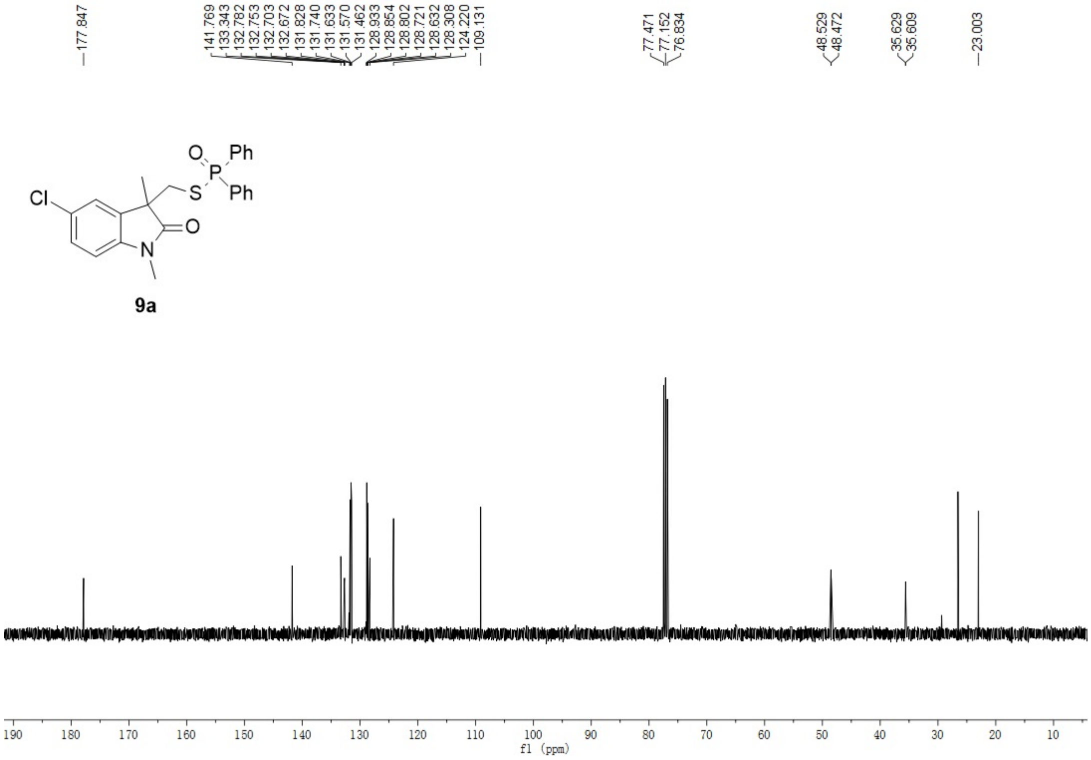
177.847
141.769
133.343
132.782
132.753
132.703
132.672
131.828
131.740
131.633
131.570
131.462
128.933
128.854
128.802
128.721
128.632
128.308
124.220
109.131
77.471
77.152
76.834
48.529
48.472
35.629
35.609
23.003
O
P
Ph
Ph
S
Cl
O
N
9a
f1 (ppm)

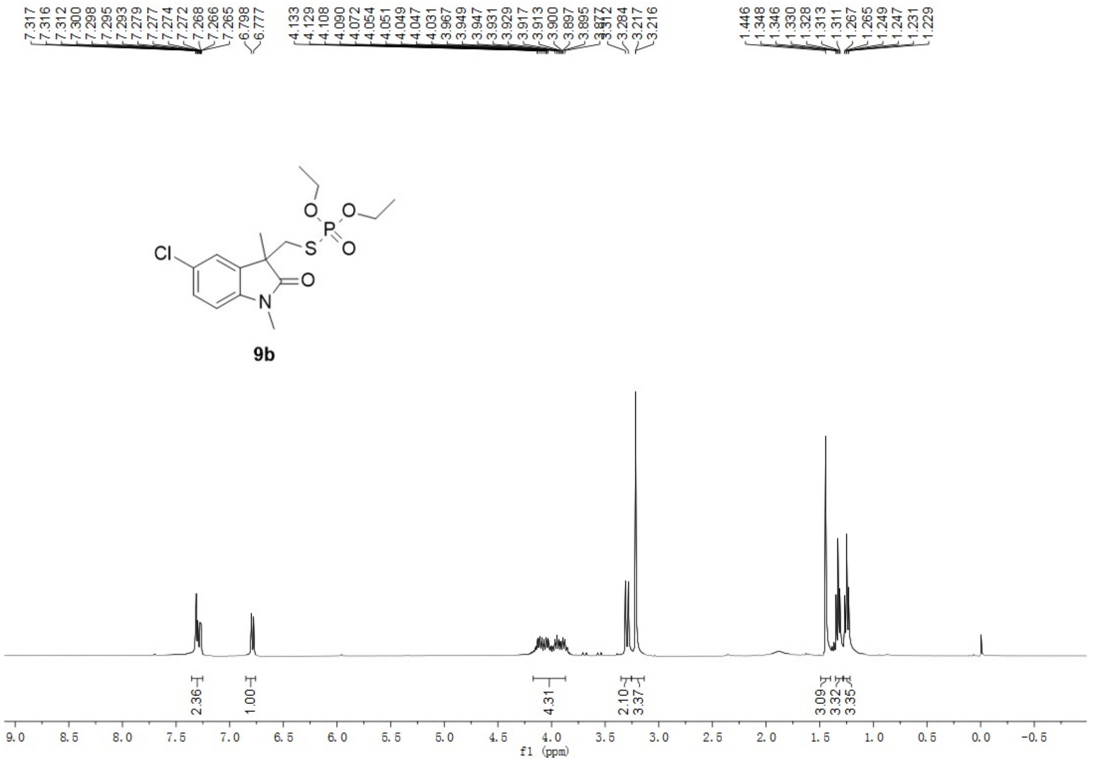

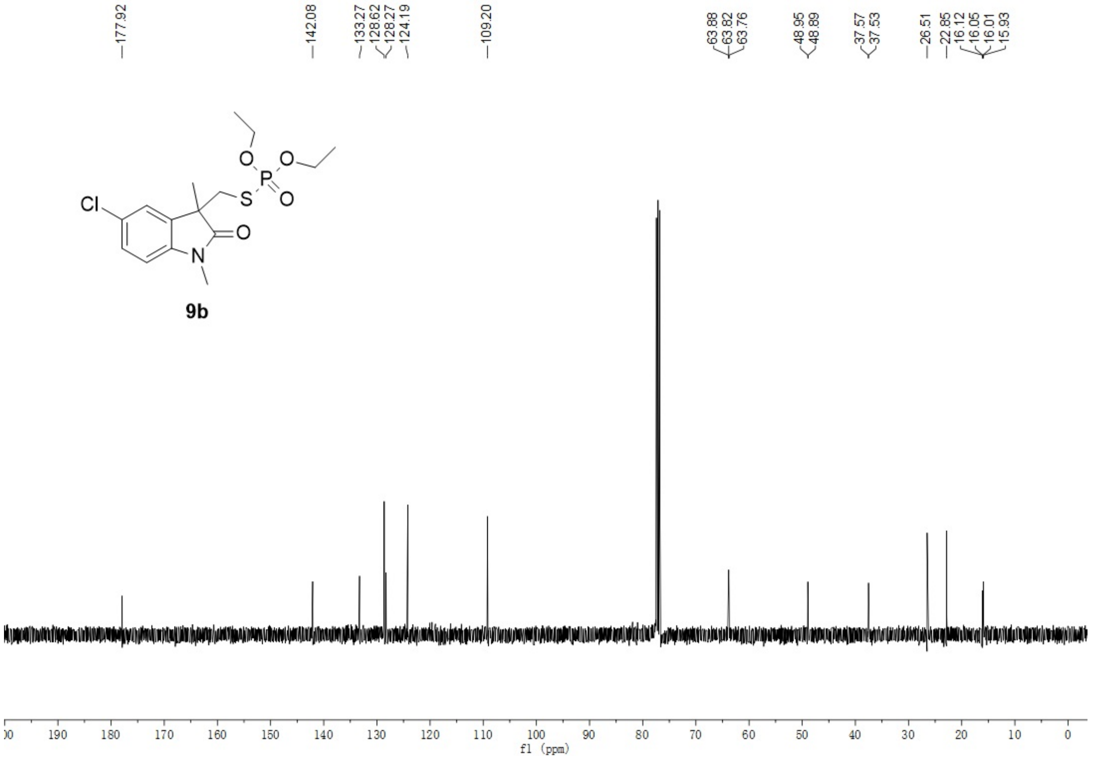

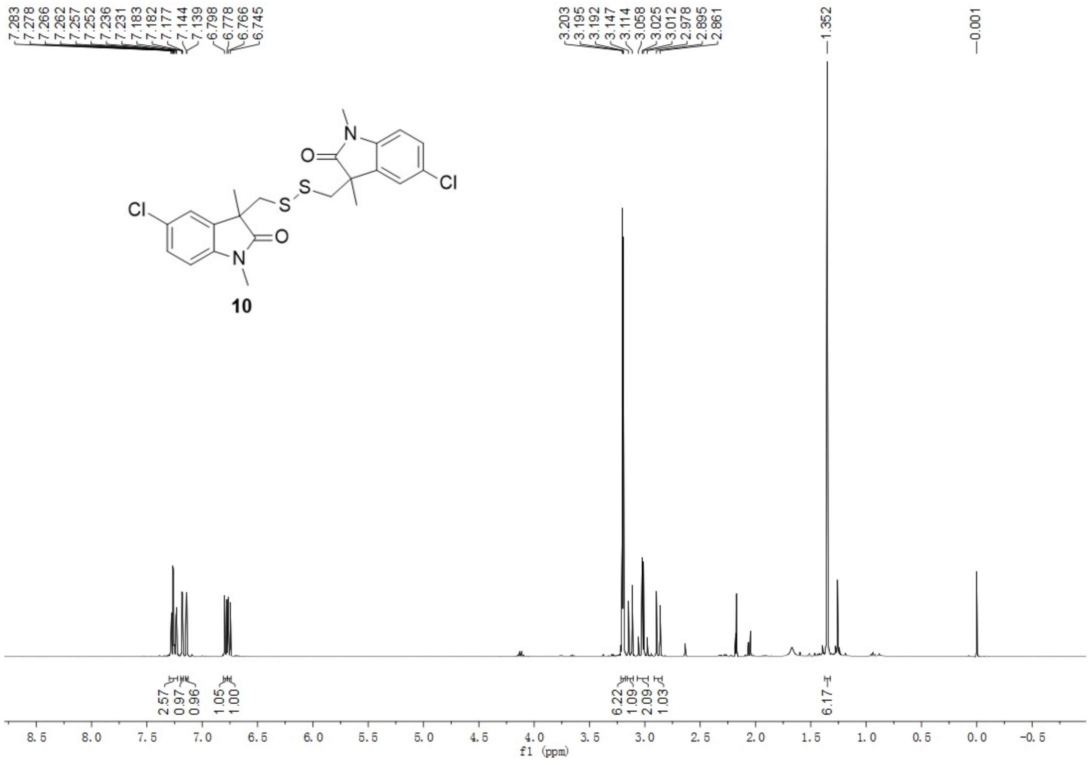

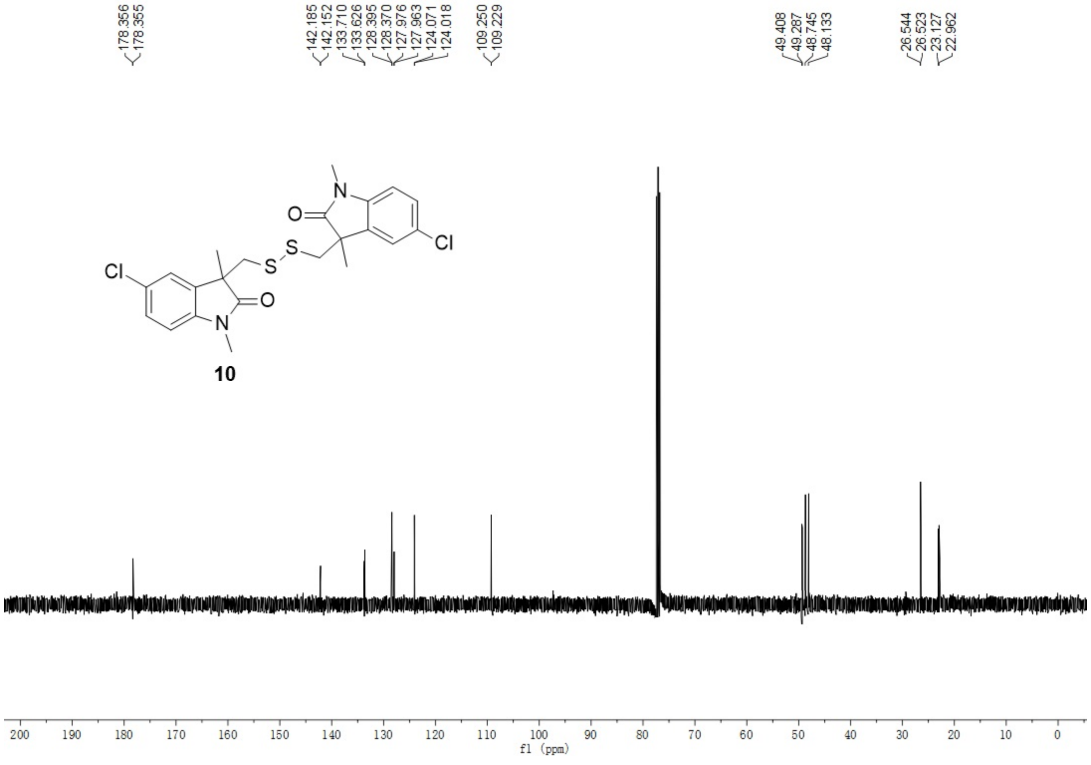

Supplement: Supplementary file 1 [file molecules-23-02727-s001.pdf]
